# Nucleophilic and electrophilic cyclization of *N*-alkyne-substituted pyrrole derivatives: Synthesis of pyrrolopyrazinone, pyrrolotriazinone, and pyrrolooxazinone moieties

**DOI:** 10.3762/bjoc.13.83

**Published:** 2017-05-04

**Authors:** Işıl Yenice, Sinan Basceken, Metin Balci

**Affiliations:** 1 Department of Chemistry, Middle East Technical University, 06800 Ankara, Turkey; 2 Department of Chemistry, Hitit University, 19030 Corum, Turkey

**Keywords:** alkyne cyclization, pyrrole, pyrrolooxazinone, pyrrolopyrazinone, pyrrolotriazinone

## Abstract

Intramolecular nucleophilic and electrophilic cyclization of *N-*alkyne-substituted pyrrole esters is described. Efficient routes towards the synthesis of pyrrolopyrazinone, pyrrolotriazinone and pyrrolooxazinone have been developed. First, *N*-alkyne-substituted pyrrole ester derivatives were synthesized. Introduction of various substituents into the alkyne functionality was accomplished by a copper-catalyzed cross-coupling reaction. Nucleophilic cyclization of *N*-alkyne-substituted methyl 1*H*-pyrrole-2-carboxylates with hydrazine afforded the 6-*exo*-dig/6-*endo-*dig cyclization products depending on the electronic nature of the substituents attached to the alkyne. On the other hand, cyclization of *N*-alkyne-substituted methyl 1*H*-pyrrole-2-carboxylates with iodine only resulted in the formation of the 6-*endo*-dig cyclization product regardless of the substitution of the alkyne functionality.

## Introduction

**Figure 1:**
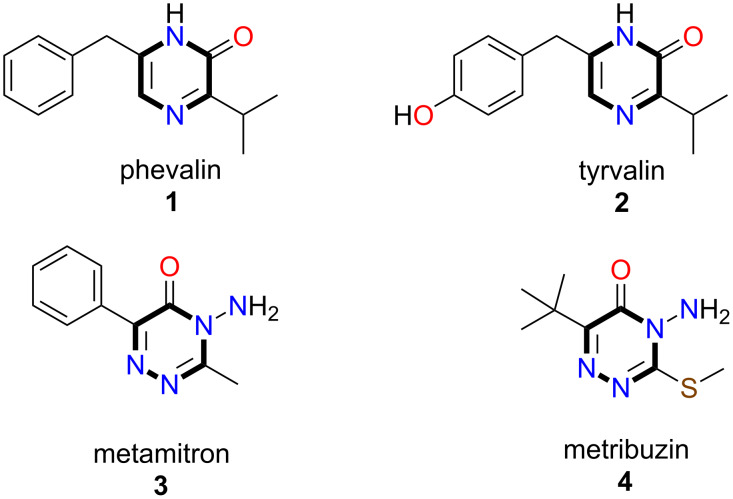
Structures of some natural products containing pyrazinone and aminotriazonone skeletons.

Pyrrole has a great range of applications in organic synthesis because of its occurrence in many active natural products, synthetic pharmaceuticals, and optoelectronic materials [1–2]. The pyrazinones derived from the pyrazine ring by single oxidation of one carbon atom also show very important biological activities. Various microbes, reported in the literature, can accomplish the synthesis of pyrazinone derivatives [3–6]. For instance, phevalin (**1**) and tyrvalin (**2**), pyrazinone derivatives synthesized by a serious human pathogen, *Staphylococcus aureus,* act as protein kinase inhibitors (Figure 1) [7–8].

Triazinone heterocycles are essential in organic synthesis regarding their herbicide, anticancer, antimicrobial, and antimetastatic activities. Metamitron (**3**) and metribuzin (**4**), 1,2,4-triazinone herbicides having an amino group, are absorbed by the roots of plants and inhibit photosynthesis by inhibiting electron transport (Figure 1). They are used to control grasses pre- and post-emergence [9–10].

**Figure 2:**
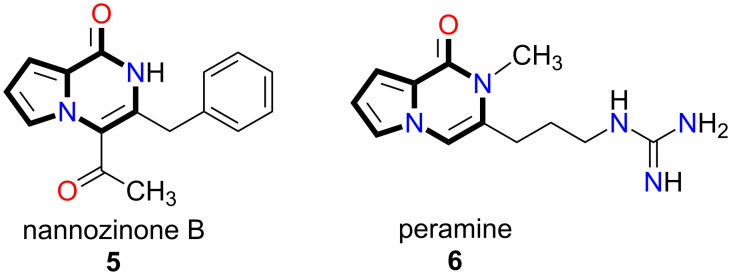
Structures of some natural products containing a pyrrolopyrazinone moiety.

Pyrrole-fused pyrazinone heterocycles and their synthesis are of great interest due to their natural presence and potent pharmacological and biological activities. A natural product, nannozinone B (**5**, Figure 2), containing a pyrrolopyrazinone moiety was isolated from a myxobacterium, *Nannocystis pusilla* [11]. The alkaloid peramine (**6**, Figure 2), isolated from endophyte-infected perennial ryegrass, has feeding deterrent activity against insects [12–13].

**Figure 3:**
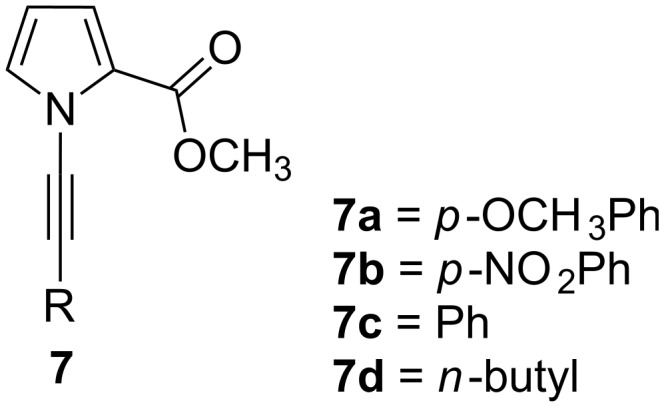
*N-*alkyne substituted pyrrole esters **7a–d**.

An efficient and straightforward synthetic method for the construction of the pyrrolopyrazinone core structure as well as for its derivatives is not described in the literature [14]. Recently, we developed new synthetic methodologies for the synthesis of various pyrrole-fused new heterocycles using alkyne cyclization reactions [15–22]. In this article, we demonstrate for the first time the concept of the cyclization of *N-*alkyne-substituted pyrrole esters **7** (Figure 3) to provide a practical access to design pyrrolopyrazinone, pyrrolotriazinone, and pyrrolooxazinone derivatives.

## Results and Discussion

**Scheme 1:**
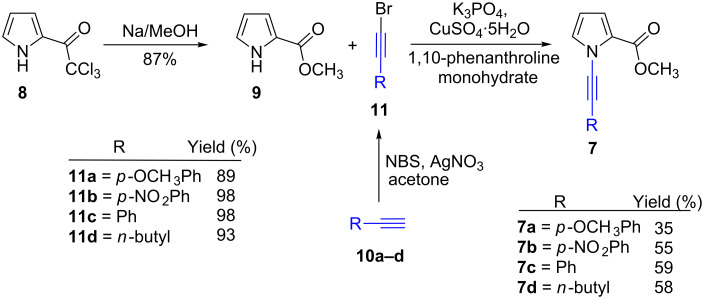
Synthesis of *N*-alkyne substituted methyl 1*H*-pyrrole-2-carboxylate derivatives **7a–d**.

The starting compound **8** was synthesized via a slightly modified route by acetylation of pyrrole with trichloroacetyl chloride in 89% yield [19,23]. The reaction of **8** with NaOMe in methanol gave pyrrole ester **9** (Scheme 1) [19,24]. It is essential to use bromoalkynes for *N*-alkyne substitution of pyrrole ester **9**. Substituted alkyne derivatives **10a** and **10b** were synthesized according to the literature. The Sonogashira coupling reaction [25] of aryl iodides with terminal acetylene is an effective approach towards the synthesis of substituted arylalkynes. The reaction of 1-iodo-4-methoxybenzene and 1-iodo-4-nitrobenzene with trimethylsilylacetylene under the Sonogashira coupling conditions followed by hydrolysis of the trimethylsilyl groups with K_2_CO_3_ resulted in the formation of **10a** and **10b** [26–28]. Fortunately, terminal alkynes can be easily converted into bromoalkynes with *N*-bromosuccinimide in the presence of silver nitrate. The synthesized acetylenes **10a,b** and commercially available acetylenes **10c,d** were converted into bromoalkyne derivatives **11a–d** according to the literature procedure (Scheme 1) [29–30].

After the successful generation of alkynyl bromides **11a–d**, the next step was the synthesis of *N*-alkyne-substituted pyrrole derivatives **7a–d**. A practical method is the coupling reaction of substituted pyrroles with alkynyl bromides using catalytic CuSO_4_·5H_2_O and 1,10-phenanthroline [31]. When alkynylation with **11a** was carried out at 85 °C, the conversion was only 12%. We assume this is due to the presence of an electron-donating group at the benzene ring. However, when the reaction was carried out at the reflux temperature of toluene, the conversion increased to 35%.

**Scheme 2:**
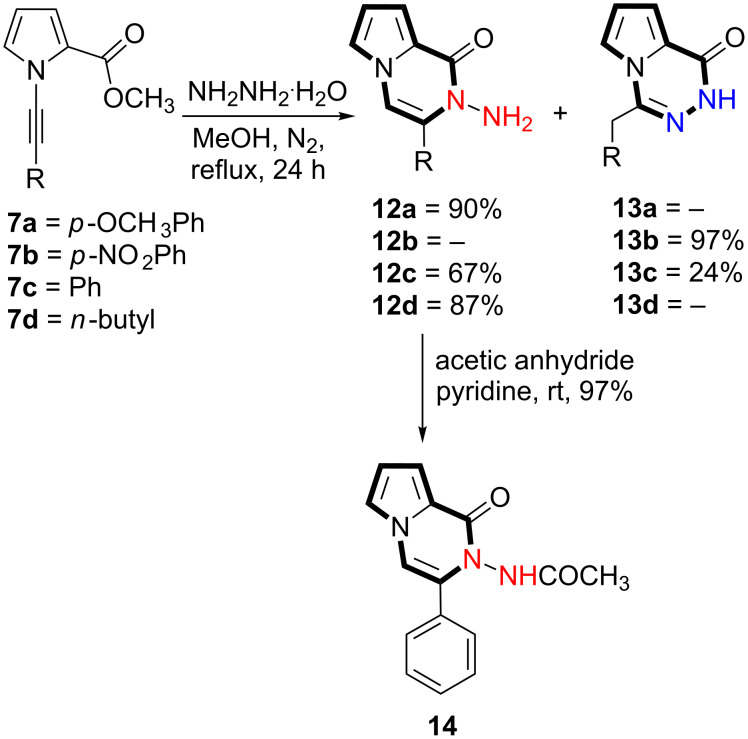
Nucleophilic cyclization reaction of compounds **7a–d** and acetylation of **12c**.

Next, we conducted cyclization reactions of *N*-alkyne substituted carboxylate derivatives **7a–d** via hydrazine monohydrate. Methyl 1-(phenylethynyl)-1*H*-pyrrole-2-carboxylate (**7c**) was treated with hydrazine monohydrate in MeOH under N_2_ atmosphere at reflux temperature. Pyrrolopyrazinone **12c** and pyrrolotriazinone **13c** skeletons were formed in 67% and 24% yields, respectively (Scheme 2).

**Figure 4:**
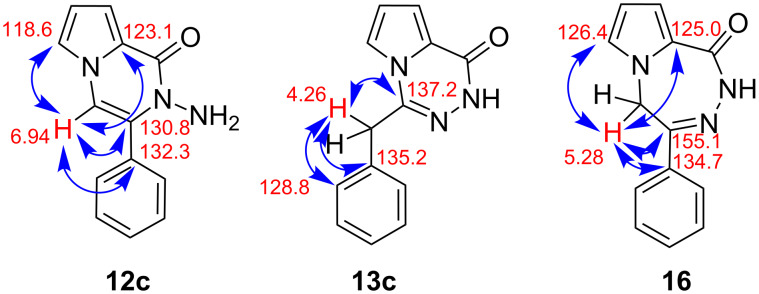
Correlations of olefinic proton in **12c** and methylene protons in **13c** and **16** with the relevant carbon atoms (from the HMBC spectrum).

The structures of cyclization products **12c** and **13c** were determined using NMR spectra. The exact locations of the olefinic carbon (=CH) in **12c** and methylene group in **13c** were determined from 2D NMR (HSQC and HMBC) spectra. The ^1^H NMR spectrum of pyrrolopyrazinone derivative **12c** shows the presence of two NH_2_ protons resonating at 4.41 ppm as a broad singlet. The double bond proton in **12c** resonating at 6.94 ppm as a singlet shows strong correlation in the HMBC spectrum with the quaternary carbon atoms resonating at 132.3, 130.8, and 123.1 as well as with the tertiary carbon atoms (=CH) at 118.6 ppm (Figure 4). This information clearly shows that the double bond (CH=C) is located between the pyrrole nitrogen atom and the benzene ring (Figure 4). On the other hand, the methylene protons in **13c** resonating at 4.26 ppm as a singlet shows strong correlation with the quaternary carbon atoms resonating at 137.2 and 135.2 as well as with the aromatic *o*-carbon atoms at 128.8 ppm indicating the structure **13c**.

**Figure 5:**
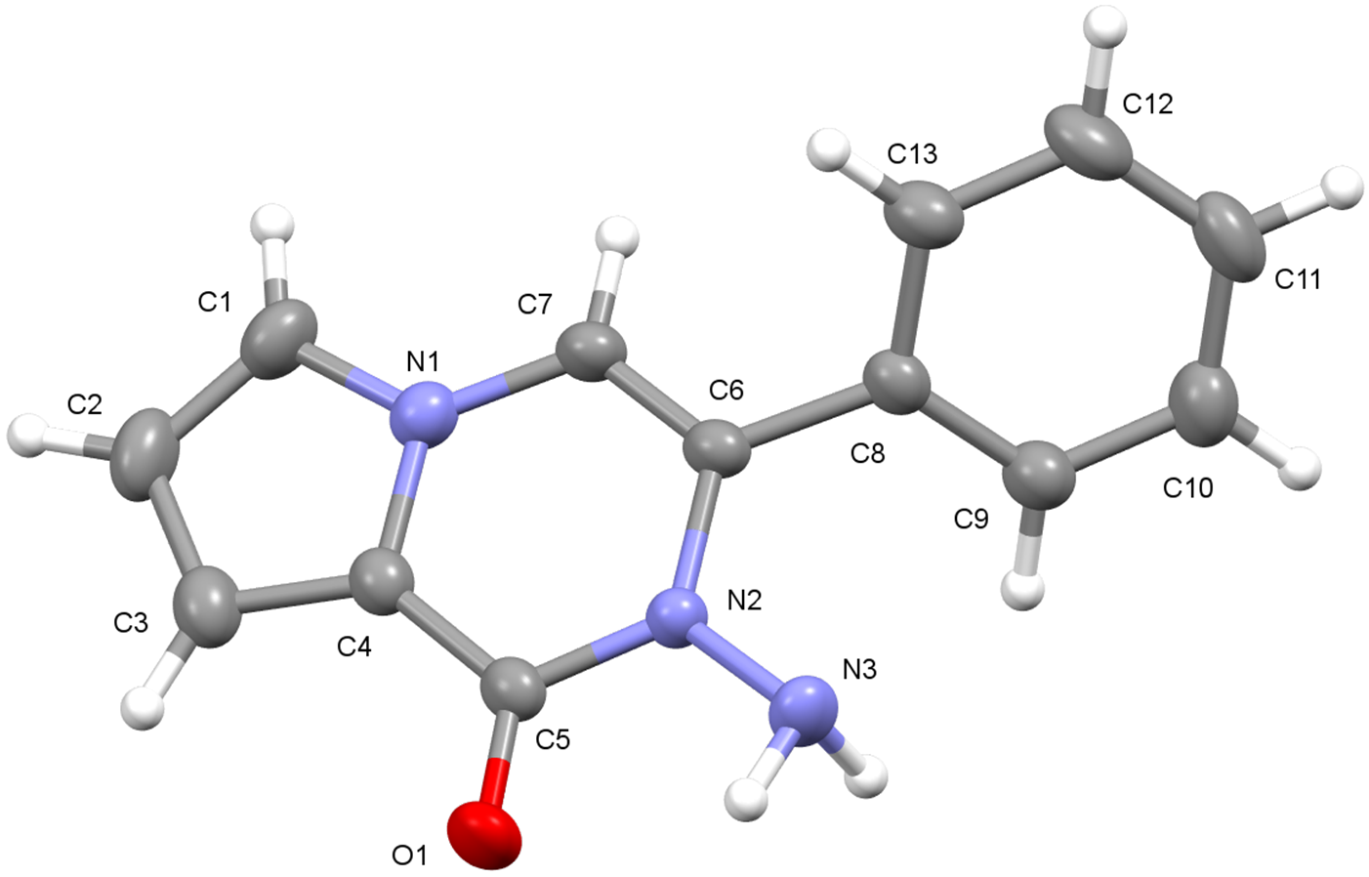
Single-crystal X-ray structure of **12c** shown with 40% probability displacement ellipsoids.

For further proof of the structure, **12c** was submitted to an acetylation reaction with acetic anhydride in pyridine to give the acetylated compound **14** in 97% yield (Scheme 2). The NH- proton resonance was now shifted to lower field (9.12 ppm) as expected. Finally, the structure of **12c** was further confirmed by single-crystal X-ray analysis (Figure 5) [32].

**Scheme 3:**
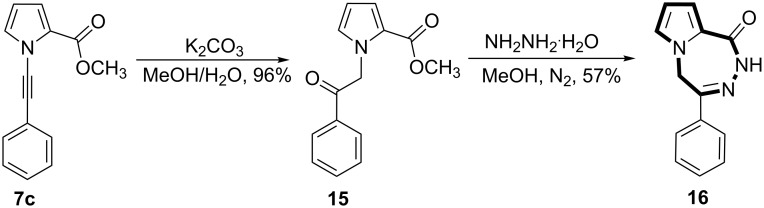
Synthesis of **16**.

The compound **13c** could have the structure **16**. However, in a structure like **16** one would expect a correlation between the methylene protons and the pyrrole ring carbon atoms. As we were not able to observe such correlations in the HMBC spectrum we eliminated this structure. To assign the correct structure to the product **13c**, we decided to synthesize **16** using a different approach and to compare the NMR spectra of **13c** with those of **16**. For this reason, **7c** was first reacted with potassium carbonate in MeOH/H_2_O solution to give the ketone **15**. Treatment of **15** with hydrazine monohydrate in methanol gave the expected product **16** in 57% yield (Scheme 3).

The ^1^H NMR and ^13^C NMR spectra of these two compounds **13c** and **16** were completely different from each other. The methylene protons in **16** resonating at 5.28 ppm showed strong correlations with the imine carbon atom, the *ipso*-carbon atom and two α-carbon atoms of the pyrrole ring, clearly indicating that the methylene group is incorporated into the seven-membered ring. All this information shows that the methylene group is located between the pyrrole ring and the imine double bond.

With these encouraging results in hand, we embarked on the evaluation of the substrate scope for this useful transformation. The compounds **7a**, **7b**, and **7d** were submitted to a cyclization reaction under the same reaction conditions applied to compound **7c**. As shown in Scheme 2, compounds **7a** and **7d** formed 2-aminopyrrolopyrazinone derivatives **12a** and **12d**, whereas **7b** furnished pyrrolotriazinone derivative **13b**. The electronic nature of the substituents attached to the alkyne determines the mode of the cyclization reaction. Since the electron-donating methoxy group increases the electron density at the alkyne unit, cyclization takes place with the less nucleophilic nitrogen atom of the hydrazide group. On the other hand, the nitro group decreases the electron density, making the alkyne carbon atom next to the pyrrole nitrogen atom electropositive, and cyclization takes place with the terminal nitrogen atom forming a six-membered ring. Moreover, the *n-*butyl-substituted alkyne carbon atom undergoes a reaction to form 6*-endo-*dig cyclization product **12d**.

**Figure 6:**
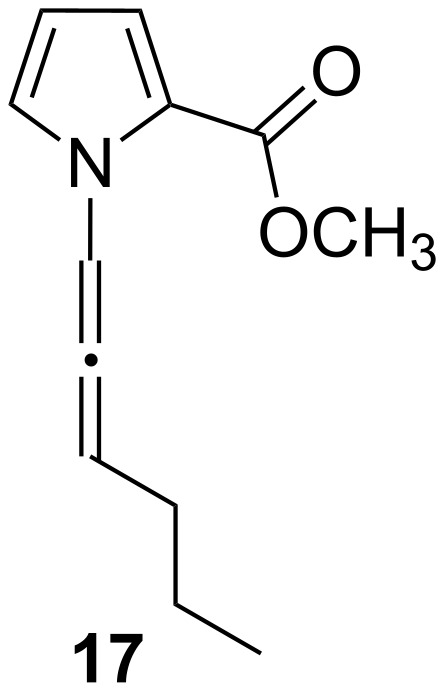
The structure of allene **17** formed during the reaction of **7d** with a base.

We assume that an allenic intermediate **17** (Figure 6) formed during the reaction is responsible for exclusive formation of **12d**. Since the central carbon atom of an allene unit is more electropositive, the carbon atom can undergo an attack by the amide nitrogen atom to form a six-membered ring. In the case of **7a–c,** the formation of allenic intermediates is out of question [15].

**Scheme 4:**
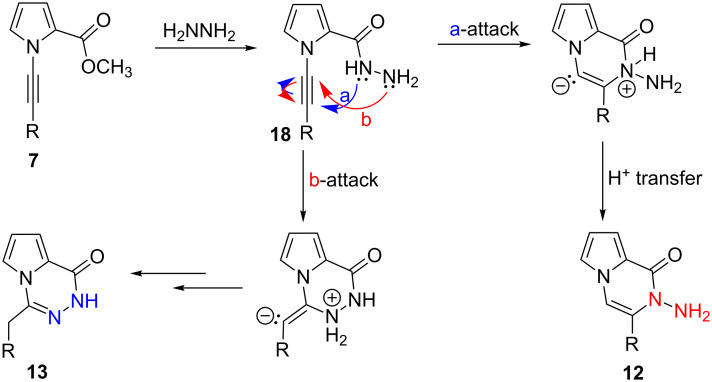
Proposed reaction mechanism of nucleophilic cyclization reaction of **7**.

Based on our experimental results, we proposed the mechanism outlined in Scheme 4 for the formation of compounds **12** and **13**. The first step is the formation of hydrazide **18**. The electronic properties of the substituents designate the fate of the reaction at this step. The intermediate undergoes either 6-*endo-*dig or 6-*exo*-dig cyclizations to form the corresponding products by attack of lone pair electrons of the internal nitrogen or terminal nitrogen atoms, respectively. The 6-*endo*-dig cyclization follows only a proton shift to yield pyrrolopyrazinone skeleton **12**, while 6-*exo*-dig follows first a proton shift and then an [1,3]-hydride shift to afford pyrrolotriazinone moiety **13**.

**Scheme 5:**
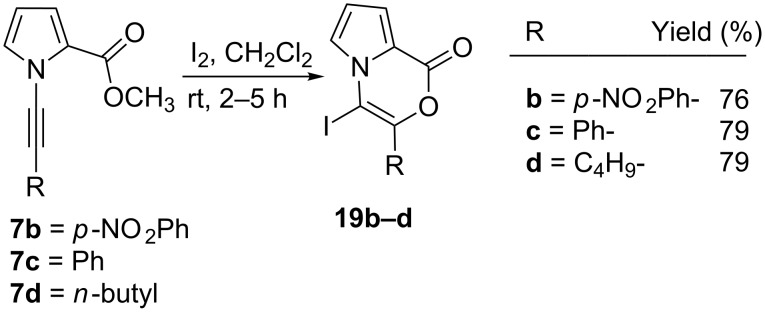
Electrophilic cyclization reactions of **19a–c** with iodine.

After completion of the nucleophilic cyclization reactions, we desired to activate the triple bond with iodine to synthesize pyrrolooxazinone derivatives via an electrophilic intramolecular cyclization reaction. For this purpose, *N*-alkyne-substituted methyl 1*H*-pyrrole-2-carboxylate derivatives **7b–d** were treated with iodine in dichloromethane to yield the corresponding pyrrolooxazinone derivatives **19b–d** in yields of 76–79% (Scheme 5).

**Figure 7:**
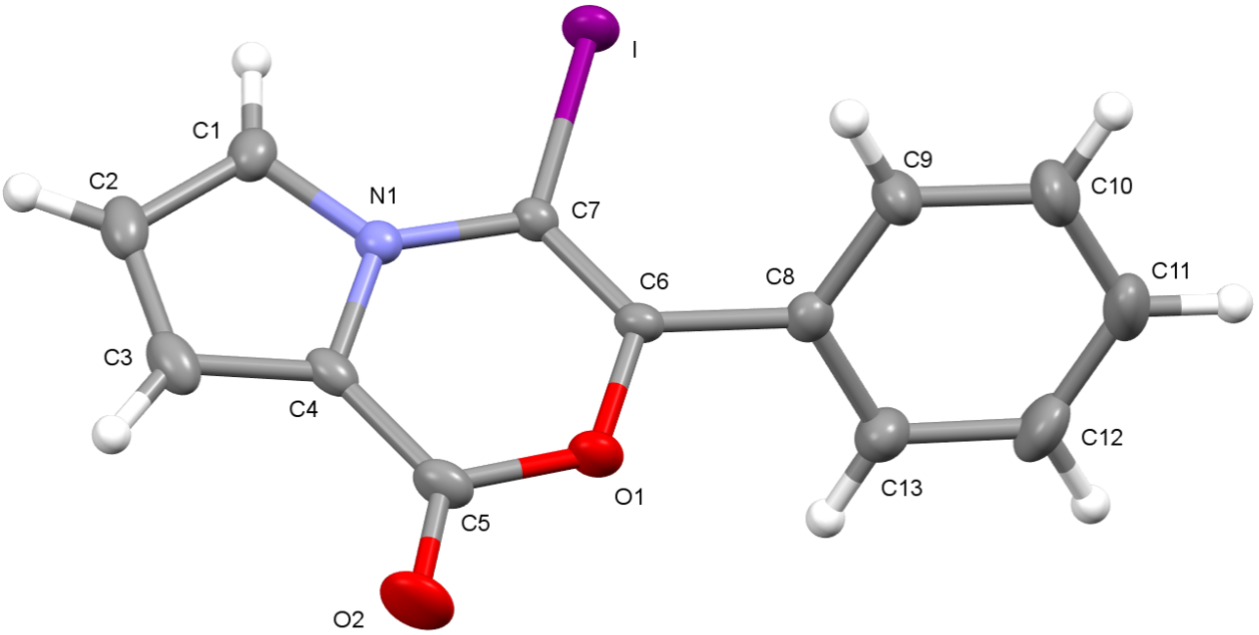
Single-crystal X-ray structure of **19c** shown with 40% probability displacement ellipsoids.

Spectral data of **19b–d** were in complete agreement with the proposed structures. The structure of **19c** was further confirmed by single-crystal X-ray analysis (Figure 7) [32].

**Scheme 6:**
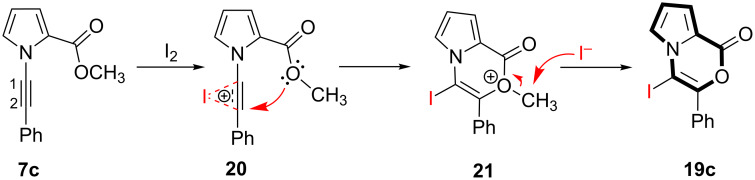
Proposed reaction mechanism of electrophilic cyclization reaction of **7c**.

The following reaction mechanism was proposed for the formation of **19c** (Scheme 6). The reaction starts with the π-activation of the triple bond by iodine to form the intermediate **20**, which undergoes an intramolecular addition of the ester oxygen atom to the alkyne functionality to form the intermediate **21**. In the next step, a nucleophilic attack on the methyl group by iodide forms the product **19c**.

All cyclization reactions of **7b–d** with iodine underwent a 6-*endo-*dig cyclization. No 5-*exo*-dig cyclization was observed. To address this issue we performed some DFT calculations. Geometrical parameters of reactants, transition states (TS), and products were fully optimized in dichloromethane with the M06 method using the GEN basis set combination 6-31+G(d) and LANL2DZ in the Gaussian 09 software package [33]. Computational details are given in Supporting Information File 1.

**Figure 8:**
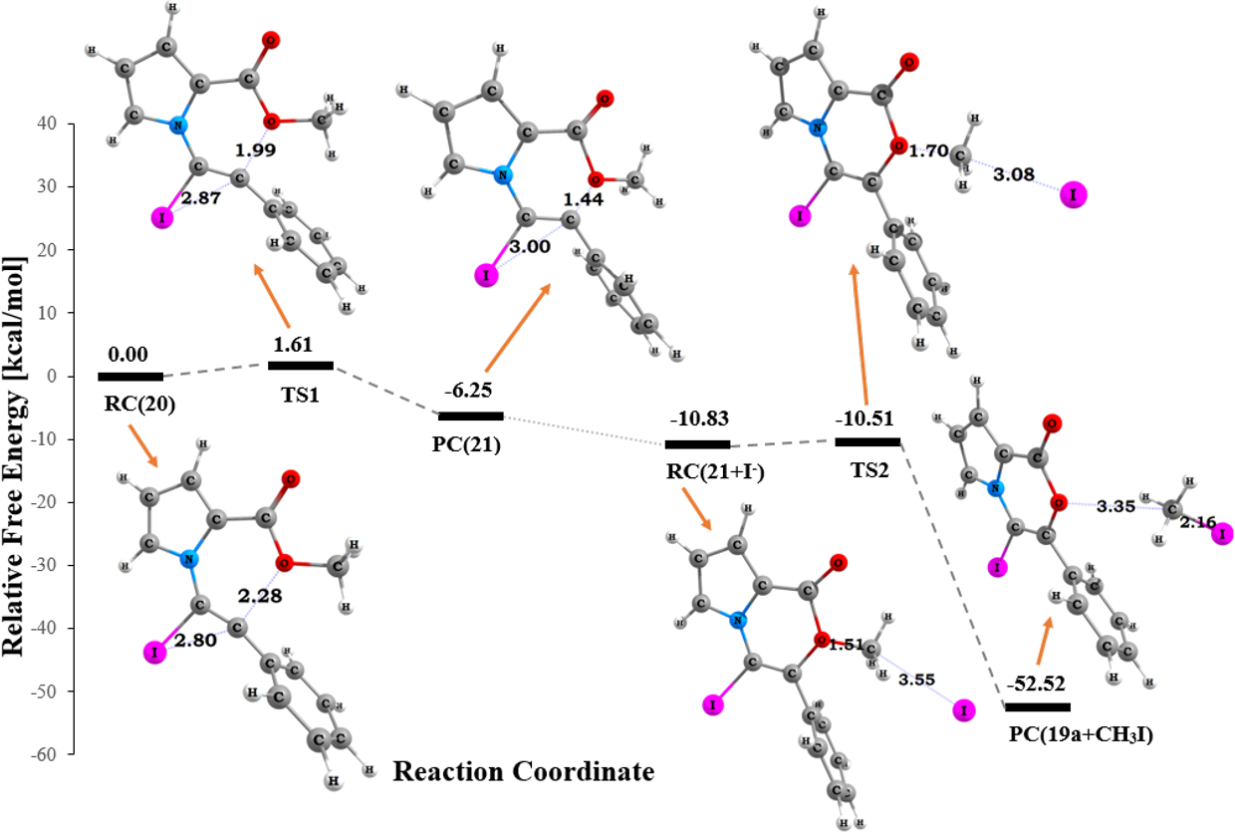
Potential energy proﬁle related to the formation of pyrrolooxazinone **19c** in the polarizable continuum model (PCM) [34–35] with the hybrid functional M06 [36] using 6-31+G(d)/LANL2DZ level in dichloromethane. Distances are given in angstroms. (Relative energies shown for **20**, **TS1**, and **21**).

According to the suggested mechanism in Scheme 6, the cyclization process occurs via the electrophilic attack of iodine to alkyne to form the complex **20**. Mulliken charges calculated at the M06/6-31+G(d)/LANL2DZ (I) level on alkyne carbon atoms C-2 and C-1 are 0.943 and −1.714, respectively. The distance between the alkyne carbon atom C-2 and the iodine atom in **20** is longer (2.80 Å) than that between the alkyne carbon atom C-1 and the iodine atom, indicating that the positive charge is more localized on the carbon atom C-2 (Figure 8). In the next step, the cyclization process occurs via the nucleophilic attack of the ester oxygen atom on the most electrophilic C-2 carbon atom to yield a 6-*endo*-dig cyclization product while iodine remains on the structure **21**. During the formation of the C2–O bond, the bond distance was shortened from 2.284 to 1.444 Å. On the other hand, the lengthening of the C2−I interaction from 2.801 to 3.001 Å and of the C1−C2 bond from 1.304 to 1.339 Å in **20** and **21** was observed (Figure 8). The activation barrier for the formation of **21** is 1.61 kcal/mol in dichloromethane and the transition state TS2 is 10.51 kcal/mol lower than the initial reactant. In the second step, the iodide anion, which already exists in the reaction media attacks the protonated methoxy group and removes the methyl group from the structure to yield the corresponding pyrrolooxazinone skeleton **19c**. The formation of **19c** is quite exergonic with a Gibbs free energy of 52.52 kcal/mol in dichloromethane. Comparison of the relative energies given in Figure 8, shows that the formation of **19c** is plausible under the given reaction conditions in dichloromethane.

## Conclusion

The synthetic strategy described in this paper shows the importance of intramolecular alkyne cyclization for the formation of interesting heterocyclic systems. We have developed an efficient method for the construction of pyrrolopyrazinone, pyrrolotriazinone, and pyrrolooxazinone moieties starting from *N-*alkyne substituted pyrrole esters. A mechanism was proposed for the formation of the title compounds. Furthermore, the methods reported here can be used for the introduction of further substituents at various positions of the target structures.

## Experimental

**General procedure for *N*-alkyne-substituted methyl 1*H*-pyrrole-2-carboxylate derivatives 7a–d.** To a solution of bromoalkyne derivatives **11** (1.1 equiv) in freshly distilled anhydrous toluene (20 mL), methyl 1*H*-pyrrole-2-carboxylate (**9**, 1.0 equiv), K_3_PO_4_ (2 equiv), CuSO_4_·5H_2_O (0.1 equiv) and 1,10-phenanthroline monohydrate (0.2 equiv) were added under N_2_ atmosphere. The reaction mixture was heated to 85 °C and stirred for 48 h. Then, the reaction mixture was cooled to room temperature and diluted with DCM (30 mL). The resulting mixture was filtered through celite and the filtrate was concentrated in vacuum. Then, the crude product was purified via column chromatography (SiO_2_, hexane) to give *N*-alkyne-substituted methyl 1*H*-pyrrole-2-carboxylate derivatives **7**.

**Methyl 1-[(4-methoxyphenyl)ethynyl]-1*H*-pyrrole-2-carboxylate (7a).** To a solution of 1-(bromoethynyl)-4-methoxybenzene (**11a**, 0.272 g, 1.290 mmol) in freshly distilled anhydrous toluene (20 mL), methyl 1*H*-pyrrole-2-carboxylate (**9**, 0.147 g, 1.170 mmol), K_3_PO_4_ (0.497 g, 2.340 mmol), CuSO_4_·5H_2_O (0.030 g, 0.120 mmol) and 1,10-phenantroline monohydrate (0.045 g, 0.230 mmol) were added and the mixture was reacted as described above. Then, the crude product was purified via column chromatography (SiO_2_, hexane/EtOAc, 20:1) to obtain **7a** (0.104 g, 35% (99% based on 35% conversion)) as a yellowish liquid. ^1^H NMR (400 MHz, CDCl_3_) δ 7.52–7.43 (m, 2H, arom.), 7.12 (dd, *J* = 2.8, 1.7 Hz, 1H, H-5), 6.98 (dd, *J* = 3.9, 1.7 Hz, 1H, H-3), 6.90–6.83 (m, 2H, arom.), 6.24 (dd, *J* = 3.9, 2.8 Hz, 1H, H-4), 3.86 (s, 3H, OCH_3_), 3.81 (s, 3H, OCH_3_); ^13^C NMR (100 MHz, CDCl_3_) δ 160.2, 159.8, 133.3, 131.4, 125.7, 118.5, 114.3, 114.1, 110.6, 80.2, 69.5, 55.4, 51.6; IR (ATR): 2951, 2257, 1719, 1605, 1543, 1515, 1458, 1438, 1416, 1361, 1334, 1265, 1246, 1179, 1168, 1100, 1069, 1027, 948, 830, 735, 598, 583; HRMS: [M + H]^+^ calcd for C_14_H_11_NO_2_, 256.09682; found, 256.09730.

**Methyl 1-[(4-nitrophenyl)ethynyl]-1*H*-pyrrole-2-carboxylate (7b).** To a solution of 1-(bromoethynyl)-4-nitrobenzene (**11b**, 0.658 g, 2.910 mmol) in freshly distilled anhydrous toluene (40 mL), methyl 1*H*-pyrrole-2-carboxylate (**9**, 0.330 g, 2.640 mmol), K_3_PO_4_ (1.121 g, 5.280 mmol), CuSO_4_·5H_2_O (0.065 g, 0.260 mmol) and 1,10-phenantroline monohydrate (0.105 g, 0.530 mmol) were added and reacted as described above. Then, the crude product was purified via column chromatography (SiO_2_, hexane/EtOAc, 20:1) to give **7b** (0.394 g, 55% (92% isolated yield based on 59% conversion)) and recrystallized as yellowish needles from chloroform, mp: 103–104 °C; ^1^H NMR (400 MHz, CDCl_3_) δ 8.21 (quasi d, *J* = 8.9 Hz, 2H, arom.), 7.67 (quasi d, *J* = 8.9 Hz, 2H, arom.), 7.16 (dd, *J* = 2.9, 1.6 Hz, 1H, H-5), 7.02 (dd, *J* = 3.8, 1.6 Hz, 1H, H-3), 6.31 (dd, *J* = 3.8, 2.9 Hz, 1H, H-4), 3.89 (s, 3H, OCH_3_); ^13^C NMR (100 MHz, CDCl_3_) δ 160.0, 147.0, 131.9, 131.2, 129.6, 126.1, 123.8, 119.3, 111.6, 86.4, 68.9, 51.8; IR (ATR): 3135, 3107, 2259, 1717, 1594, 1512, 1456, 1440, 1420, 1338, 1265, 1188, 1170, 1100, 1071, 943, 852, 754, 743, 686, 593, 517; HRMS: [M + H]^+^ calcd for C_14_H_10_N_2_O_4_, 271.07133; found, 271.07200.

**Methyl 1-(phenylethynyl)-1*H*-pyrrole-2-carboxylate (7c).** To a solution of (bromoethynyl)benzene (**11c**, 0.91 g, 5.02 mmol) in freshly distilled anhydrous toluene (20 mL), methyl 1*H*-pyrrole-2-carboxylate (**9**, 0.57 g, 4.56 mmol), K_3_PO_4_ (1.94 g, 9.12 mmol), CuSO_4_·5H_2_O (0.11 g, 0.46 mmol) and 1,10-phenanthroline monohydrate (0.18 g, 0.91 mmol) were added and the resulting mixture was reacted as described above to obtain **7c** (0.61 g, 59% (80% isolated yield based on 74% conversion)) as a yellowish liquid. ^1^H NMR (400 MHz, CDCl_3_) δ 7.61–7.47 (m, 2H, arom.), 7.39–7.29 (m, 3H, arom.), 7.18–7.10 (m, 1H, arom.), 7.00 (dd, *J* = 3.9, 1.6 Hz, 1H, H-3), 6.28–6.22 (m, 1H, arom.), 3.88 (s, 3H, OCH_3_); ^13^C NMR (100 MHz, CDCl_3_) δ 160.1, 131.6, 131.4, 128.5, 128.4, 125.8, 122.3, 118.6, 110.8, 81.4, 69.7, 51.6; IR (ATR): 2259, 1719, 1458, 1438, 1360, 1264, 1168, 1100, 1069, 751, 733, 689. HRMS: [M + H]^+^ calcd for C_14_H_11_NO_2_, 226.08626; found, 226.08840.

**Methyl 1-(hex-1-yn-1-yl)-1*H*-pyrrole-2-carboxylate (7d).** To a solution of 1-bromohex-1-yne (**11d**, 1.23 g, 7.65 mmol) in freshly distilled anhydrous toluene (30 mL), methyl 1*H*-pyrrole-2-carboxylate (**9**, 0.87 g, 6.95 mmol), K_3_PO_4_ (2.95 g, 13.9 mmol), CuSO_4_·5H_2_O (0.17 g, 0.69 mmol) and 1,10-phenantroline monohydrate (0.27 g, 1.39 mmol) were added and the resulting mixture was reacted as described above to obtain **7d** (0.83 g, 58% (92% isolated yield based on 63% conversion)) as a colorless liquid. ^1^H NMR (400 MHz, CDCl_3_) δ 7.02 (dd, *J* = 2.8, 1.7 Hz, 1H, H-5), 6.90 (dd, *J* = 3.9, 1.7 Hz, 1H, H-3), 6.16 (dd, *J* = 3.9, 2.8 Hz, 1H, H-4), 3.84 (s, 3H, OCH_3_), 2.41 (t, *J* = 7.2 Hz, 2H, CH_2_), 1.60 (q, *J* = 7.2 Hz, 2H, CH_2_), 1.48 (h, *J* = 7.2 Hz, 2H, CH_2_), 0.94 (t, *J* = 7.3 Hz, 3H, CH_3_); ^13^C NMR (100 MHz, CDCl_3_) δ 160.3, 131.8, 125.5, 118.0, 110.0, 72.6, 69.7, 51.5, 30.9, 22.1, 18.2, 13.7; IR (ATR): 2955, 2872, 2273, 1720, 1542, 1464, 1438, 1416, 1197, 1105, 926, 732, 601; HRMS: [M + H]^+^ calcd for C_12_H_15_NO_2_, 206.11756; found, 206.11960.

**Methyl 1-(2-oxo-2-phenylethyl)-1*H*-pyrrole-2-carboxylate (15).** To a solution of K_2_CO_3_ (0.246 g, 1.780 mmol) in water (15 mL) was added a solution of methyl 1-(phenylethynyl)-1*H*-pyrrole-2-carboxylate (**7c**, 0.250 g, 1.110 mmol) in MeOH (15 mL) and the reaction mixture was stirred at room temperature overnight. Then HCl (25 mL, 3 N) was added to the reaction mixture and extracted with EtOAc (3 × 25 mL) and the combined organic phase was washed with brine. The resulting mixture was dried over Na_2_SO_4_ and concentrated in vacuum. Then, the product was eluted over SiO_2_ (hexane) and concentrated in vacuum to give **7c** (0.182 g, 0.748 mmol, 96%). White needles from chloroform, mp: 109–110 °C (Lit. [15] 105–106 °C); ^1^H NMR (400 MHz, CDCl_3_) δ 8.01 (d, *J* = 7.5 Hz, 2H, arom.), 7.62 (t, *J* = 7.5 Hz, 1H, arom.), 7.51 (t, *J* = 7.5 Hz, 2H, arom.), 7.05 (dd, *J* = 3.5, 1.1 Hz, 1H, H-5), 6.85 (bs, 1H, H-3), 6.26 (dd, *J* = 3.5, 2.5 Hz, 1H, H-4), 5.76 (s, 2H, CH_2_), 3.72 (s, 3H, CH_3_); ^13^C NMR (100 MHz, CDCl_3_) δ 193.5, 161.9, 135.0, 133.7, 129.9, 129.0, 128.1, 122.3, 118.4, 108.9, 55.2, 51.2.

**General procedure for nucleophilic cyclization reactions of 7a–d with hydrazine.** To a solution of *N*-alkyne-substituted methyl 1*H*-pyrrole-2-carboxylate derivatives **7a–d** (1.0 equiv) in dry MeOH (15 mL), hydrazine monohydrate (10 equiv) was added under N_2_ atmosphere. The reaction mixture was stirred at reflux temperature for 24 h. After completion of the reaction, water (20 mL) was added. Then, MeOH was removed from the resulting mixture in vacuum. The residue was extracted with ethyl acetate (3 × 25 mL) and the organic phase was concentrated in vacuum. The resulting crude mixture was separated gradiently via column chromatography (SiO_2_, ethyl acetate/hexane, 1:4 to 1:1) and concentrated in vacuum to give the corresponding pyrrolopyrazinone and/or pyrrolotriazinone derivatives.

**2-Amino-3-phenylpyrrolo[1,2-*a*]pyrazin-1-(2*H*)-one (12c) and 4-benzylpyrrolo-[1,2-*d*][1,2,4]triazin-1(2*H*)-one (13c).** Methyl 1-(phenylethynyl)-1*H*-pyrrole-2-carboxylate (**7c**, 0.32 g, 1.42 mmol) in dry MeOH (15 mL) reacted with hydrazine monohydrate (0.71 g, 14.2 mmol) as described above to give **12c** (0.21g, 67%) as colorless cubes from ethyl acetate, mp: 167–168 °C; ^1^H NMR (400 MHz, CDCl_3_) δ 7.51–7.47 (m, 2H, arom.), 7.47–7.42 (m, 3H, arom.), 7.14 (bd, *J* = 4.0 Hz, 1H, H-8), 7.12 (dd, *J* = 2.5, 1.5 Hz, 1H, H-6), 6.94 (s, 1H, H-4), 6.61 (dd, *J* = 4.0, 2.5 Hz, 1H, H-7), 4.41 (bs, 2H, NH_2_); ^13^C NMR (100 MHz, CDCl_3_) δ 156.3, 132.3, 130.8, 129.8, 129.0, 128.3, 123.1, 118.6, 113.2, 110.4, 107.4; IR (ATR) 3297, 3097, 1664, 1630, 1430, 1376, 1347, 1294, 1184, 1009, 753, 732, 697, 627; HRMS: [M + H]^+^ calcd for C_13_H_11_N_3_O, 226.09749; found, 226.09990.

**4-Benzylpyrrolo[1,2-*d*][1,2,4]triazin-1(2*H*)-one (13c)**. (0.077 g, 24%), white needles from chloroform, mp: 199–200 °C; ^1^H NMR (400 MHz, DMSO-*d*_6_) δ 11.78 (s, 1H, N-H), 7.72–7.46 (m, 1H, H-8), 7.36 (bd, *J* = 7.3, 2H, arom.), 7.32 (bt, *J* = 7.5 Hz, 2H, arom.), 7.24 (bt, *J* = 7.5 Hz, 1H, arom.), 7.03 (bd, *J* = 3.7 Hz, 1H, H-6), 6.82–6.53 (m, 1H, H-7), 4.26 (s, 2H, CH_2_); ^13^C NMR (100 MHz, DMSO-*d*_6_) δ 154.3, 137.2, 135.2, 128.8, 128.6, 126.9, 123.5, 118.1, 114.1, 110.5, 35.9; IR (ATR); 3668, 3170, 3120, 2987, 2902, 1644, 1554, 1455, 1415, 1380, 1072, 846, 803, 695, 642, 598; HRMS: [M + H]^+^ calcd for C_13_H_11_N_3_O, 226.09749; found, 226.09760.

***N*-(1-Oxo-3-phenylpyrrolo[1,2-*a*]pyrazin-2(1*H*)-yl)acetamide (14).** 2-Amino-3-phenylpyrrolo[1,2-*a*]pyrazin-1-(2*H*)-one (**12c**, 0.200 g, 0.888 mmol) was dissolved in pyridine (5 mL) and then acetic anhydride (0.136 g, 1.332 mmol) was added at room temperature. The reaction mixture was stirred over 2 days, and then HCl (10 mL, 3 N) was added to the reaction mixture and the resulting solution was extracted with EtOAc (3 × 20 mL) and washed with brine. The organic phase was dried over Na_2_SO_4_ and concentrated in vacuum. Then, the product was eluted over SiO_2_ (ethyl acetate/hexane, 1:3) and concentrated in vacuum to give **14** (0.229 g, 0.856 mmol, 97%), snowflakes from chloroform, mp: 169.7–170.5 °C; ^1^H NMR (400 MHz, CDCl_3_) δ 9.12 (bs, 1H, NH), 7.51–7.35 (m, 5H, arom.), 7.18 (bd, *J* = 3.8 Hz, 1H, H-8), 7.13 (dd, *J* = 2.4, 1.5 Hz, 1H, H-6), 6.94 (s, 1H, H-4), 6.59 (dd, *J* = 3.8, 2.4 Hz, 1H, H-7), 1.89 (s, 3H, CH_3_); ^13^C NMR (100 MHz, CDCl_3_) δ 170.1, 155.9, 131.5, 131.4, 129.5, 129.3, 128.4, 123.0, 119.9, 113.4, 112.5, 108.0, 20.6; IR (ATR): 3367, 2971, 1473, 1373, 1341, 1315, 1281, 1081, 1040, 774, 722, 710, 632, 546; HRMS: [M + H]^+^ calcd for C_15_H_13_N_3_O_2_, 268.10805; found, 268.11080.

**4-Phenyl-2,5-dihydro-1*H*-pyrrolo[2,1-*d*][1,2,5]triazepin-1-one (16).** To a solution of methyl 1-(2-oxo-2-phenylethyl)-1*H*-pyrrole-2-carboxylate (**15**, 0.127 g, 0.522 mmol) in dry MeOH (10 mL), hydrazine monohydrate (0.261 g, 5.220 mmol) was added under N_2_ atmosphere. The reaction mixture was stirred at reflux temperature for 24 h, then water (20 mL) was added. The solvent was removed in vacuum. The residue was extracted with ethyl acetate (3 × 20 mL) and the organic phase was concentrated in vacuum. The resulting crude mixture was separated gradiently via column chromatography (SiO_2_, ethyl acetate/hexane, 1:4 to 1:1) and concentrated in vacuum to give **16** (0.032 g, 0.142 mmol, 57%) as a white solid from chloroform, mp: 224–225 °C; ^1^H NMR (400 MHz, DMSO-*d*_6_) δ 10.89 (s, 1H, NH), 7.95–7.87 (m, 2H, arom.), 7.51–7.43 (m, 3H, arom.), 7.34–7.27 (m, 1H), 6.82 (dd, *J* = 3.8, 1.7 Hz, 1H, H-9), 6.22 (dd, *J* = 3.8, 2.5 Hz, 1H, H-8), 5.28 (s, 2H, CH_2_); ^13^C NMR (100 MHz, DMSO-*d*_6_) δ 159.7, 155.1, 134.7, 130.5, 129.0, 126.7, 126.4, 125.0, 115.7, 109.7, 44.9; IR (ATR): 3206, 3069, 2929, 1634, 1604, 1537, 1409, 1372, 1347, 1307, 1179, 1074, 1023, 875, 831, 816, 749, 735, 687, 626, 577; HRMS: [M + H]^+^ calcd for C_13_H_11_N_3_O, 226.09749; found, 226.09850.

**2-Amino-3-(4-methoxyphenyl)pyrrolo[1,2-*a*]pyrazin-1(2*H*)-one (12a).** Methyl 1-[(4-methoxyphenyl)ethynyl]-1*H*-pyrrole-2-carboxylate (**7a**, 0.080 g, 0.313 mmol) was reacted with hydrazine monohydrate (0,157 g, 3.130 mmol) as described above and the resulting crude mixture was separated gradiently via column chromatography (SiO_2_, ethyl acetate/hexane, 1:10 to 1:2) and concentrated in vacuum to give **12a** (0.072 g, 0.282 mmol, 90%) as brownish needles from chloroform, mp: 168–169 °C; ^1^H NMR (400 MHz, CDCl_3_) δ 7.42 (quasi d, *J* = 8.7 Hz, 2H, arom.), 7.13 (bd, *J* = 4.0 Hz, 1H, H-8), 7.11 (dd, *J* = 2.5, 1.4 Hz, 1H, H-6), 6.96 (quasi d, *J* = 8.7 Hz, 2H, arom.), 6.91 (s, 1H, H-4), 6.60 (dd, *J* = 4.0, 2.5 Hz, 1H, H-7), 4.56 (bs, 2H, NH_2_), 3.84 (s, 3H, OCH_3_); ^13^C NMR (100 MHz, CDCl_3_) δ 160.2, 156.2, 131.2, 130.4, 124.4, 123.0, 118.4, 113.7, 113.1, 110.2, 107.2, 55.4; IR (ATR): 3314, 3107, 2920, 1670, 1605, 1510, 1473, 1373, 1341, 1242, 1176, 1021, 965, 830, 800, 736, 634, 595; HRMS: [M + H]^+^ calcd for C_13_H_8_INO_2_, 256.10805; found, 256.10860.

**4-(4-Nitrobenzyl)pyrrolo[1,2-*d*][1,2,4]triazin-1(2*H*)-one (13b).** Methyl 1-[(4-nitrophenyl)ethynyl]-1*H*-pyrrole-2-carboxylate (**7b**, 0.30 g, 1.46 mmol) in dry MeOH (15 mL) was reacted with hydrazine monohydrate (0.205 g, 0.758 mmol) as described above to give **13b** (0.190 g, 0.703 mmol, 97%), yellowish pellets from chloroform, mp: 237–238 °C; ^1^H NMR (400 MHz, DMSO-*d*_6_) δ 10.92 (s, 1H, NH), 7.42–7.25 (m, 2H, arom.), 6.86–6.71 (m, 3H, arom. and H-6), 6.19 (dd, *J* = 3.7, 1.2 Hz, 1H, H-8), 5.88 (dd, *J* = 3.7, 3.1 Hz, 1H, H-7), 3.59 (s, 2H, CH_2_); ^13^C NMR (100 MHz, DMSO-*d*_6_) δ 154.4, 146.7, 143.2, 136.6, 130.6, 123.6, 123.6, 118.1, 114.4, 110.8, 35.5; IR (ATR): 3144, 3110, 2259, 1717, 1594, 1512, 1457, 1440, 1420, 1339, 1265, 1220, 1170, 1100, 1071, 943, 852, 754, 743, 686, 593; HRMS: [M + H]^+^ calcd for C_13_H_10_N_4_O_3_, 271.08257; found, 271.08380.

**2-Amino-3-butylpyrrolo[1,2-*a*]pyrazin-1(2*H*)-one (12d).** Methyl 1-(hex-1-yn-1-yl)-1*H*-pyrrole-2-carboxylate (**7d**, 0.30 g, 1.46 mmol) in dry MeOH (15 mL) was reacted with hydrazine monohydrate (0.73 g, 14.6 mmol) as described above to obtain 2-amino-3-butylpyrrolo[1,2-*a*]pyrazin-1(2*H*)-one (**12d**, 0.26 g, 87%), colorless needles from chloroform, mp: 119–120 °C; ^1^H NMR (400 MHz, CDCl_3_) δ 7.09–6.93 (m, 2H, arom.), 6.71 (s, 1H, H-4), 6.52–6.46 (m, 1H, arom.), 4.54 (s, 2H, NH_2_), 2.59 (t, *J* = 7.4 Hz, 2H, CH_2_), 1.58 (qui, *J* = 7.4 Hz, 2H, CH_2_), 1.40 (h, *J* = 7.4 Hz, 2H, CH_2_), 0.93 (t, *J* = 7.4 Hz, 3H, CH_3_); ^13^C NMR (100 MHz, CDCl_3_) δ 156.8, 130.7, 122.9, 117.7, 112.3, 109.5, 104.8, 30.7, 29.5, 22.3, 13.9; IR (ATR): 3287, 3204, 3111, 2950, 2934, 2867, 1670, 1618, 1596, 1414, 1371, 1346, 1232, 1071, 971, 878, 742, 690, 639; HRMS: [M + H]^+^ calcd for C_11_H_15_N_3_O, 206.12879; found, 206.13010.

**General procedure for electrophilic cyclization reactions of 7 with iodine**. To a solution of *N*-alkyne-substituted methyl 1*H*-pyrrole-2-carboxylate derivatives **7** in dichloromethane (10 mL), I_2_ (1.0 equiv) was added. The reaction mixture was stirred at room temperature for 2 h. After completion of the reaction, the mixture was concentrated in vacuum. Then, the crude product was purified via column chromatography (SiO_2_, ethyl acetate/hexane, 1:5) and concentrated in vacuum to obtain the corresponding iodine-substituted pyrrolo-oxazinone derivatives **19**.

**4-Iodo-3-phenyl-1*H*-pyrrolo[2,1-*c*][1,4]oxazin-1-one (19c).** To a solution of methyl 1-(phenylethynyl)-1*H*-pyrrole-2-carboxylate (**7c**, 50.0 mg, 0.22 mmol) in dichloromethane (10 mL), I_2_ (56.3 mg, 0.22 mmol) was added and treated as described above to give **19c** (58.9 mg, 79%) as colorless cubes from chloroform, mp: 176–178 °C; ^1^H NMR (400 MHz, CDCl_3_) δ 7.69–7.59 (m, 2H, arom.), 7.52–7.47 (m, 2H, arom.), 7.47–7.42 (m, 3H, arom.), 6.61 (dd, *J* = 4.0, 2.8 Hz, 1H, H-7); ^13^C NMR (100 MHz, CDCl_3_) δ 153.3, 142.6, 131.8, 129.1, 129.0, 127.2, 125.8, 116.8, 116.4, 111.7, 68.3; IR (ATR) 3664, 2969, 2918, 1708, 1450, 1371, 1332, 1089, 1055, 766, 729, 696, 685; HRMS: [M + H]^+^ calcd for C_13_H_8_INO_2_, 337.96791; found, 337.97070.

**4-Iodo-3-(4-nitrophenyl)-*1H-*pyrrolo[2,1-*c*][1,4]oxazin-1-one (19b).** To a solution of methyl 1-[(4-nitrophenyl)ethynyl]-1*H*-pyrrole-2-carboxylate (**7b**, 0.108 g, 0.400 mmol) in DCM (20 mL), I_2_ (0.101 g, 0.400 mmol) was added and the reaction mixture was stirred for 5 h. The crude product was purified as described above to give **19b** (0.116 g, 76%) as yellowish needle from chloroform, mp: 171–172 °C; ^1^H NMR (400 MHz, CDCl_3_) δ 8.32 (quasi d, *J* = 8.9 Hz, 2H, arom.), 7.90 (quasi d, *J* = 8.9 Hz, 2H, arom.), 7.58–7.48 (m, 2H, H-6 and H-8), 6.67 (dd, *J* = 3.9, 2.9 Hz, 1H, H-7); ^13^C NMR (100 MHz, CDCl_3_) δ 153.5, 148.4, 141.4, 138.9, 131.2, 127.2, 123.5, 118.6, 117.3, 113.3, 70.8; IR (ATR): 3144, 1731, 1594, 1447, 1406, 1332, 1249, 1182, 1107, 1074, 1033, 1011, 939, 855, 738, 707, 694, 676, 598; HRMS: [M + H]^+^ calcd for C_13_H_7_IN_2_O_4_, 382.95233; found, 382.95660.

**4-Iodo-3-butyl-1*H*-pyrrolo[2,1-*c*][1,4]oxazin-1-one (19d).** To a solution of methyl 1-(hex-1-yn-1-yl)-1*H*-pyrrole-2-carboxylate (**7d**, 0.120 g, 0.585 mmol) in CHCl_3_ (15 mL), I_2_ (0.15 g, 0.59 mmol) was added and the reaction mixture was stirred for 5 h. The crude product was purified as described above to give **19d** (58.9 mg, 79%) as colorless cubes from chloroform, mp: 176–178 °C; ^1^H NMR (400 MHz, CDCl_3_) δ 7.39 (dd, *J* = 4.1, 1.6 Hz, 1H, H-8), 7.33 (dd, *J* = 2.7, 1.6 Hz, 1H, H-6), 6.52 (dd, *J* = 4.1, 2.7 Hz, 1H, H-7), 2.70 (t, *J* = 7.5 Hz, 2H, CH_2_), 1.66 (qui, *J* = 7.5 Hz, 2H, CH_2_), 1.39 (h, *J* = 7.5 Hz, 2H, CH_2_), 0.93 (t, *J* = 7.5 Hz, 3H, CH_3_); ^13^C NMR (100 MHz, CDCl_3_) δ 154.7, 145.7, 125.9, 117.6, 117.4, 112.3, 68.6, 34.0, 29.3, 22.1, 13.9; IR (ATR) 2956, 2928, 2869, 1635, 1534, 1455, 1402, 1339, 1229, 1023, 1084, 1027, 997, 896, 620, 596; HRMS: [M + H]^+^ calcd for C_11_H_12_INO_2_, 317.99855; found, 317.99880.

## Supporting Information

File 1 NMR spectra, X-ray crystallographic data, and Cartesian Coordinates for the optimized structures.
