# Supplementary material for: Nucleophilic and electrophilic cyclization of N-alkyne-substituted pyrrole derivatives: Synthesis of pyrrolopyrazinone, pyrrolotriazinone, and pyrrolooxazinone moieties
Source: Beilstein J Org Chem. 2017 May 4;13:825–34. doi: 10.3762/bjoc.13.83 (PMC5433174; doi:10.3762/bjoc.13.83)
Supplement: File 1 — NMR spectra, X-ray crystallographic data, and Cartesian Coordinates for the optimized structures. [file Beilstein_J_Org_Chem-13-825-s001.pdf]

Supporting Information

for

Nucleophilic and electrophilic cyclization of *N*-alkyne-substituted pyrrole derivatives: Synthesis of pyrrolopyrazinone, pyrrolotriazinone, and pyrrolo-oxazinone moieties

Işıl Yenice[1], Sinan Basceken[1,2], Metin Balci*[1]

Address: [1]Department of Chemistry, Middle East Technical University, 06800 Ankara, Turkey and [2]Department of Chemistry, Hitit University, 19030 Corum, Turkey

Email: Metin Balci* - mbalci@metu.edu.tr

*Corresponding author

NMR spectra, X-ray crystallographic data, and Cartesian Coordinates for the optimized structures

Table of contents

1.	General remarks	S3
2.	2,2,2-Trichloro-1-(1*H*-pyrrol-2-yl)ethanone (**8**)	S3
3.	Methyl 1*H*-pyrrole-2-carboxylate (**9**)	S3
4.	[(4-Methoxyphenyl)ethynyl]trimethylsilane	S4
5.	[(4-Nitrophenyl)ethynyl]trimethylsilane	S4
6.	1-Ethynyl-4-methoxybenzene (**10a**)	S4
7.	General procedure for synthesis of bromoalkyne derivatives **11**	S5
8.	1-(Bromoethynyl)-4-methoxybenzene (**11a**)	S5
9.	1-(Bromoethynyl)-4-nitrobenzene (**11b**)	S5
10.	(Bromoethynyl)benzene (**11c**)	S5

11. 1-Bromohex-1-yne (**11d**) S6
12. ^{1}H and ^{13}C NMR spectra of 2,2,2-trichloro-1-(1H-pyrrol-2-yl)ethanone (**8**) S7
13. ^{1}H and ^{13}C NMR spectra of methyl 1H-pyrrole-2-carboxylate (**9**) S8
14. ^{1}H and ^{13}C NMR spectra of [(4-methoxyphenyl)ethynyl]trimethylsilane S9
15. ^{1}H and ^{13}C NMR spectra of [(4-nitrophenyl)ethynyl]trimethylsilane S10
16. ^{1}H and ^{13}C NMR spectra of 1-ethynyl-4-methoxybenzene (**10a**) S11
17. ^{1}H and ^{13}C NMR spectra of 1-(bromoethynyl)-4-methoxybenzene (**11a**) S12
18. ^{1}H and ^{13}C NMR spectra of 1-(bromoethynyl)-4-nitrobenzene (**11b**) S13
19. ^{1}H and ^{13}C NMR spectra of (bromoethynyl)benzene (**11c**) S14
20. ^{1}H and ^{13}C NMR spectra of 1-bromohex-1-yne (**11d**) S15
21. ^{1}H and ^{13}C NMR spectra of **7a** S16
22. ^{1}H and ^{13}C NMR spectra of **7b** S17
23. ^{1}H and ^{13}C NMR spectra of **7c** S18
24. ^{1}H and ^{13}C NMR spectra of **7d** S19
25. ^{1}H and ^{13}C NMR spectra of **15** S20
26. ^{1}H and ^{13}C NMR spectra of **12c** S21
    HSQC spectra of **12c** S22
    HMBC spectra of **12c** S23
27. ^{1}H and ^{13}C NMR spectra of **13c** S24
    HSQC spectra of **13c** S25
    HMBC spectra of **13c** S26
28. ^{1}H and ^{13}C NMR spectra of **14** S27
29. ^{1}H and ^{13}C NMR spectra of **16** S28
    HSQC spectra of **16** S29
    HMBC spectra of **16** S30
30. ^{1}H and ^{13}C NMR spectra of **12a** S31
31. ^{1}H and ^{13}C NMR spectra of **13b** S32
32. ^{1}H and ^{13}C NMR spectra of **12d** S33
33. ^{1}H and ^{13}C NMR spectra of **19c** S34
34. ^{1}H and ^{13}C NMR spectra of **19b** S35
35. ^{1}H and ^{13}C NMR spectra of **19d** S36
36. Theoretical calculations S37
37. X-ray data for **12c** and **19c** S38–S44

General remarks

All reagents were used as purchased from commercial suppliers without further purification. Proton nuclear magnetic resonance spectra (^{1}H NMR) were recorded on a 400 MHz instrument, and chemical shifts are reported in parts per million (ppm) downfield from TMS, using residual $CDCl_3$ as an internal standard. The ^{13}C NMR spectra were recorded on a 100 MHz instrument and are reported in ppm using solvent as an internal standard ($CDCl_3$). Column chromatography was performed on silica gel (60 mesh). TLC was carried out on 0.2 mm silica gel 60 F254 analytical aluminum plates. High-resolution mass spectra were recorded by LC–MS–TOF electrospray ionization. Chemicals and all solvents were commercially available and used without further purification. Infrared (IR) spectra were recorded in the range 4000–600 cm^{-1} via diamond ATR. Melting points were measured using a melting point apparatus and were uncorrected. Evaporation of solvents was performed at reduced pressure, using a rotary vacuum evaporator.

2,2,2-Trichloro-1-(1*H*-pyrrol-2-yl)ethanone (8) [1,2]. To a solution of trichloroacetyl chloride (14.3 g, 78.6 mmol) in dry diethyl ether (20 mL), pyrrole (4.80 g, 71.6 mmol) was added over 1 h. The reaction mixture was stirred for an additional hour at room temperature, and then the reaction mixture was neutralized with an aqueous potassium carbonate solution (6.10 g, 44.1 mmol) in 20 mL water. Then the extraction was performed with diethyl ether (3 × 25 mL) and the organic phase was dried over Na_2SO_4. After the filtration and evaporation of the solvent, the crude product was purified via column chromatography (SiO_2, ethyl acetate/n-hexane, 1:7) and concentrated in vacuum to give **8** (13.52 g, 89%) and recrystallized as colorless needles from ethyl acetate/n-hexane. Mp. 75–76 °C. (Lit. [1] 73.5–74 °C). ^{1}H-NMR (400 MHz, $CDCl_3$) δ 9.60 (bs, 1H, NH), 7.39 (ddd, J = 3.7, 2.4, 1.1 Hz, 1H, H-4), 7.19–7.16 (m, 1H, arom.), 6.41–6.37 (m, 1H, arom.); ^{13}C NMR (100 MHz, $CDCl_3$) δ 173.4, 127.3, 123.1, 121.3, 112.0, 95.1.

Methyl 1*H*-pyrrole-2-carboxylate (9) [1,3]. In dry methanol (40 mL), sodium (0.14 g, 6.20 mmol) was dissolved and trichloroacetyl-1*H*-pyrrole (**8**) (9.440 g, 44.43 mmol) was added in small quantities over 30 min. Then the reaction mixture was stirred for additional 2 hours at room temperature, and then the solvent was removed and resulting crystals were dissolved in diethyl ether (50 mL). Ether solution was washed with HCl (4 mL, 3 N) and then $NaHCO_3$ (10 mL) solution. Then the organic phase was dried over Na_2SO_4. After the filtration and evaporation of the solvent, the crude product was purified via column chromatography (SiO_2, ethyl acetate/hexane, 2:3) and concentrated in vacuum to obtain methyl 1*H*-pyrrole-2-

carboxylate **9** (4.83 g, 87%) which was crystallized from ethyl acetate/hexane as colorless pellets, mp: 72–73 °C. (Lit. [3] 69–70 °C); ^{1}H NMR (400 MHz, $CDCl_3$) δ 9.74 (bs, 1H, N-H), 6.97 (ddd, *J* = 4.1, 2.7, 1.5 Hz, 1H, H-4), 6.95–6.91 (m, 1H, arom.), 6.31–6.21 (m, 1H, arom.), 3.86 (s, 3H, $-OCH_3$); ^{13}C NMR (100 MHz, $CDCl_3$) δ 162.0, 123.3, 122.6, 115.5, 110.4, 51.5.

[(4-Methoxyphenyl)ethynyl]trimethylsilane [4-6]. To a solution of $PdCl_2$ (30.00 mg, 0.171 mmol), PPh_3 (90.00 mg, 0.342 mmol), CuI (16.00 mg, 0.085 mmol), in triethylamine (30 mL) was added 4-iodoanisole (1.00 g, 4.27 mmol) under N_2 atmosphere, the mixture was stirred for 10 minutes and trimethylsilylacetylene (0.839 g, 8.540 mmol) was added. Then, the reaction mixture was stirred at 80 °C for 3 hours. After completion of the reaction, the reaction mixture was filtered and the filtrate was concentrated in vacuum. The resulting crude mixture was eluted through a SiO_2 column (ethylacetate/hexane, 1:10) and concentrated in vacuum to give [(4-methoxyphenyl)ethynyl]trimethylsilane (0.768 g, 88%) as a yellowish liquid. ^{1}H NMR (400 MHz, $CDCl_3$) δ 7.41 (quasi d, *J* = 8.9 Hz, 2H, arom.), 6.82 (quasi d, *J* = 8.9 Hz, 2H, arom.), 3.80 (s, 3H, $-CH_3$), 0.24 (s, 9H, TMS); ^{13}C NMR (100 MHz, $CDCl_3$) δ 159.9, 133.6, 115.4, 113.9, 105.3, 92.6, 55.4, 0.2.

[(4-Nitrophenyl)ethynyl]trimethylsilane [4-6]. To a solution of $PdCl_2$ (29.00 mg, 0.163 mmol), PPh_3 (85.00 mg, 0.326 mmol), CuI (15.00 mg, 0.082 mmol), in triethylamine (30 mL) was added 1-iodo-4-nitrobenzene (2.036 g, 8.176 mmol) under N_2 atmosphere, the mixture was stirred for 10 minutes and then trimethylsilylacetylene (0.964 g, 9.815 mmol) was added. The reaction mixture was stirred at 80 °C for 4 h. After completion of the reaction, the reaction mixture was filtered and the filtrate was concentrated in vacuum. The resulting crude mixture was eluted through a SiO_2 column (ethylacetate/hexane, 1:10) and concentrated in vacuum to obtain [(4-nitrophenyl)ethynyl]-trimethylsilane (1.614 g, 90%) and recrystallized as yellowish solid from chloroform, mp: 98–99 °C (Lit. [6] 96–97 °C); ^{1}H NMR (400 MHz, $CDCl_3$) δ 8.17 (quasi d, *J* = 8.9 Hz, 2H, arom.), 7.59 (quasi d, *J* = 8.9 Hz, arom.), 0.27 (s, 9H, TMS); ^{13}C NMR (100 MHz, $CDCl_3$) δ 147.3, 132.8, 130.1, 123.6, 102.8, 100.8, 0.2.

1-Ethynyl-4-methoxybenzene (10a) [4-6]. To a solution of MeOH/$CHCl_3$ (30 mL, 1:1) was added ((4-methoxyphenyl)ethynyl)trimethylsilane (0.768 g, 3.760 mmol) and then K_2CO_3 (0.624 g, 4.510 mmol). The reaction mixture was stirred at room temperature for an hour. After completion of the reaction, the reaction mixture was concentrated in vacuum. The residue was diluted with EtOAc (50 mL) and washed with HCl (4 N, 40 mL) then with brine (3 × 40 mL). The resulting crude mixture was eluted through a SiO_2 column (hexane) and

concentrated in vacuum to obtain **10a** (0.487 g, 98%) as a colorless oil. ^{1}H NMR (400 MHz, $CDCl_3$) δ 7.44 (quasi d, *J* = 8.6 Hz, 2H, arom.), 6.85 (quasi d, *J* = 8.6 Hz, 2H, arom.), 3.81 (s, 3H, OCH_3), 3.01 (s, 1H, C≡CH); ^{13}C NMR (100 MHz, $CDCl_3$) δ 160.1, 133.7, 114.3, 114.1, 83.8, 75.9, 55.4.

General procedure for synthesis of bromoalkyne derivatives (11). To a solution of terminal alkyne derivatives **10** (1.0 equiv) in acetone (30 mL) were added NBS (1.1 equiv) and $AgNO_3$ (0.1 equiv) and the resulting mixture was stirred at room temperature for 4 hours. After completion of the reaction, the reaction mixture was filtered and the filtrate was concentrated in vacuum. Then, the resulting crude mixture was added to distilled water (20 mL) and extracted with diethyl ether (3 × 25 mL) and washed with brine. Then the organic phase was dried over Na_2SO_4 and concentrated in vacuum. The crude product was purified via column chromatography (SiO_2, hexane) and concentrated in vacuum to obtain bromoalkyne derivatives**.**

1-(Bromoethynyl)-4-methoxybenzene (11a) [7,8]. A solution of 1-ethynyl-4-methoxybenzene (**11a**) (0.51 g, 3.86 mmol) in acetone (30 mL) were treated with NBS (0.76 g, 4.25 mmol) in the presence of $AgNO_3$ (0.065 g 0.386 mmol) as described above to obtain **11a** (0.72 g, 89%) as a yellowish oil. ^{1}H NMR (400 MHz, $CDCl_3$) δ 7.62–7.47 (m, 2H, arom.), 6.76–6.60 (m, 2H, arom.), 3.78 (s, 3H, OCH_3); ^{13}C NMR (100 MHz, $CDCl_3$) δ 159.6, 138.3, 133.6, 116.5, 82.8, 55.4, 29.8.

1-(Bromoethynyl)-4-nitrobenzene (11b) [7,8]. To a solution of [(4-nitrophenyl)ethynyl]trimethylsilane (0.654 g, 2.980 mmol) in acetone (30 mL) were added $AgNO_3$ (0.151 g 0.890 mmol) and NBS (0.637 g, 3.580 mmol), then the resulting mixture was stirred at room temperature in dark for 2 hours. After completion of the reaction, the reaction mixture was concentrated in vacuum. Then, the resulting crude mixture was purified over silica gel column chromatography eluting with CH_2Cl_2/hexane, 2:3). Concentration of the solvent in vacuum gave **11b** (0.658 g, 98%) as a light yellowish solid, mp: 165–166 °C. ^{1}H NMR (400 MHz, $CDCl_3$) δ 8.18 (quasi d, *J* = 8.8 Hz, 2H, arom.), 7.59 (quasi d, *J* = 8.8 Hz, 2H, arom.); ^{13}C NMR (100 MHz, $CDCl_3$) δ 147.5, 133.0, 129.6, 123.7, 78.6, 56.5.

(Bromoethynyl)benzene (11c) [7,8]. A solution of phenylacetylene (**10c**) (0.58 g, 5.68 mmol) in acetone (30 mL) were treated with NBS (1.11 g, 6.25 mmol) in the presence of $AgNO_3$ (0.11 g 0.57 mmol) as described above to obtain **11c** (1.01 g, 98%) as a yellowish liquid. ^{1}H NMR (400 MHz, $CDCl_3$) δ 7.50–7.41 (m, 2H, arom.), 7.37–7.28 (m, 3H, arom.); ^{13}C NMR (100 MHz, $CDCl_3$) δ 132.1, 128.8, 128.5, 122.8, 80.2, 49.9.

1-Bromohex-1-yne (11d) [7,8]. A solution of 1-hexyne (3.50 g, 42.6 mmol) in acetone (150 mL) were added $AgNO_3$ (0.84 g 4.30 mmol) and stirred for 5 minutes. To the resulting mixture was added NBS (9.09 g, 51.1 mmol) and stirred at room temperature for 90 min and **11d** (6.38 g, 39.6 g, 93%) was obtained as a colorless liquid as described above. ^{1}H-NMR (400 MHz, $CDCl_3$) δ 2.20 (t, *J* = 7.1 Hz, 2H, H-3), 1.54–1.44 (m, 2H, H-4), 1.44–1.34 (m, 2H, H-5), 0.91 (t, *J* = 7.1 Hz, 3H, H-6); ^{13}C NMR (100 MHz, $CDCl_3$) δ 80.5, 37.5, 30.5, 22.0, 19.5, 13.6.

^{1}H and ^{13}C NMR spectra

2,2,2-Trichloro-1-(1*H*-pyrrol-2-yl)ethanone (**8**).

^{1}H-NMR: (400 MHz, $CDCl_3$)

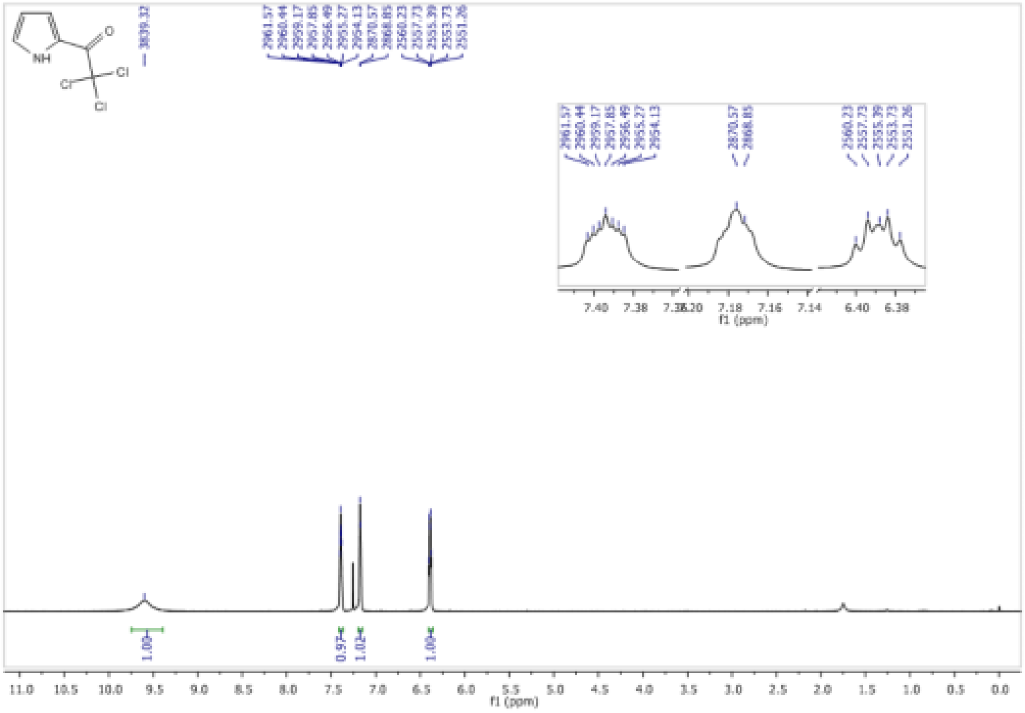

^{13}C-NMR: (100 MHz, $CDCl_3$)

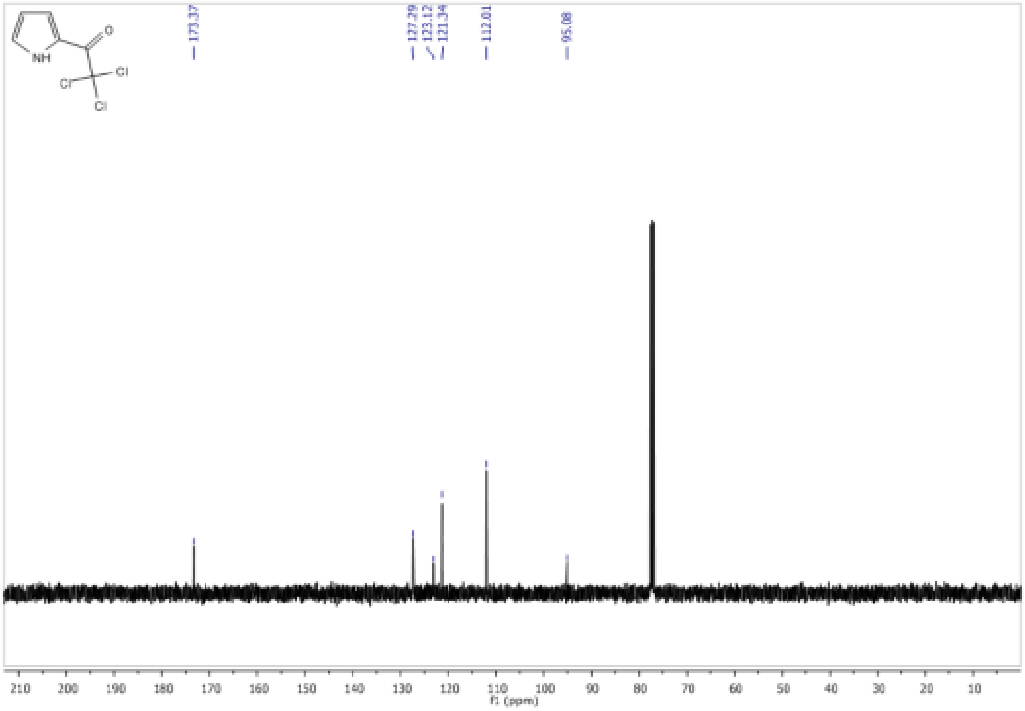

Methyl 1*H*-pyrrole-2-carboxylate (**9**).

^{1}H-NMR: (400 MHz, $CDCl_3$)

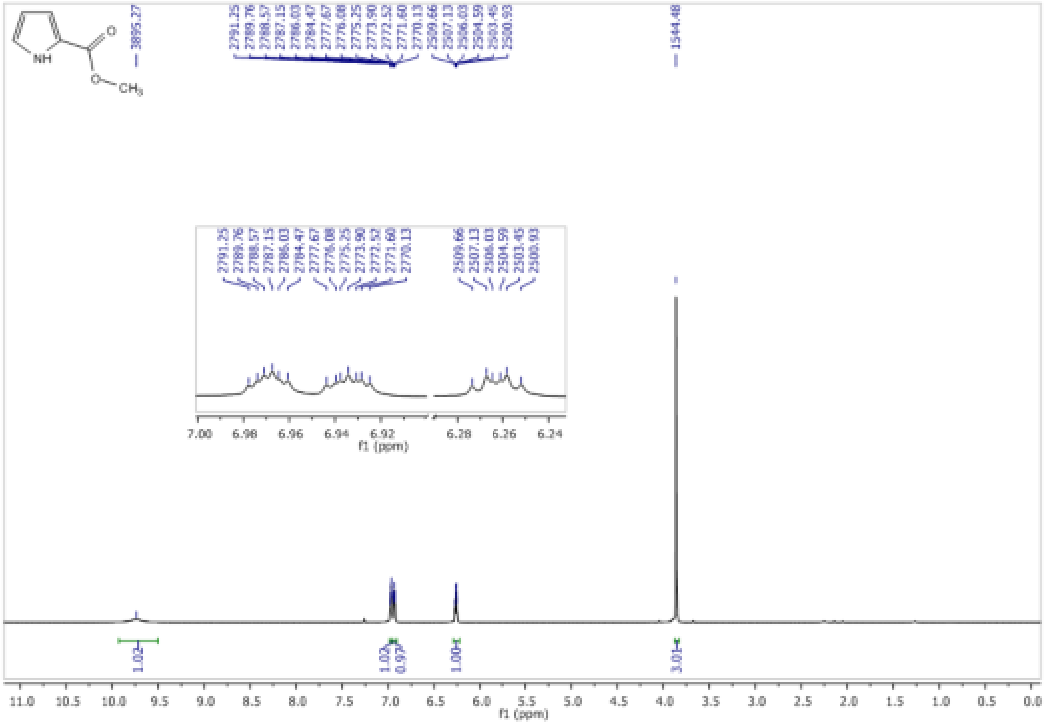

^{13}C-NMR: (100 MHz, $CDCl_3$)

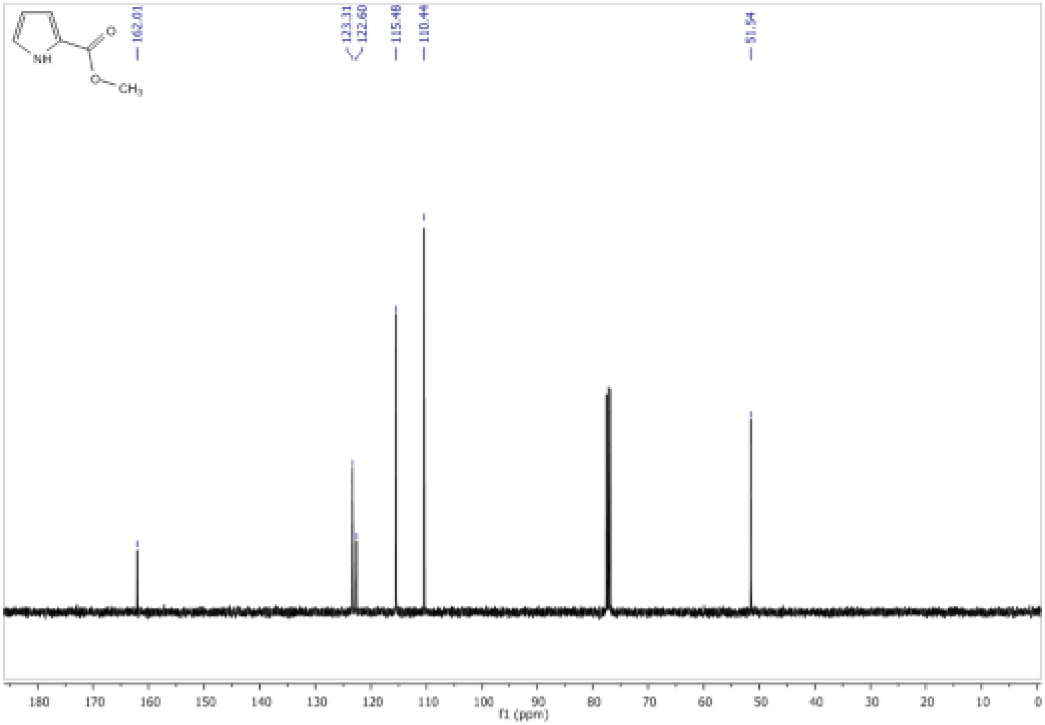

[(4-Methoxyphenyl)ethynyl]trimethylsilane.

^{1}H-NMR: (400 MHz, $CDCl_3$)

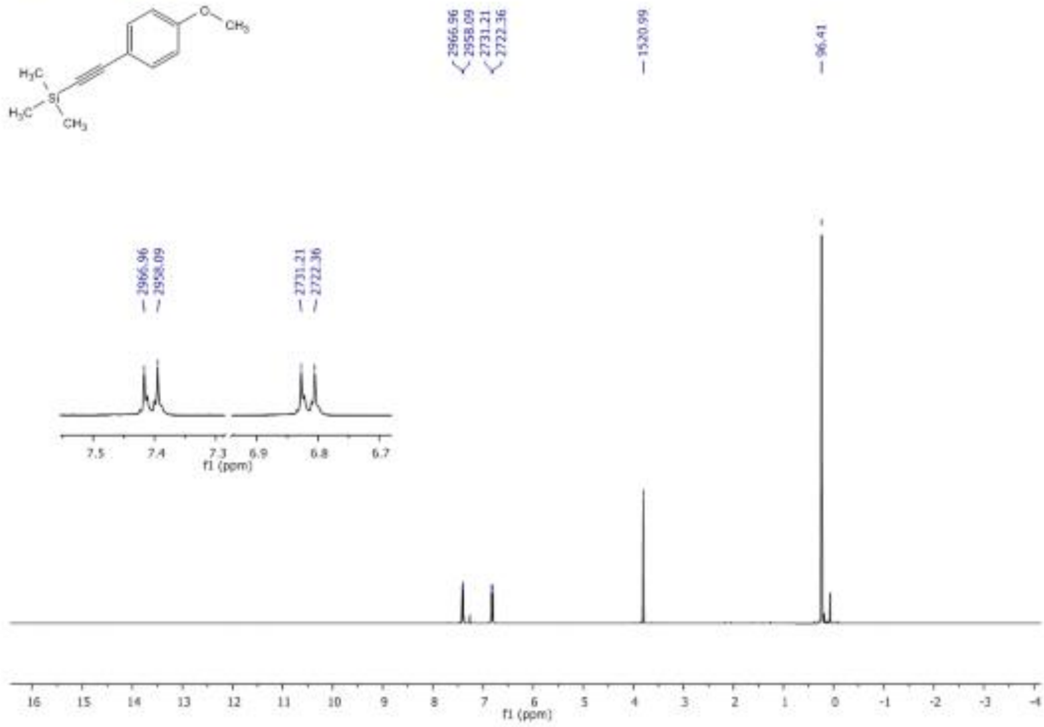

^{13}C-NMR: (100 MHz, $CDCl_3$)

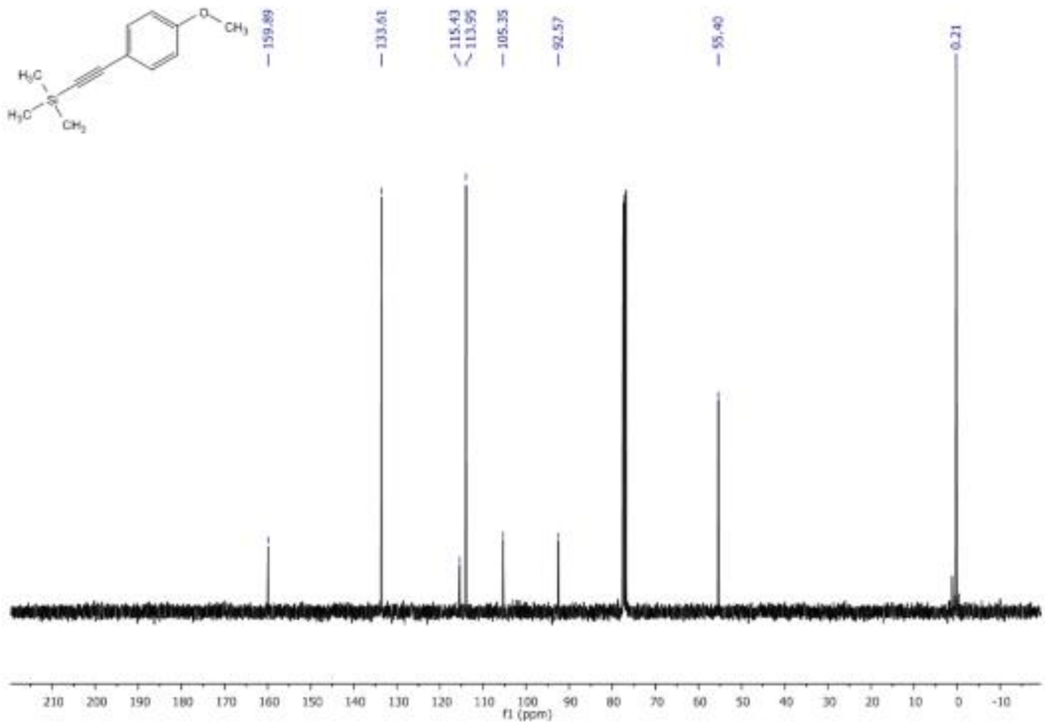

[(4-Nitrophenyl)ethynyl]trimethylsilane.

^{1}H-NMR: (400 MHz, $CDCl_3$)

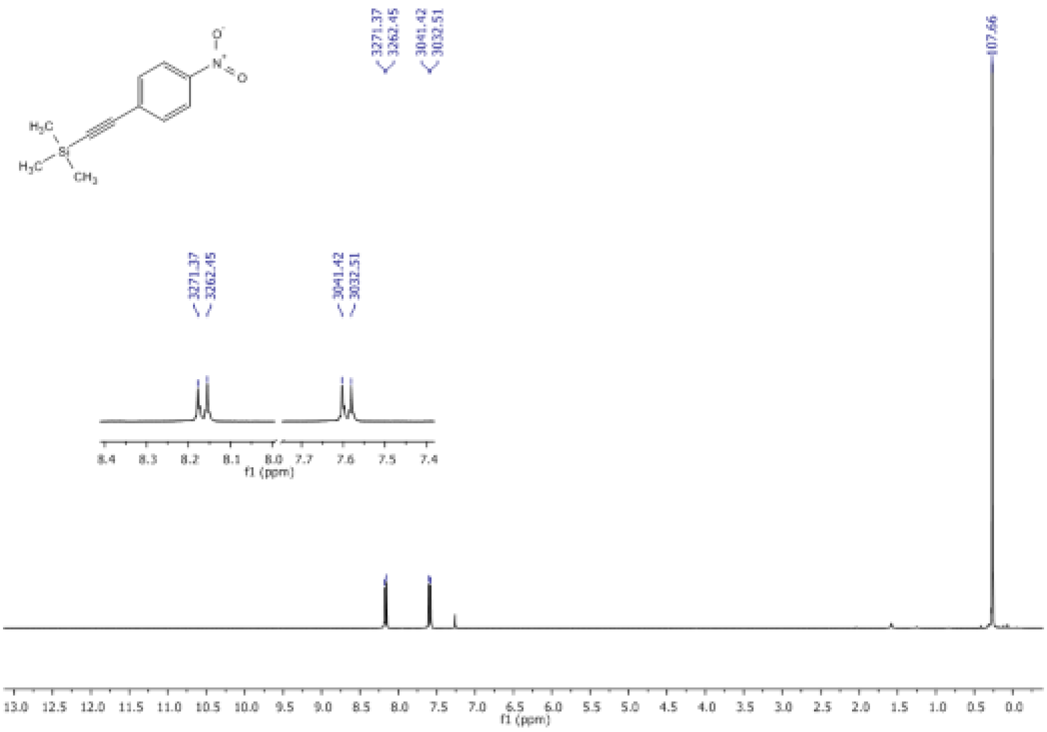

^{13}C-NMR: (100 MHz, $CDCl_3$)

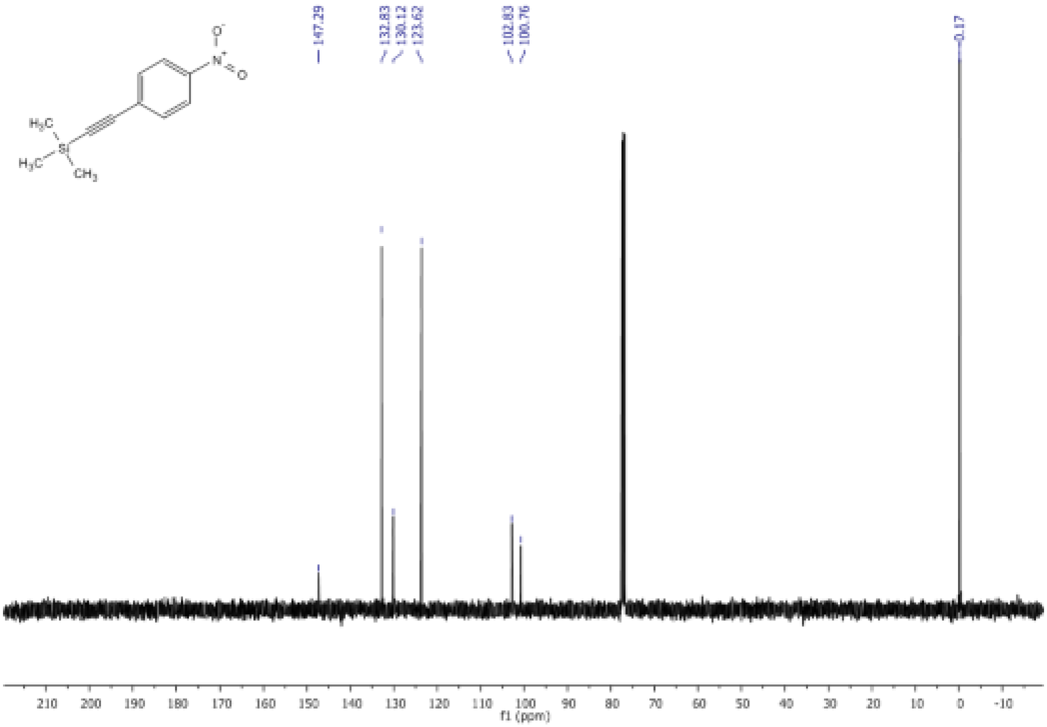

1-Ethynyl-4-methoxybenzene (**10a**).

^{1}H-NMR: (400 MHz, $CDCl_3$)

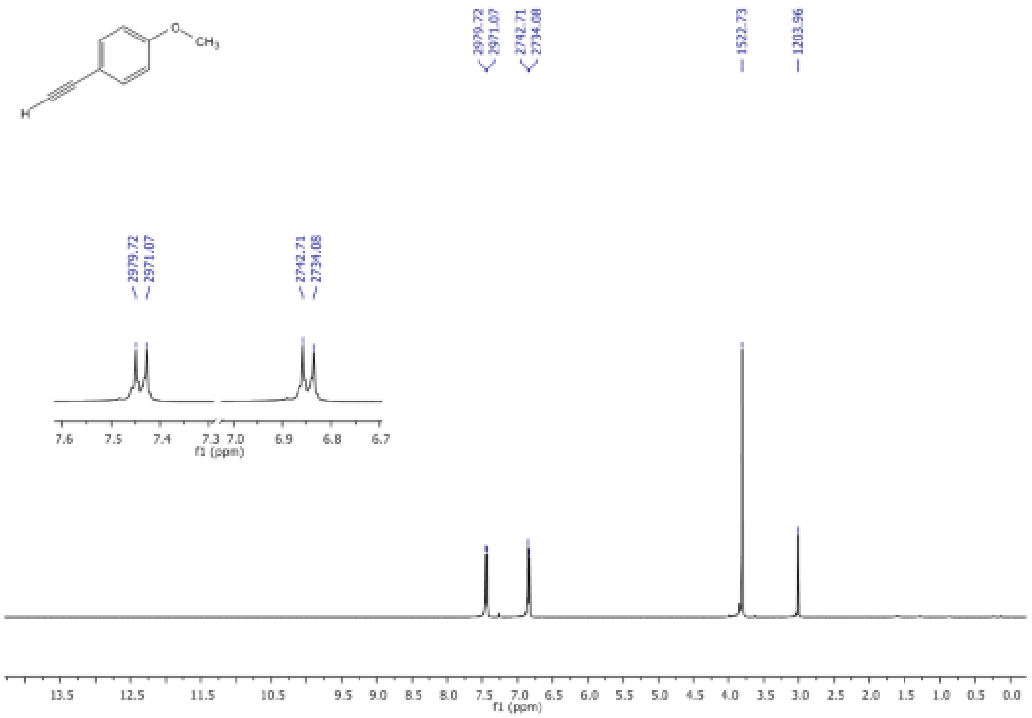

^{13}C-NMR: (100 MHz, $CDCl_3$)

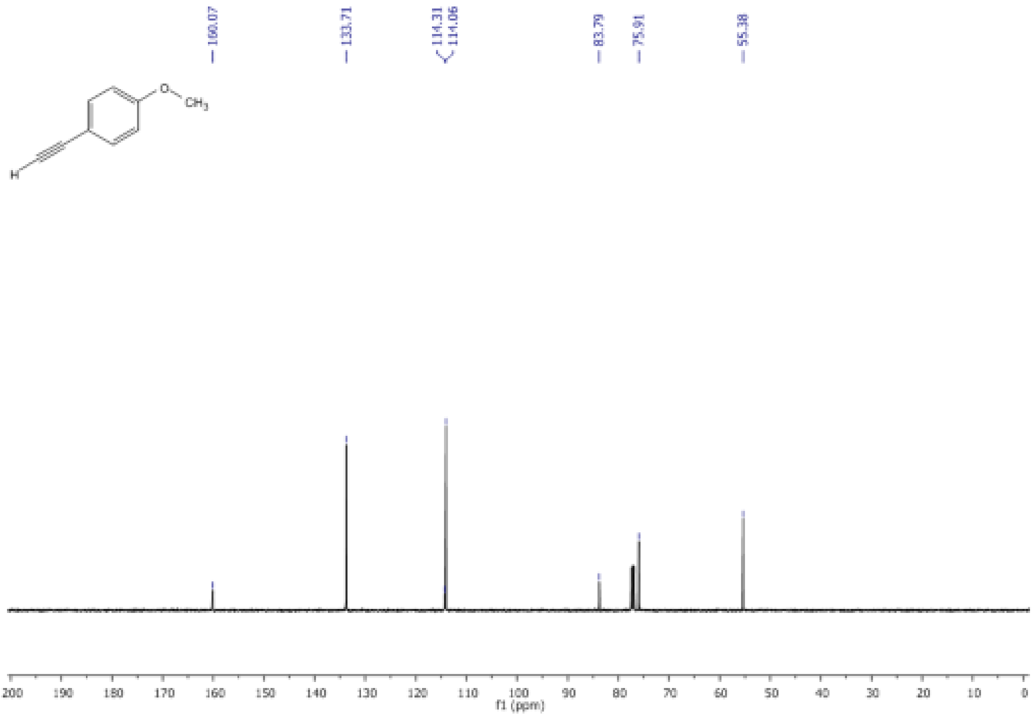

1-(Bromoethynyl)-4-methoxybenzene (**11a**).

^{1}H-NMR: (400 MHz, $CDCl_3$)

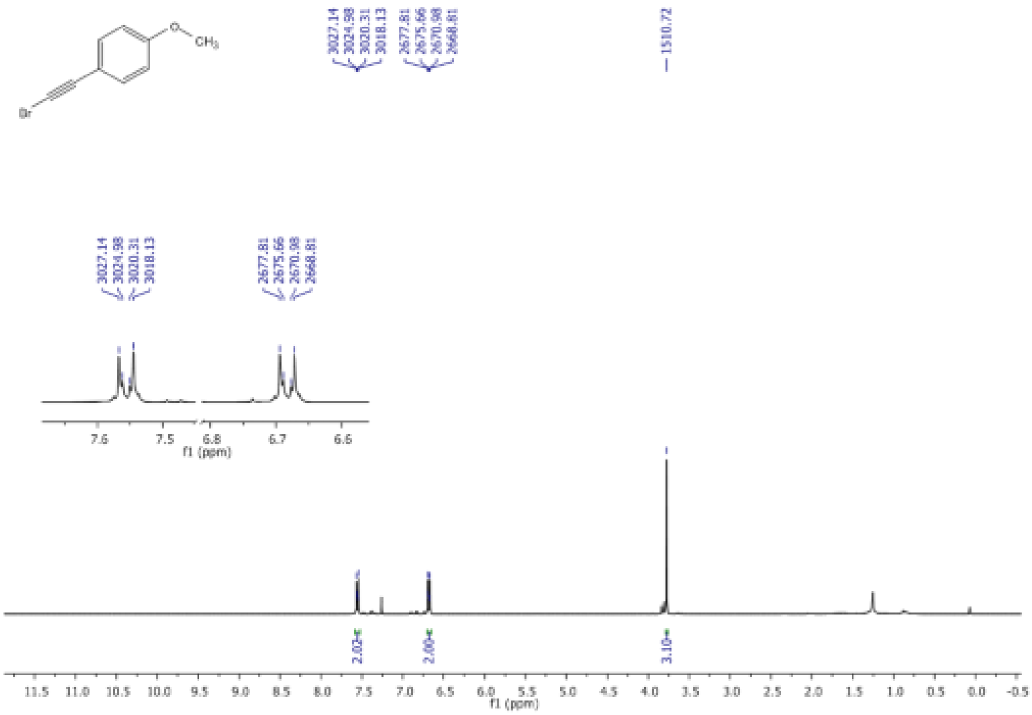

^{13}C-NMR: (100 MHz, $CDCl_3$)

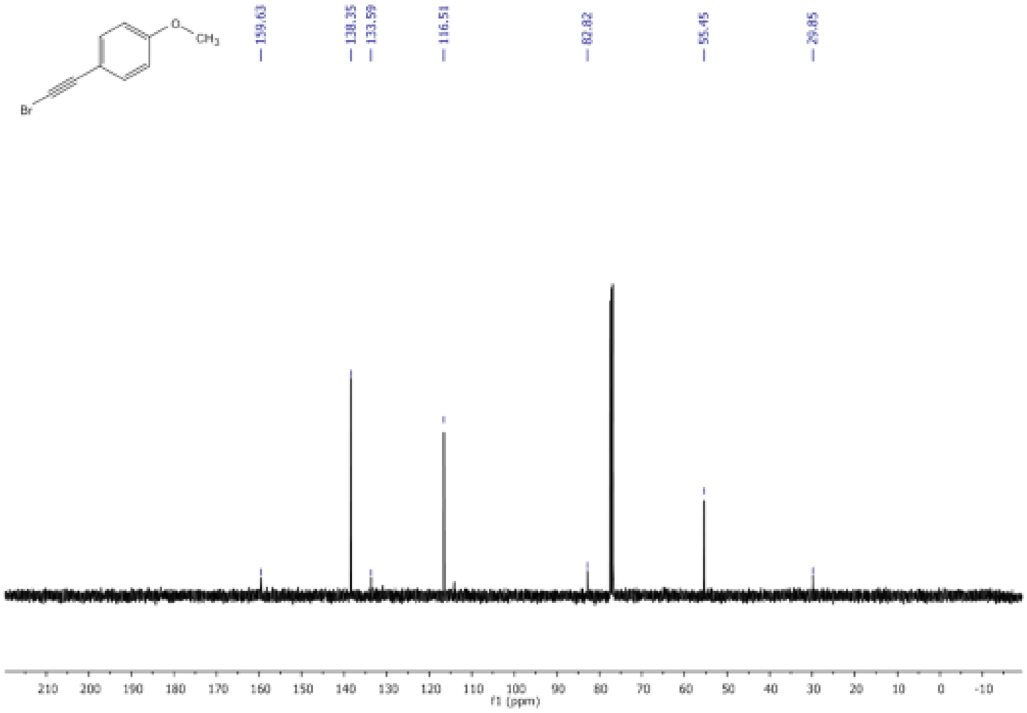

1-(Bromoethynyl)-4-nitrobenzene (**11b**).

^{1}H-NMR: (400 MHz, $CDCl_3$)

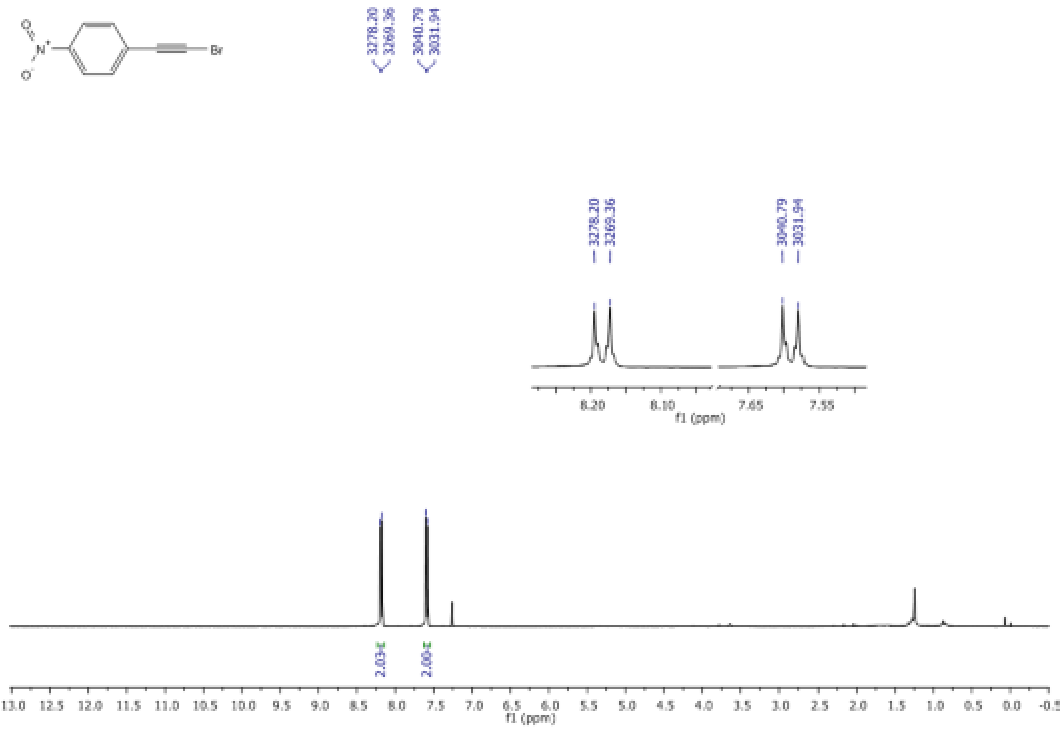

^{13}C-NMR: (100 MHz, $CDCl_3$)

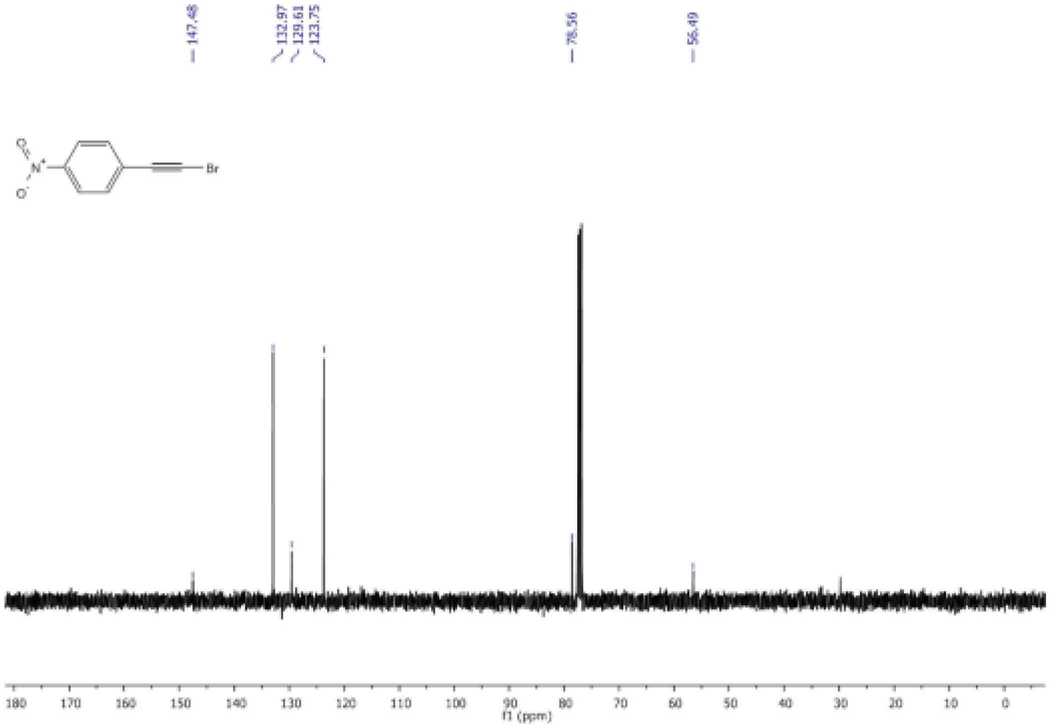

(Bromoethynyl)benzene (**11c**).

^{1}H-NMR: (400 MHz, CDCl$_3$)

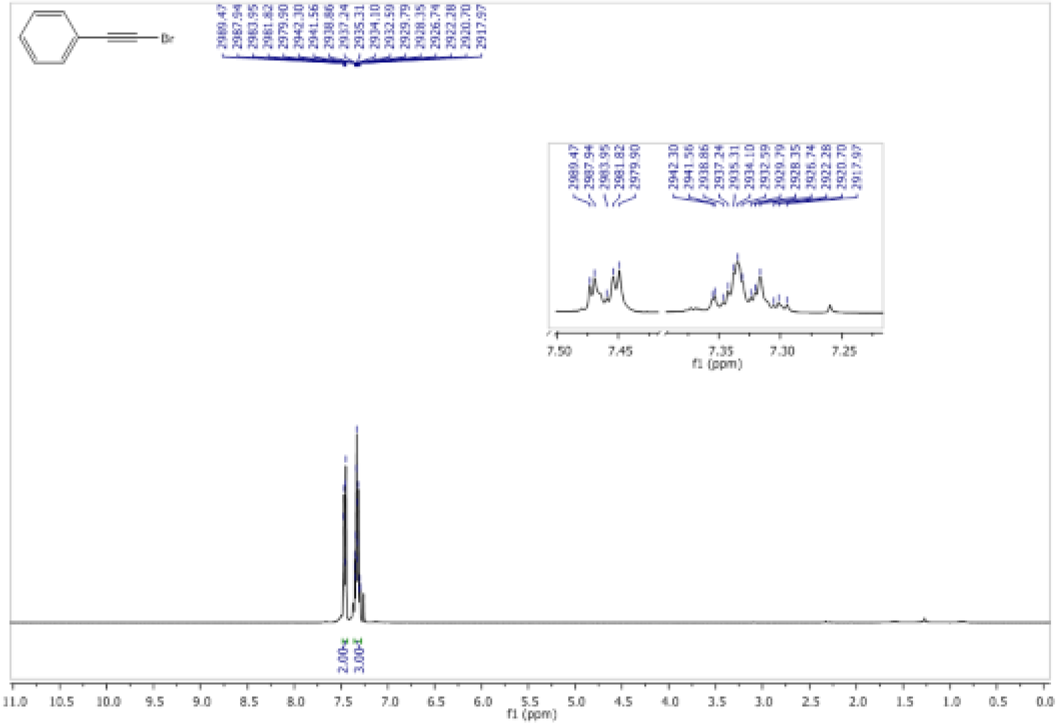

^{13}C-NMR: (100 MHz, CDCl$_3$)

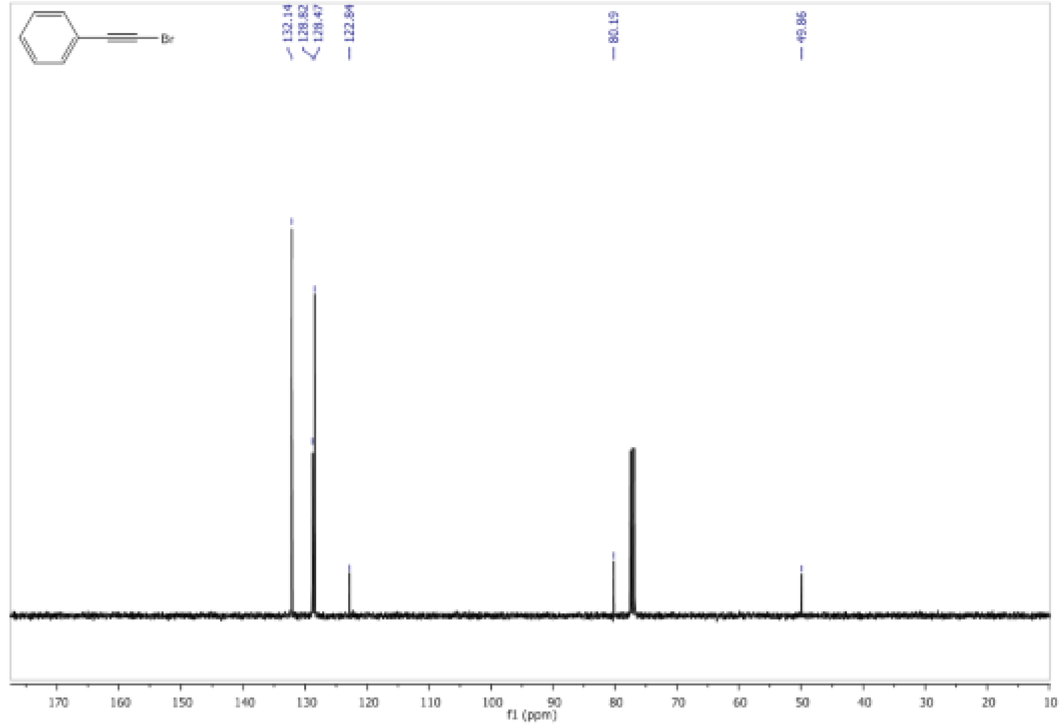

1-Bromohex-1-yne (**11d**).

^{1}H-NMR: (400 MHz, $CDCl_3$)

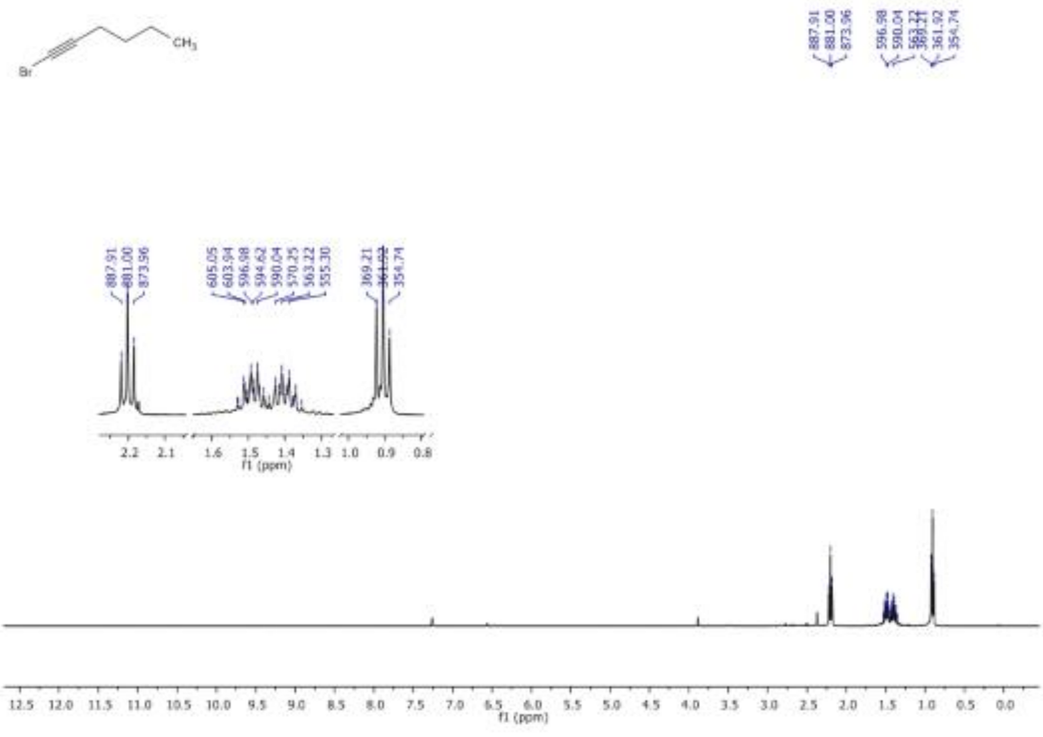

^{13}C-NMR: (100 MHz, $CDCl_3$)

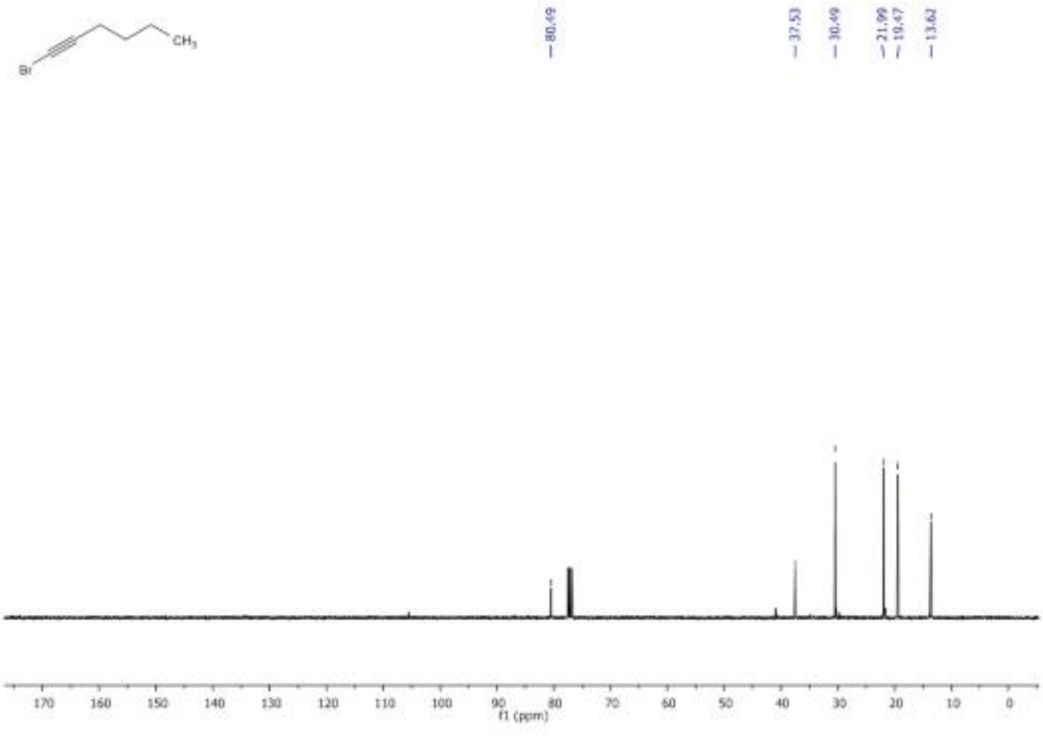

Methyl 1-[(4-methoxyphenyl)ethynyl]-1*H*-pyrrole-2-carboxylate (**7a**).

^{1}H-NMR: (400 MHz, $CDCl_3$)

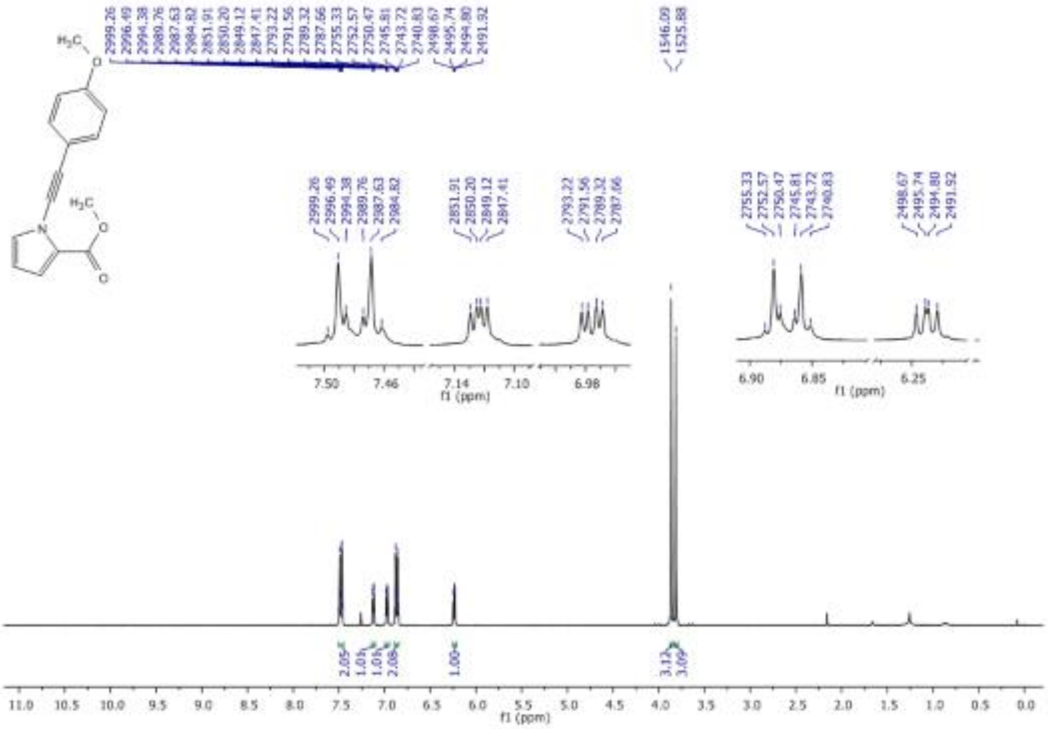

^{13}C-NMR: (100 MHz, $CDCl_3$)

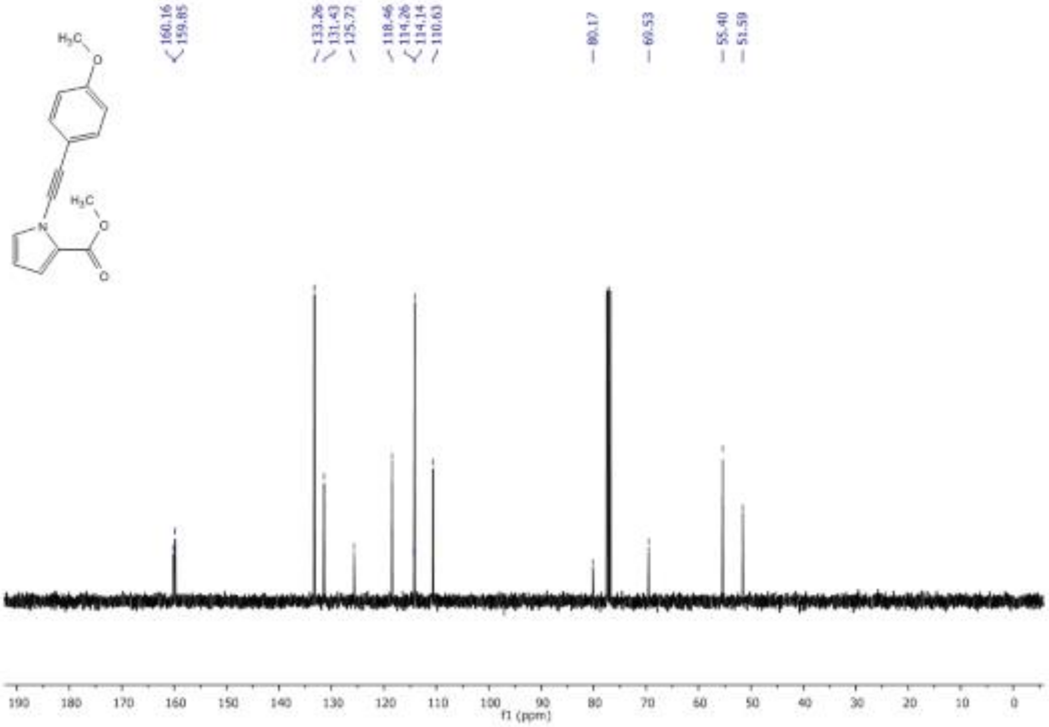

Methyl 1-[(4-nitrophenyl)ethynyl]-1*H*-pyrrole-2-carboxylate (**7b**).

^{1}H-NMR: (400 MHz, $CDCl_3$)

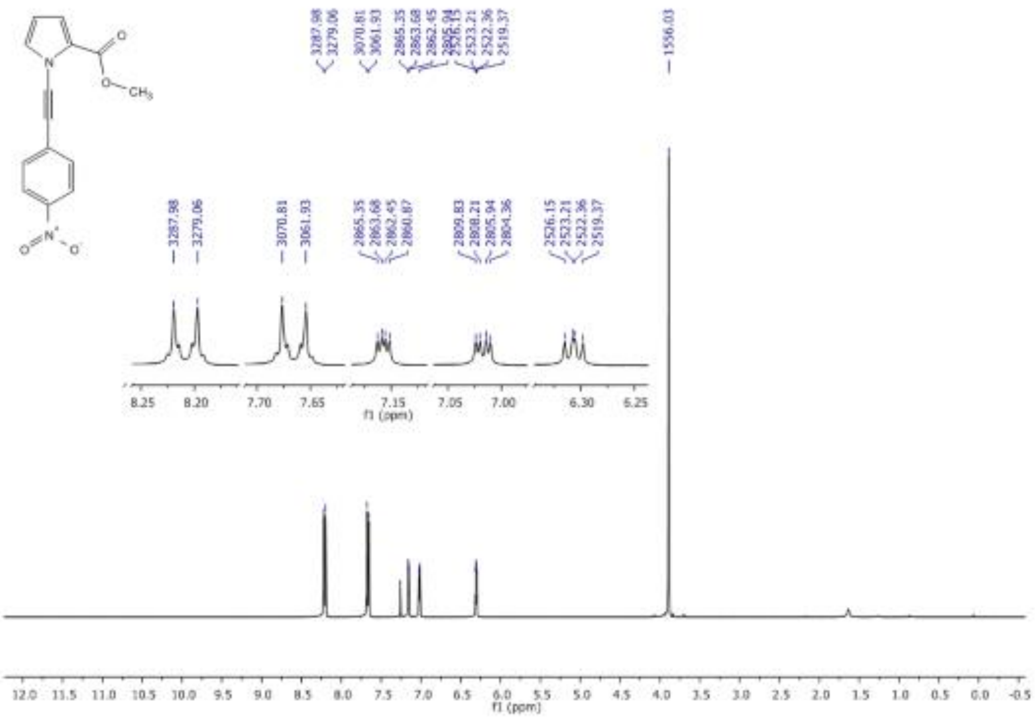

^{13}C-NMR: (100 MHz, $CDCl_3$)

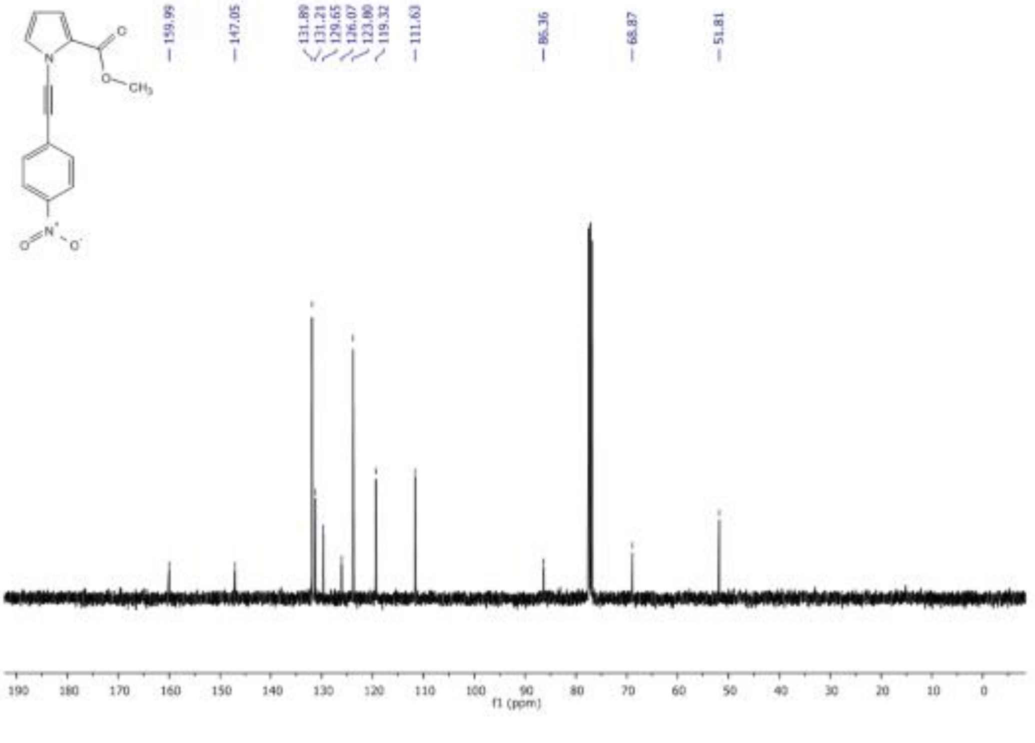

Methyl 1-(phenylethynyl)-1*H*-pyrrole-2-carboxylate (**7c**).

^{1}H-NMR: (400 MHz, $CDCl_3$)

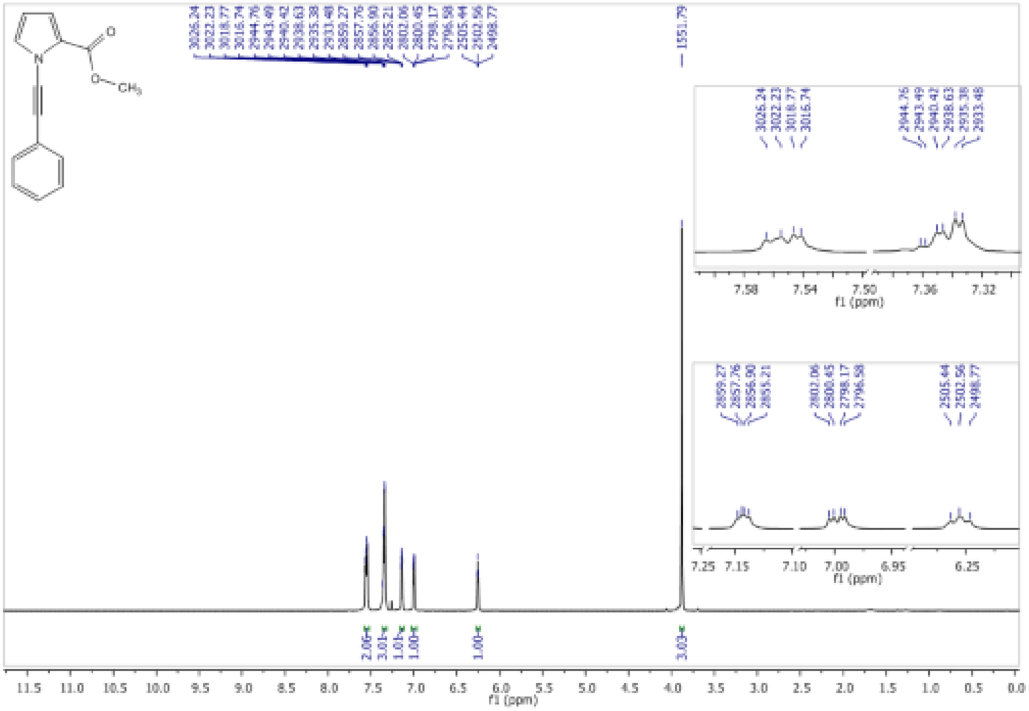

^{13}C-NMR: (100 MHz, $CDCl_3$)

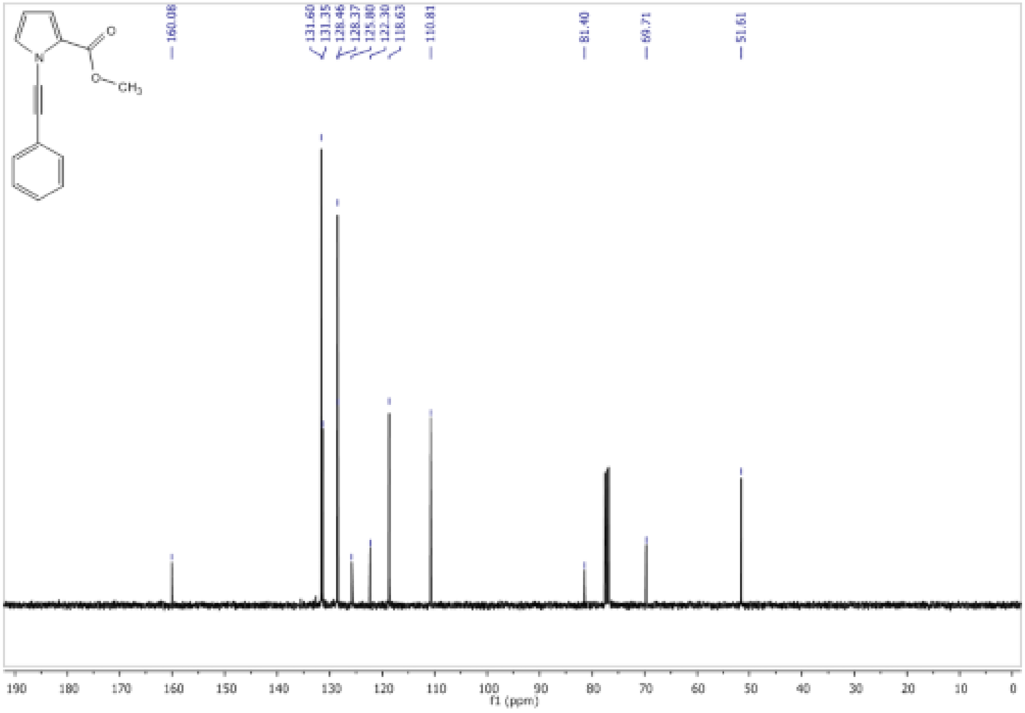

Methyl 1-(hex-1-yn-1-yl)-1*H*-pyrrole-2-carboxylate (**7d**).

^{1}H-NMR: (400 MHz, $CDCl_3$)

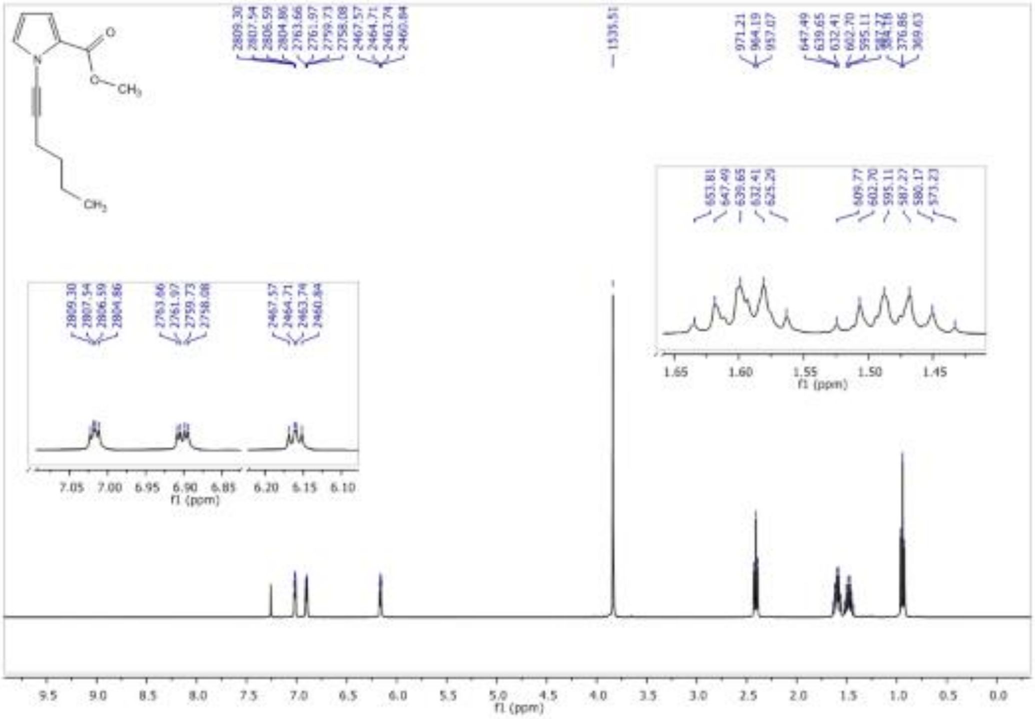

^{13}C-NMR: (100 MHz, $CDCl_3$)

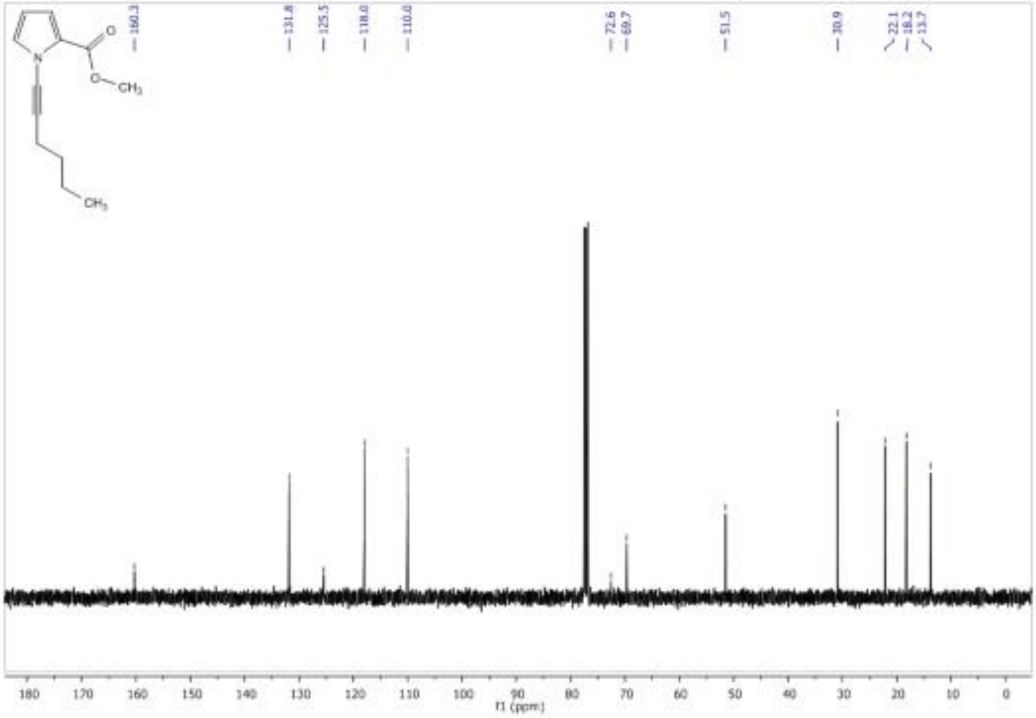

Methyl 1-(2-oxo-2-phenylethyl)-1*H*-pyrrole-2-carboxylate (**15**).

^{1}H-NMR: (400 MHz, $CDCl_3$)

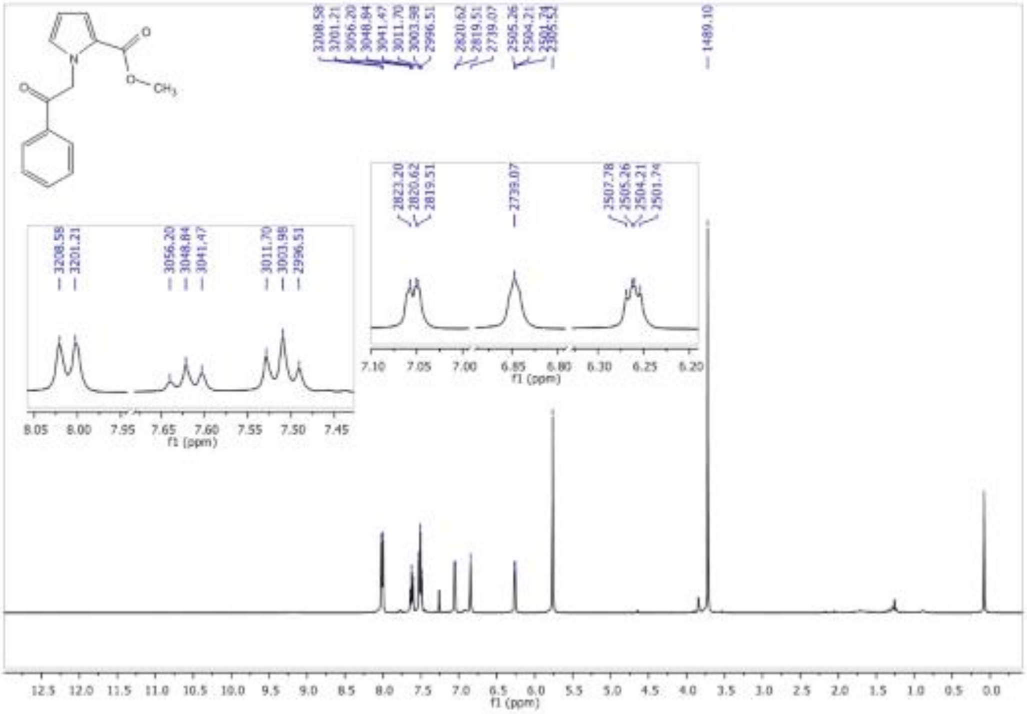

^{13}C-NMR: (100 MHz, $CDCl_3$)

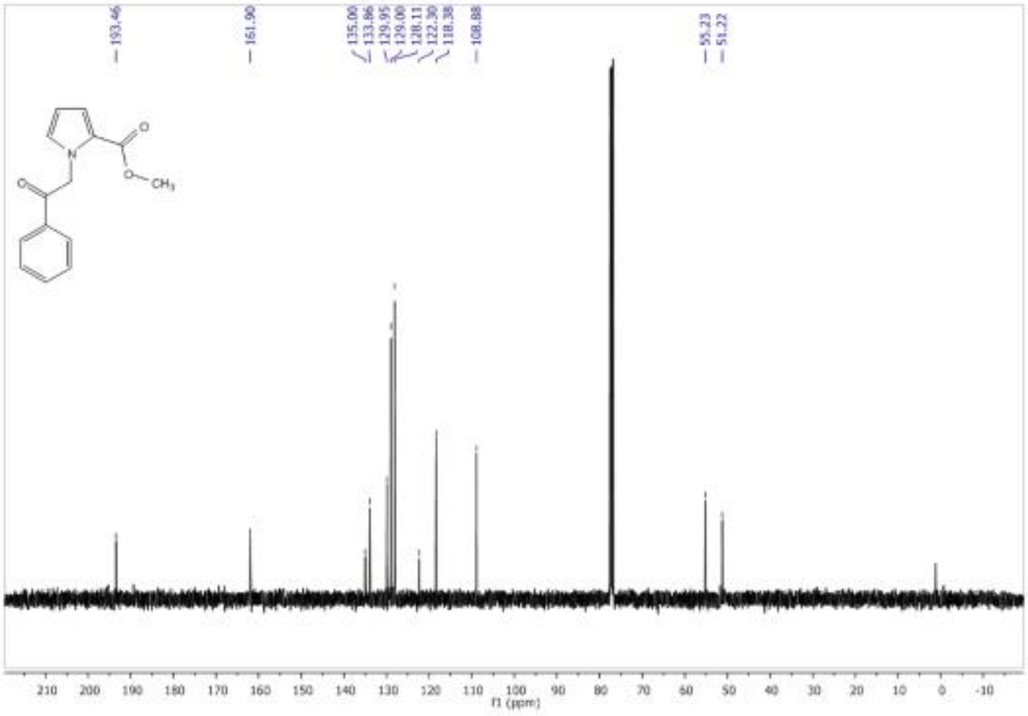

2-Amino-3-phenylpyrrolo[1,2-*a*]pyrazin-1-(2*H*)-one (**12c**).

^{1}H-NMR: (400 MHz, $CDCl_3$)

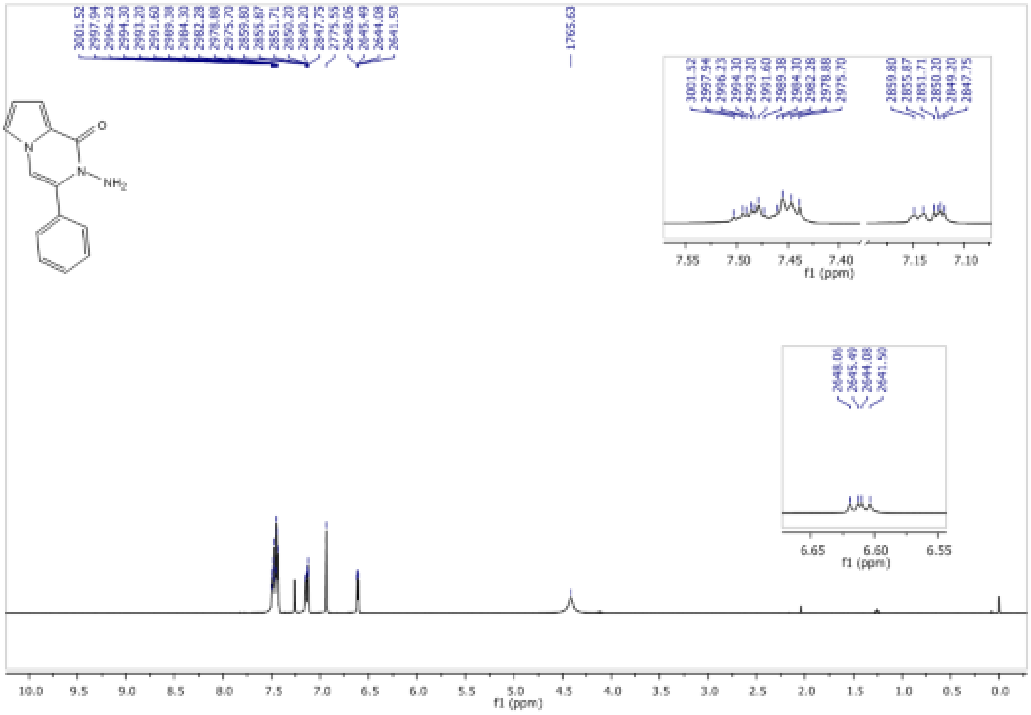

^{13}C-NMR: (100 MHz, $CDCl_3$)

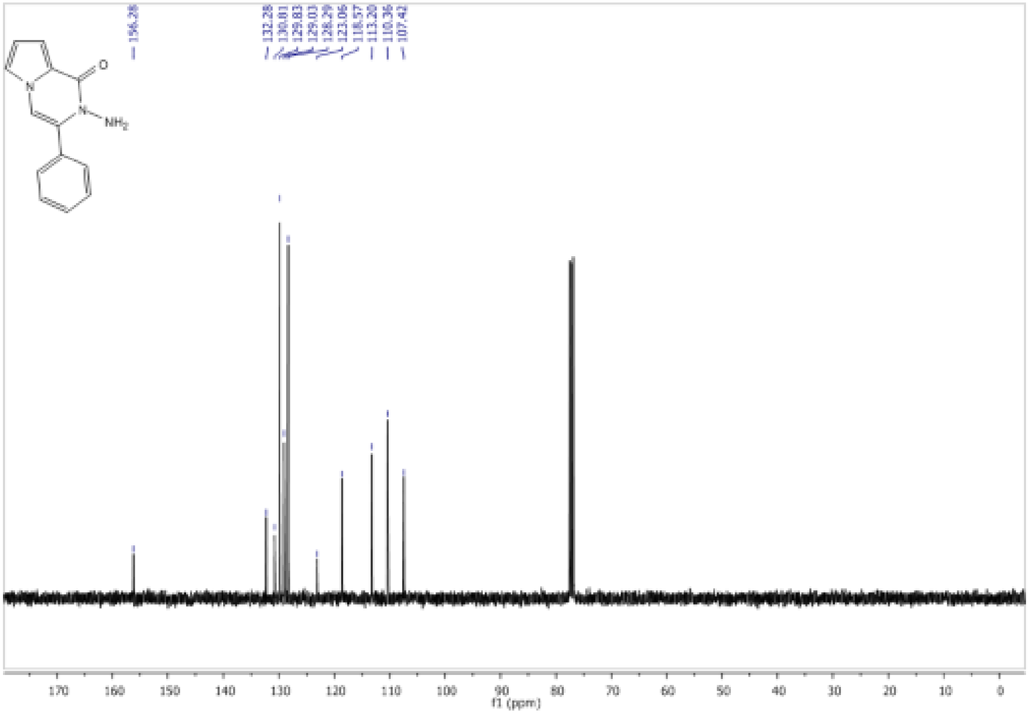

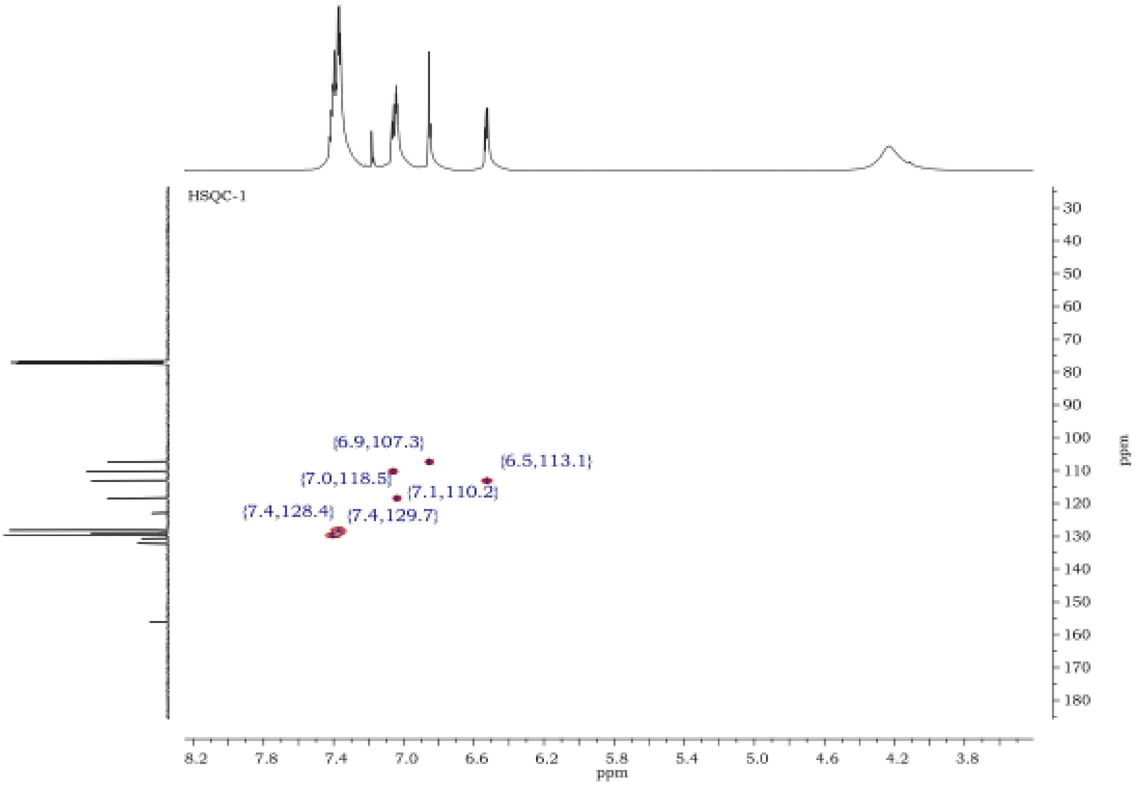
HSQC-1
{6.9,107.3}
{6.5,113.1}
{7.0,118.5}
{7.1,110.2}
{7.4,128.4}
{7.4,129.7}
ppm
ppm

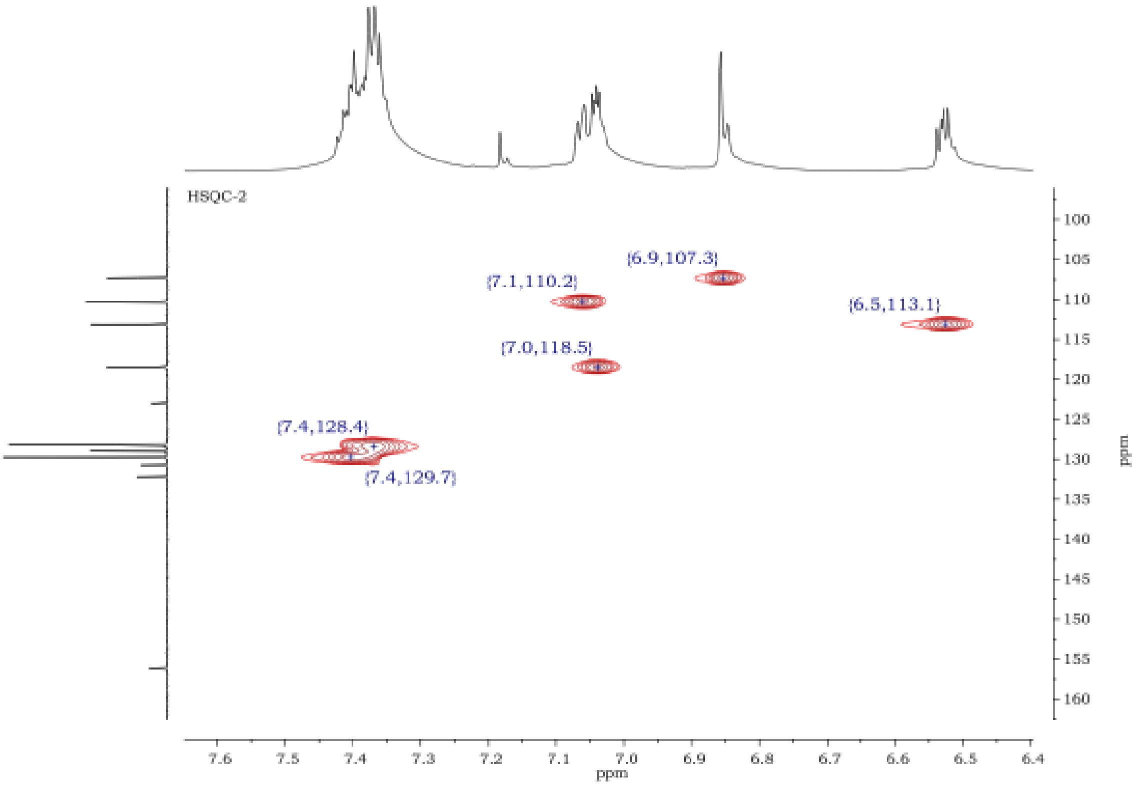
HSQC-2
{6.9,107.3}
{7.1,110.2}
{6.5,113.1}
{7.0,118.5}
{7.4,128.4}
{7.4,129.7}
ppm
ppm

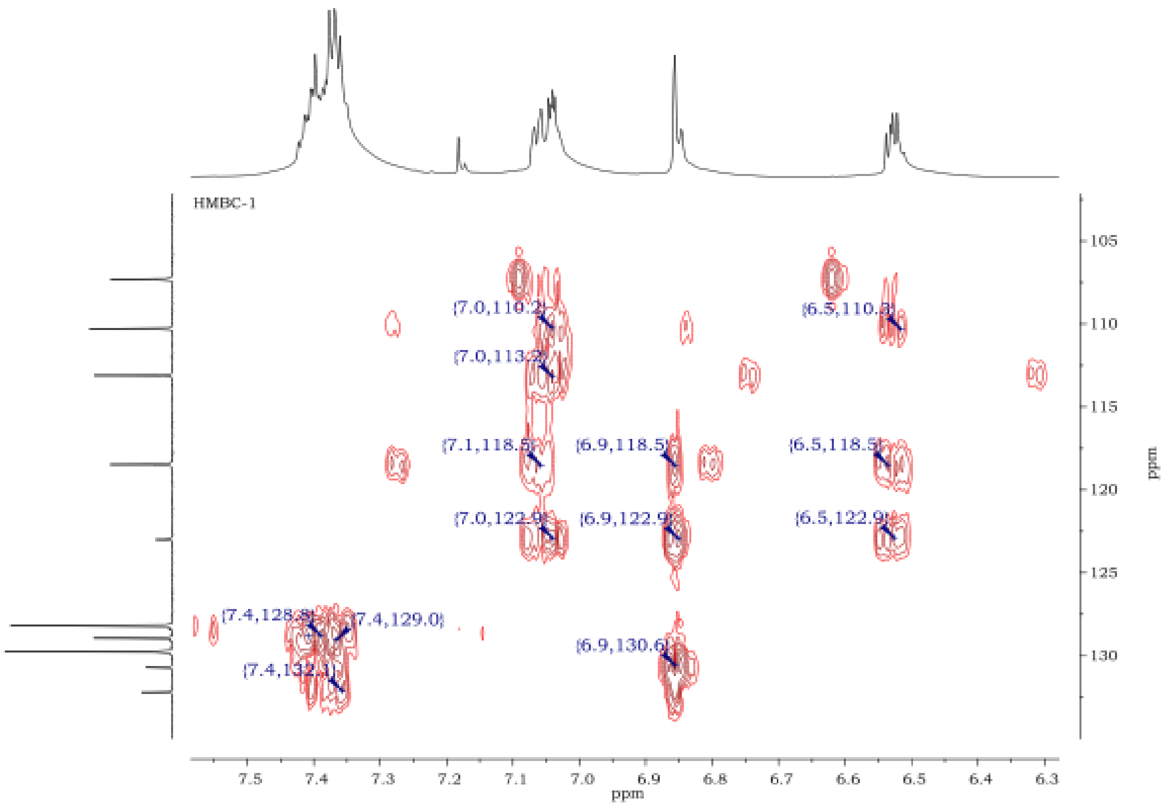
HMBC-1
{7.0,110.2}
{7.0,113.2}
{6.5,110.3}
{7.1,118.5}
{6.9,118.5}
{6.5,118.5}
{7.0,122.9}
{6.9,122.9}
{6.5,122.9}
{7.4,128.8}
{7.4,129.0}
{7.4,132.1}
{6.9,130.6}
ppm
ppm

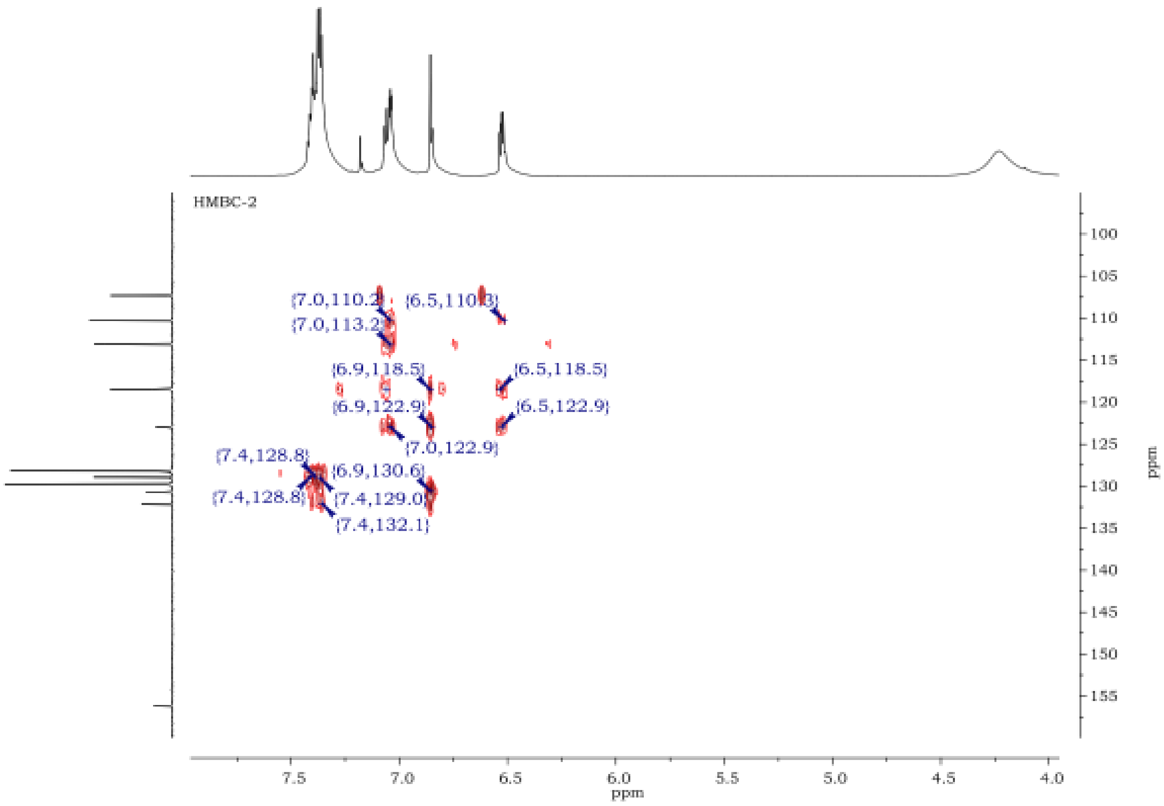
HMBC-2
{7.0,110.2}
{6.5,110.3}
{7.0,113.2}
{6.9,118.5}
{6.5,118.5}
{6.9,122.9}
{6.5,122.9}
{7.0,122.9}
{7.4,128.8}
{6.9,130.6}
{7.4,128.8}
{7.4,129.0}
{7.4,132.1}
ppm
ppm

4-Benzylpyrrolo[1,2-*d*][1,2,4]triazin-1(2*H*)-one (**13c**).

^{1}H-NMR: (400 MHz, DMSO)

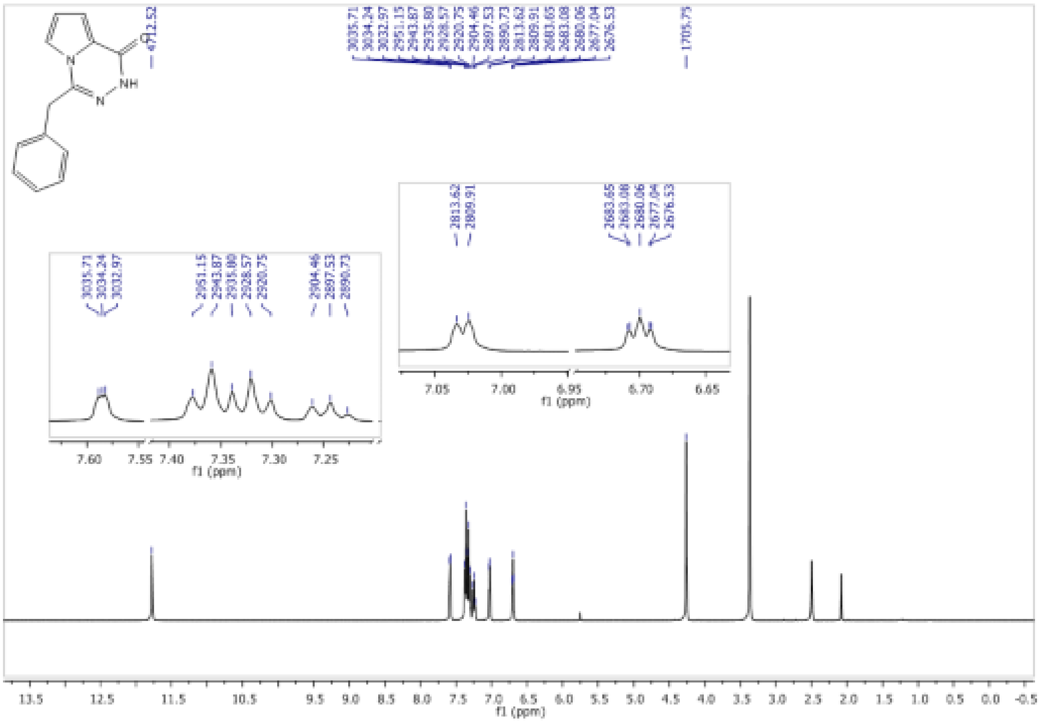

^{13}C-NMR: (100 MHz, DMSO)

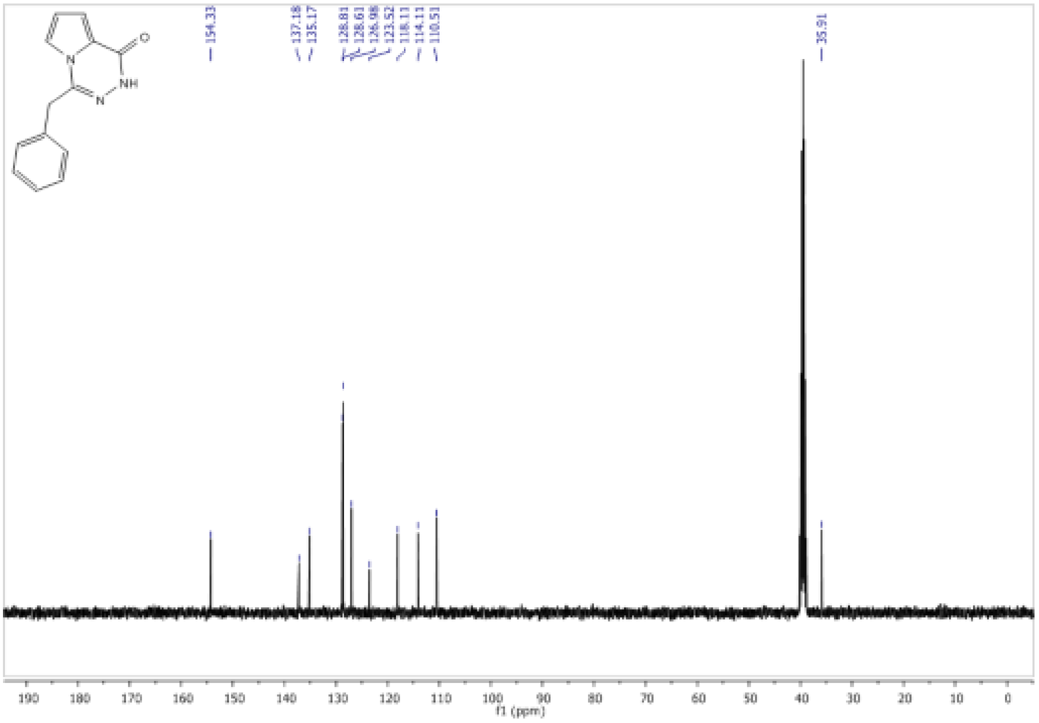

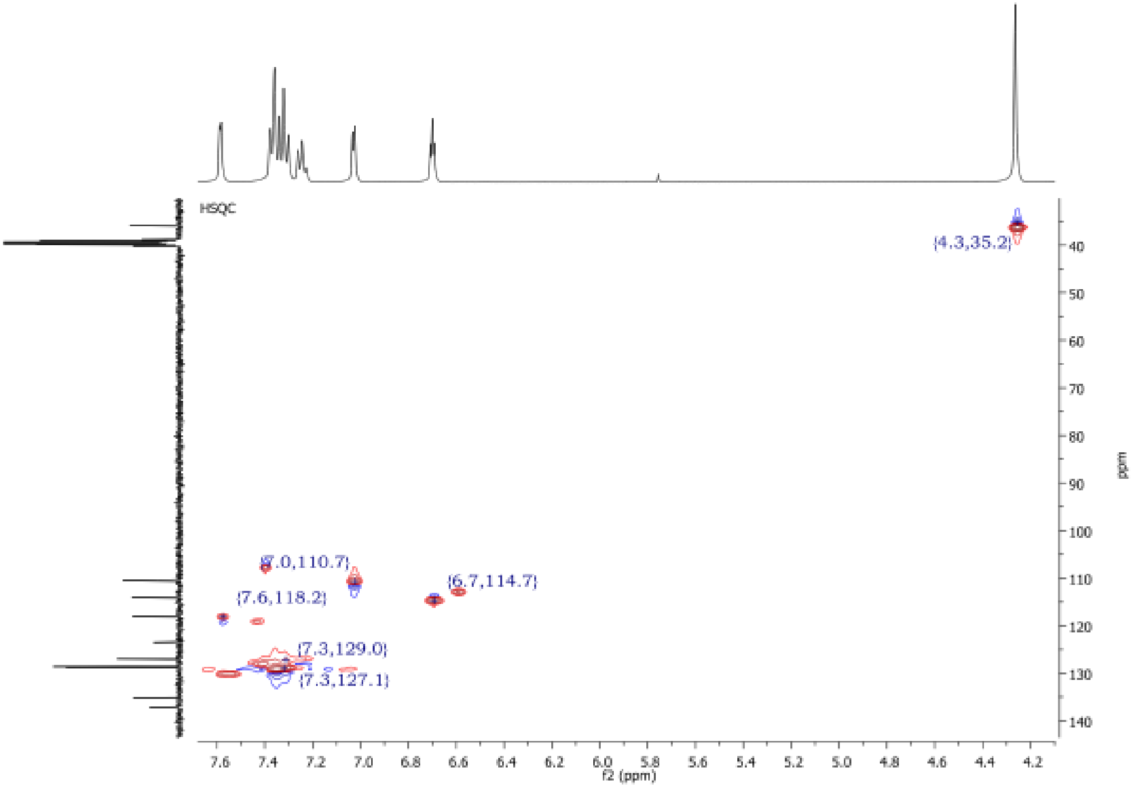
HSQC
{4.3,35.2}
{7.0,110.7}
{6.7,114.7}
{7.6,118.2}
{7.3,129.0}
{7.3,127.1}
f2 (ppm)
ppm

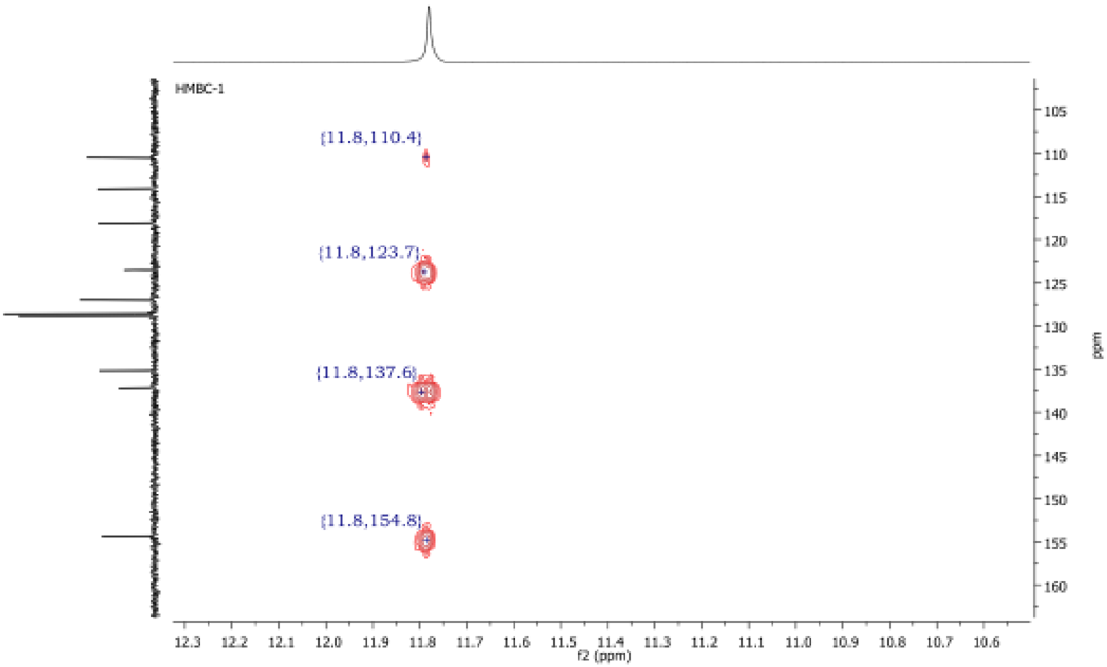
HMBC-1
{11.8,110.4}
{11.8,123.7}
{11.8,137.6}
{11.8,154.8}
f2 (ppm)
ppm

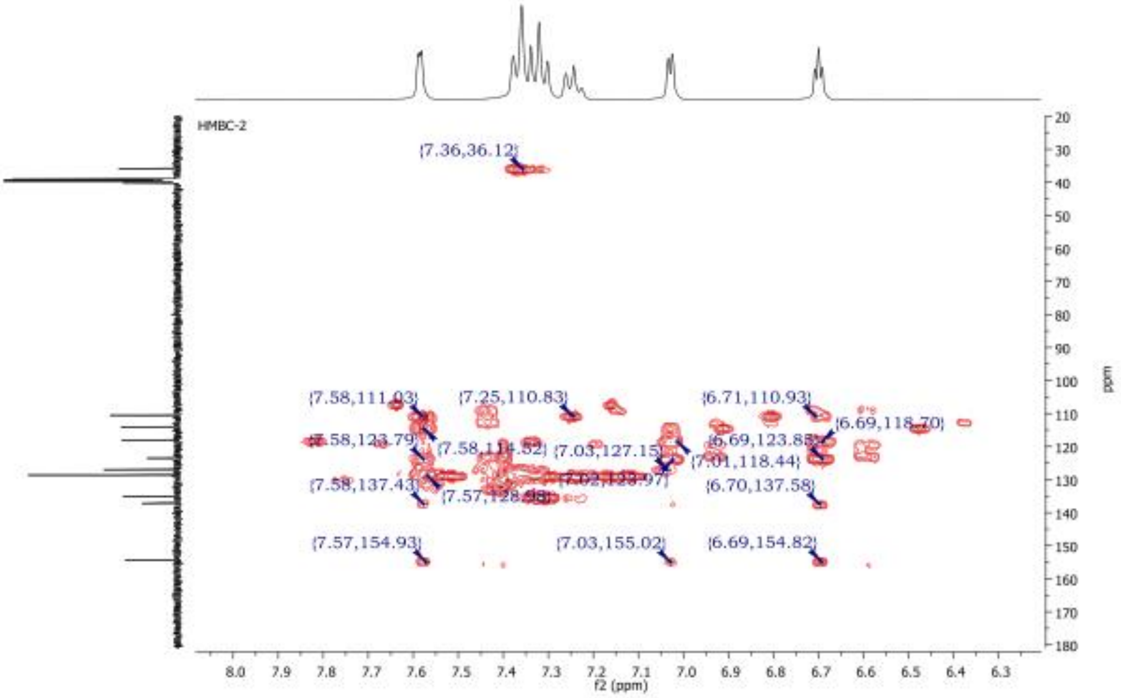
HMBC-2
{7.36,36.12}
{7.58,111.03}
{7.25,110.83}
{6.71,110.93}
{6.69,118.70}
{7.58,114.52}
{7.03,127.15}
{6.69,123.83}
{7.01,118.44}
{7.58,137.43}
{7.57,128.98}
{7.02,128.97}
{6.70,137.58}
{7.57,154.93}
{7.03,155.02}
{6.69,154.82}
f2 (ppm)
ppm

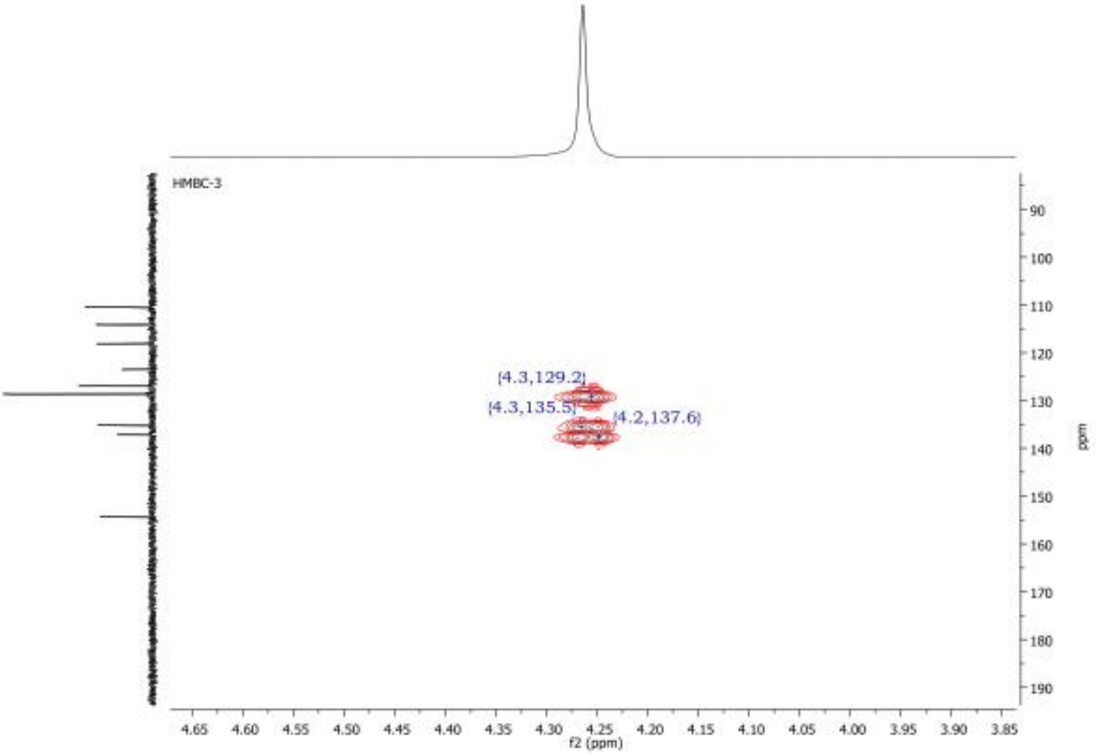
HMBC-3
{4.3,129.2}
{4.3,135.5}
{4.2,137.6}
f2 (ppm)
ppm

N-(1-oxo-3-phenylpyrrolo[1,2-*a*]pyrazin-2(1*H*)-yl)acetamide (**14**).

^{1}H-NMR: (400 MHz, $CDCl_3$)

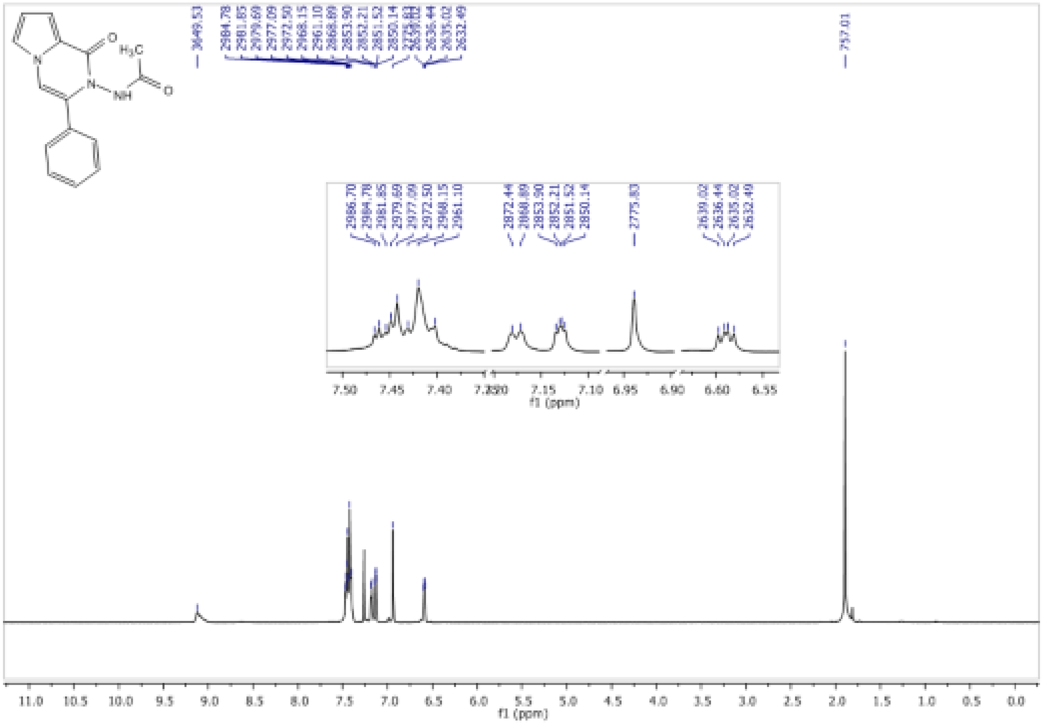

^{13}C-NMR: (100 MHz, $CDCl_3$)

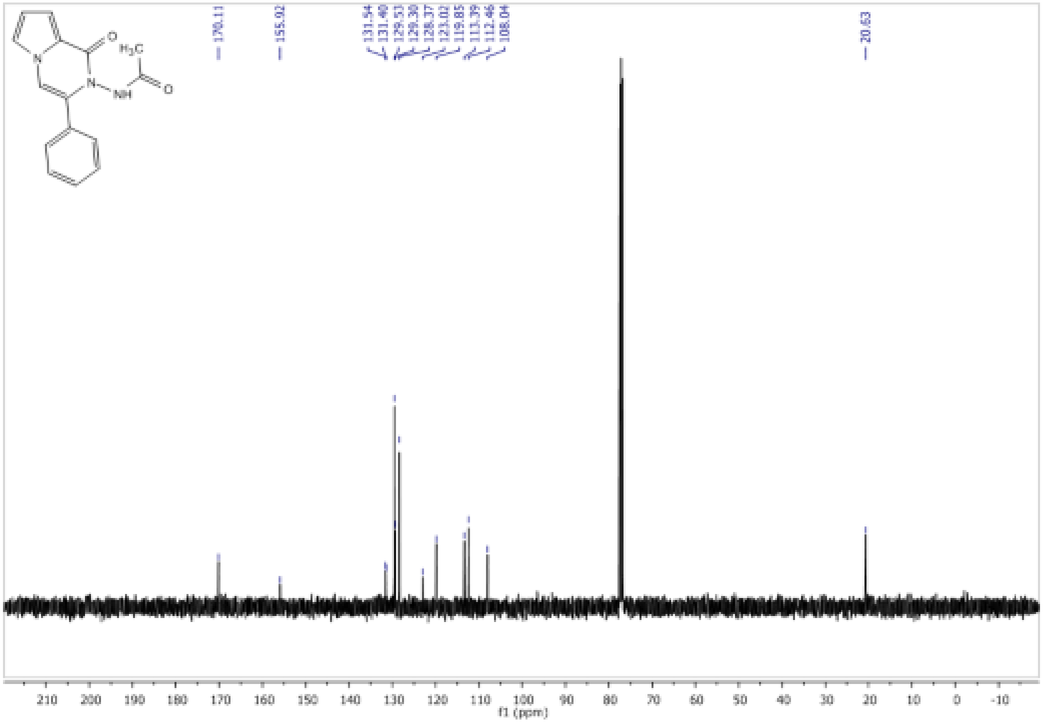

4-Phenyl-2,5-dihydro-1*H*-pyrrolo[2,1-*d*][1,2,5]triazepin-1-one (**16**).

^{1}H-NMR: (400 MHz, DMSO)

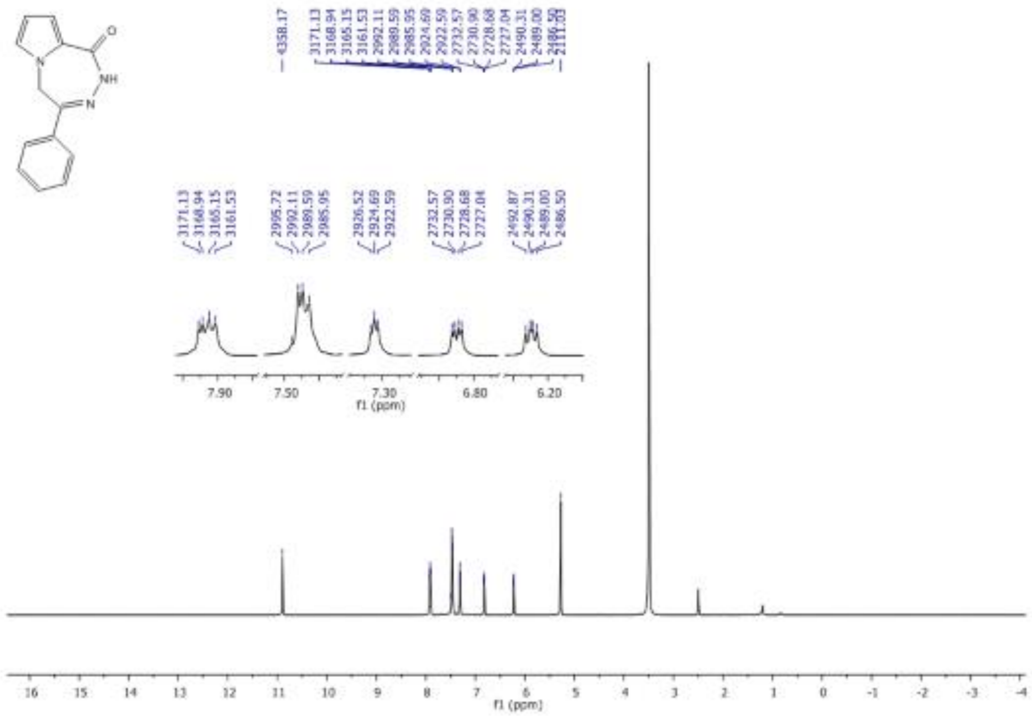

^{13}C-NMR: (100 MHz, DMSO)

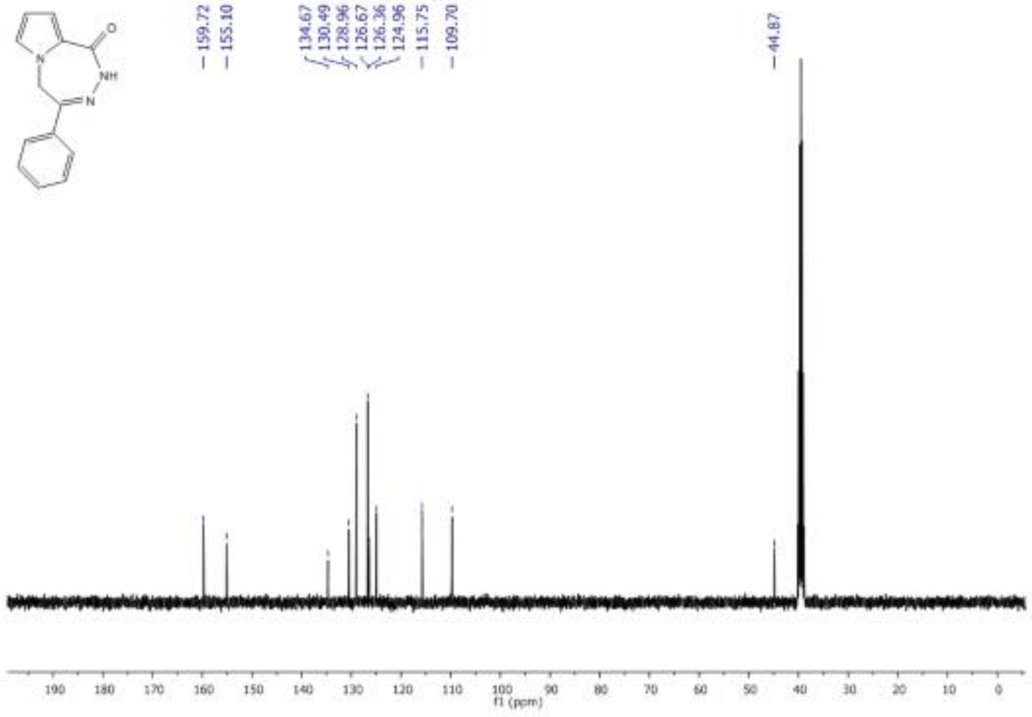

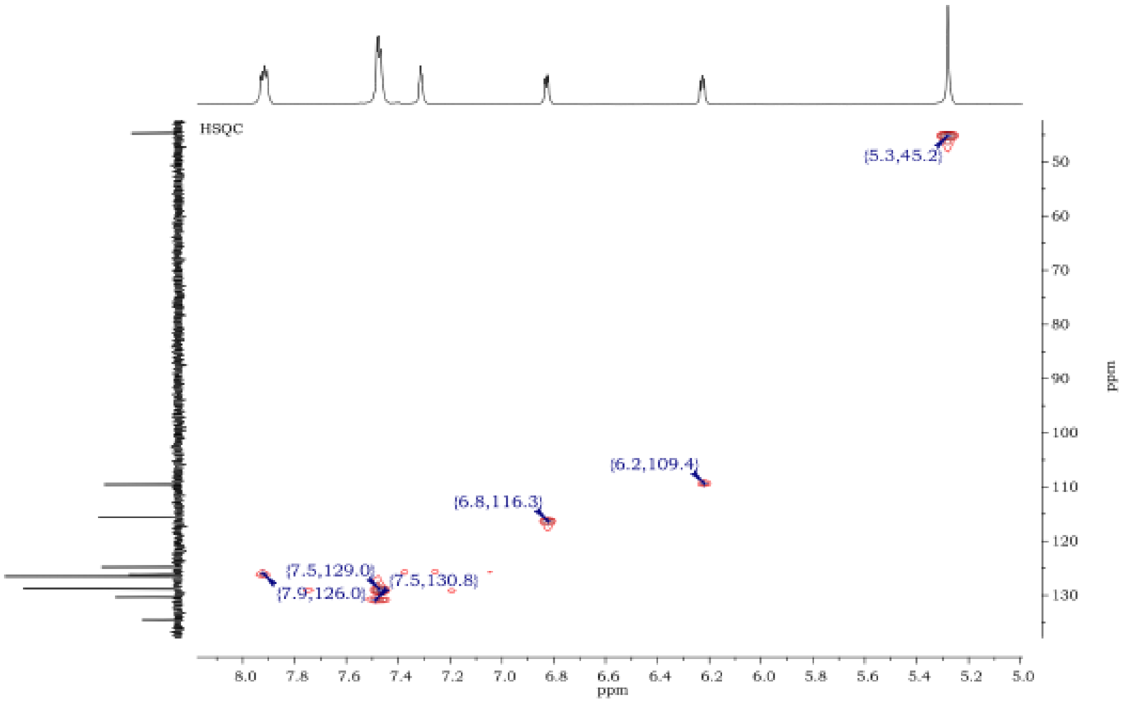
HSQC
{5.3,45.2}
{6.2,109.4}
{6.8,116.3}
{7.5,129.0}
{7.9,126.0}
{7.5,130.8}
ppm

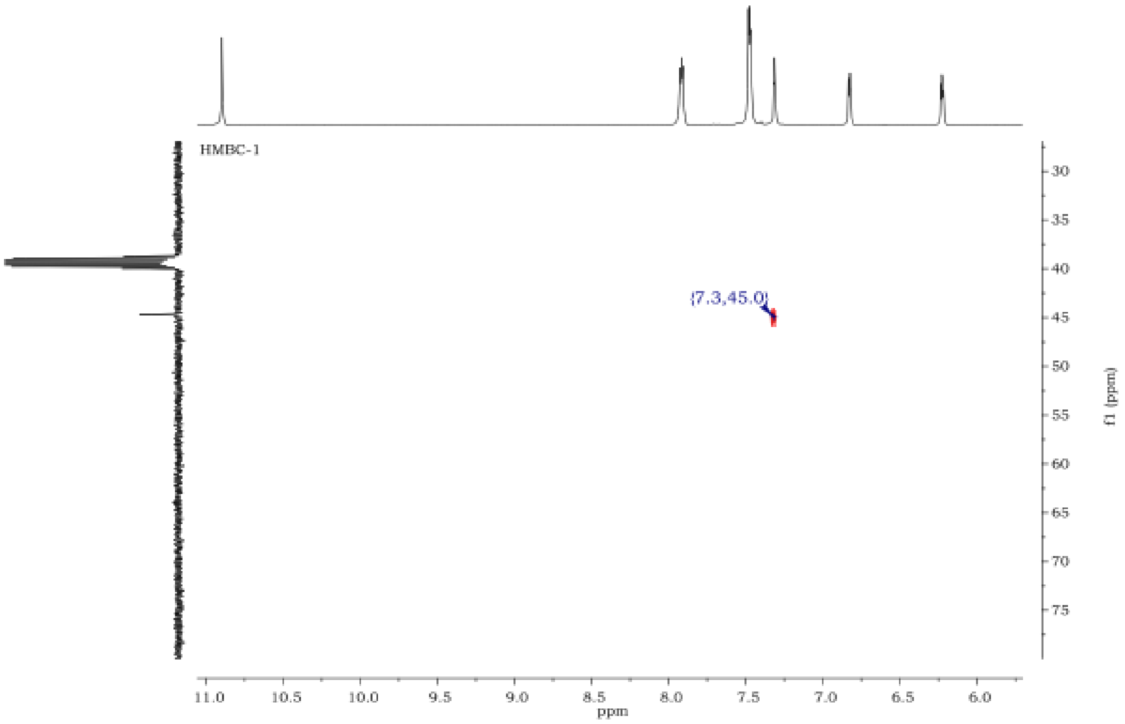
HMBC-1
{7.3,45.0}
ppm
f1 (ppm)

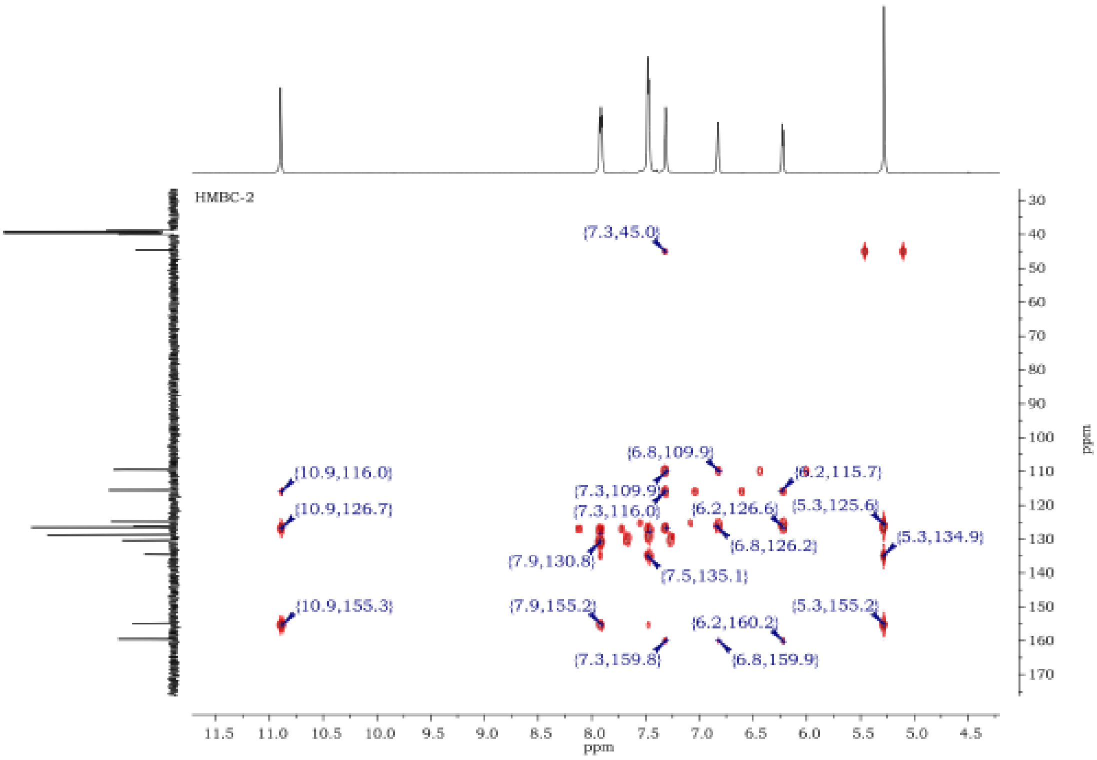
HMBC-2
{7.3,45.0}
{6.8,109.9}
{10.9,116.0}
{6.2,115.7}
{7.3,109.9}
{10.9,126.7}
{7.3,116.0}
{6.2,126.6}
{5.3,125.6}
{5.3,134.9}
{6.8,126.2}
{7.9,130.8}
{7.5,135.1}
{10.9,155.3}
{7.9,155.2}
{5.3,155.2}
{6.2,160.2}
{7.3,159.8}
{6.8,159.9}
ppm
ppm

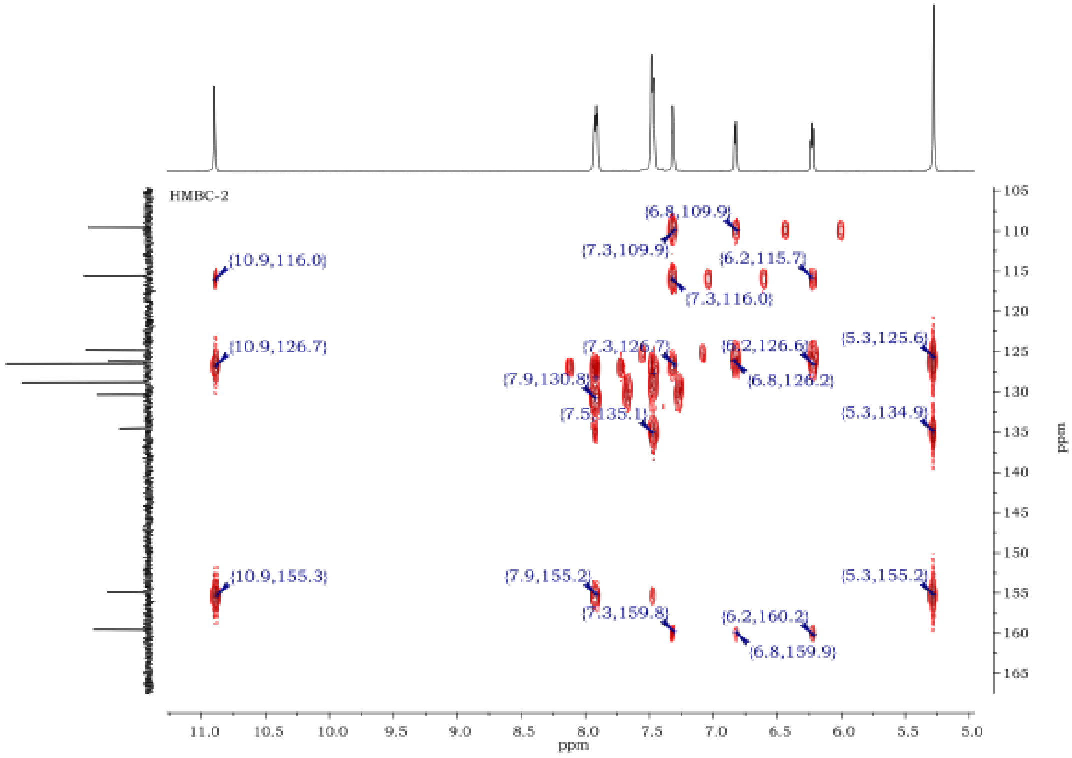
HMBC-2
{6.8,109.9}
{10.9,116.0}
{7.3,109.9}
{6.2,115.7}
{7.3,116.0}
{10.9,126.7}
{7.3,126.7}
{6.2,126.6}
{5.3,125.6}
{7.9,130.8}
{6.8,126.2}
{7.5,135.1}
{5.3,134.9}
{10.9,155.3}
{7.9,155.2}
{5.3,155.2}
{7.3,159.8}
{6.2,160.2}
{6.8,159.9}
ppm
ppm

2-Amino-3-(4-methoxyphenyl)pyrrolo[1,2-*a*]pyrazin-1(2*H*)-one (**12a**).

^{1}H-NMR: (400 MHz, $CDCl_3$)

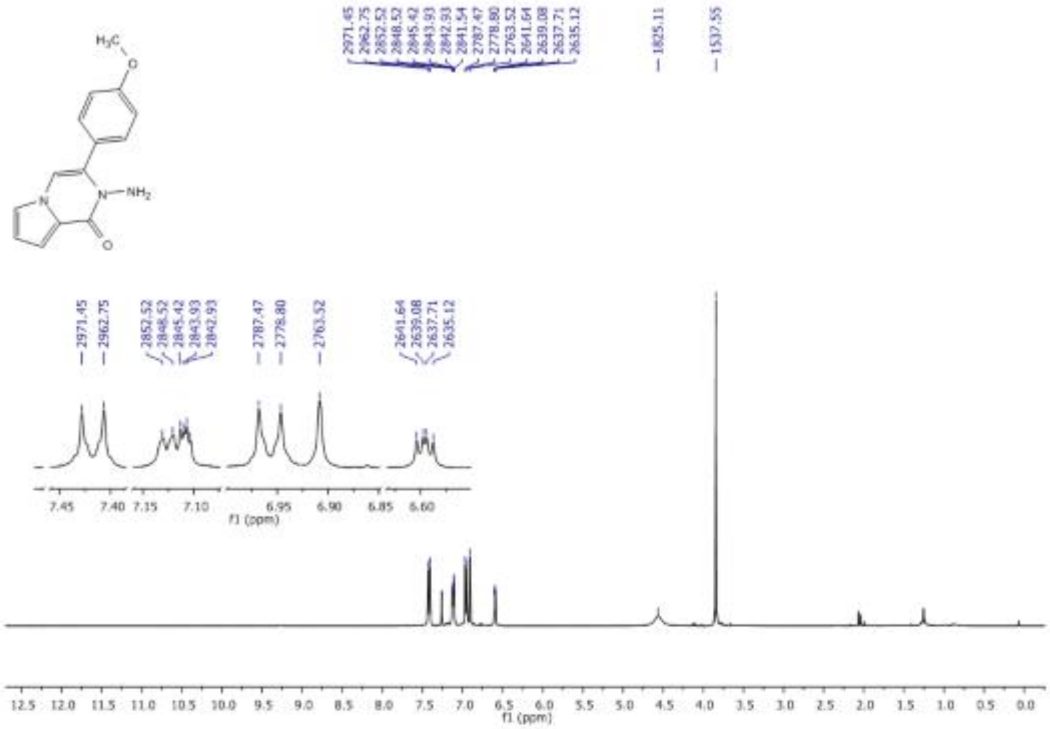

^{13}C-NMR: (100 MHz, $CDCl_3$)

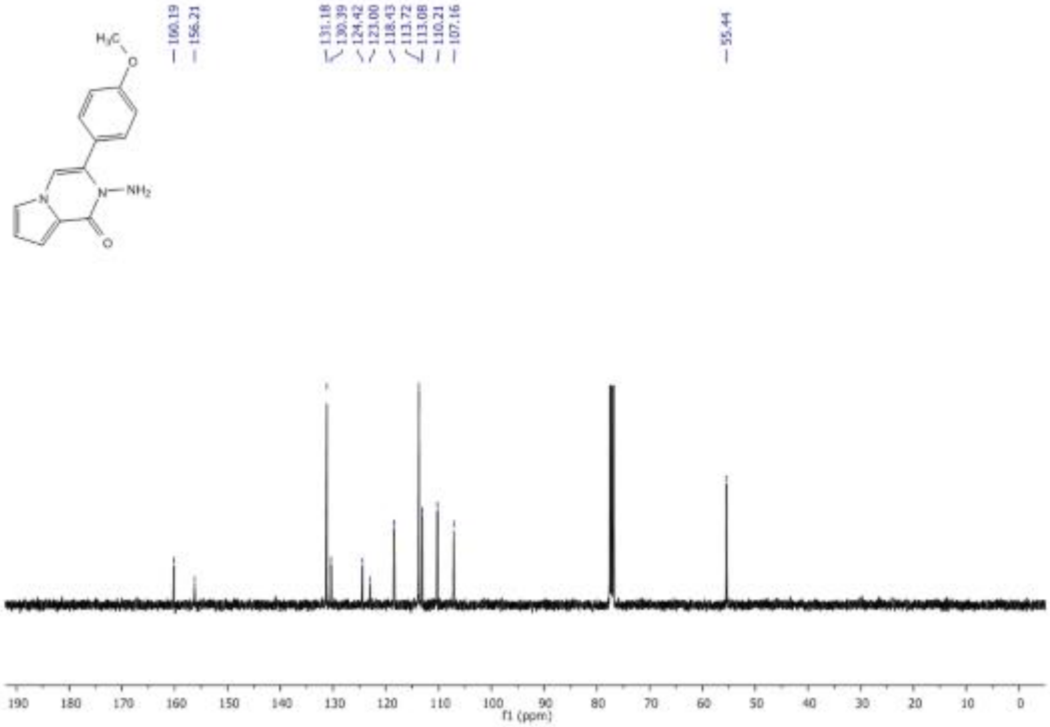

4-(4-Nitrobenzyl)pyrrolo[1,2-*d*][1,2,4]triazin-1(2*H*)-one (**13b**).

^{1}H-NMR: (400 MHz, DMSO)

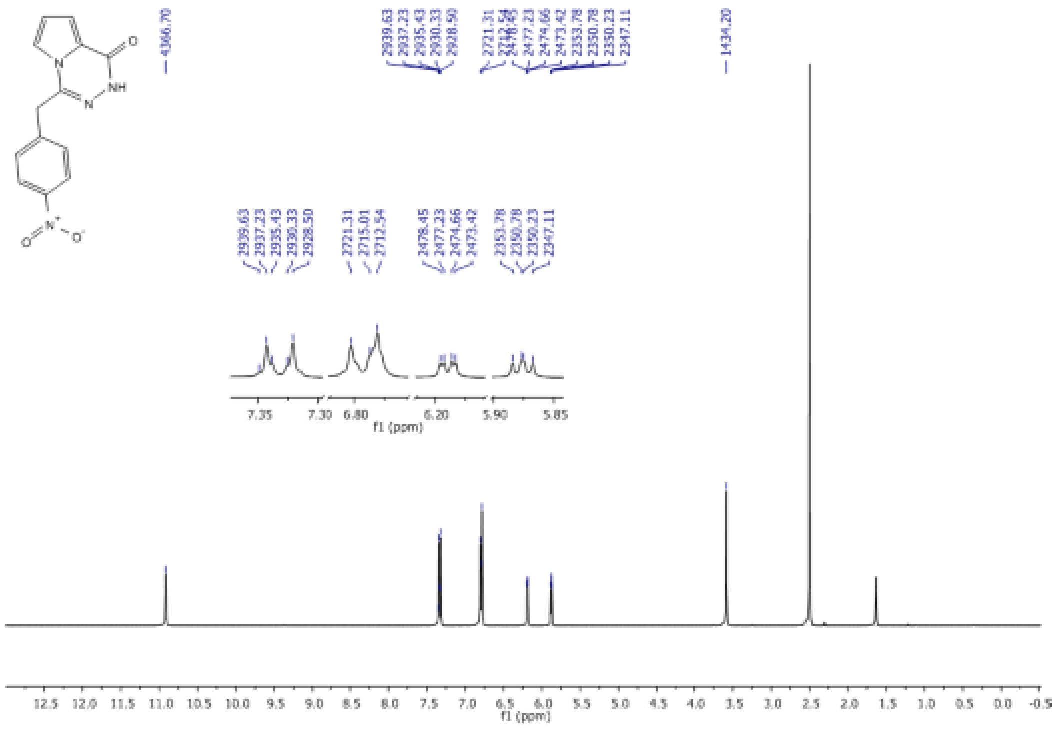

^{13}C-NMR: (100 MHz, DMSO)

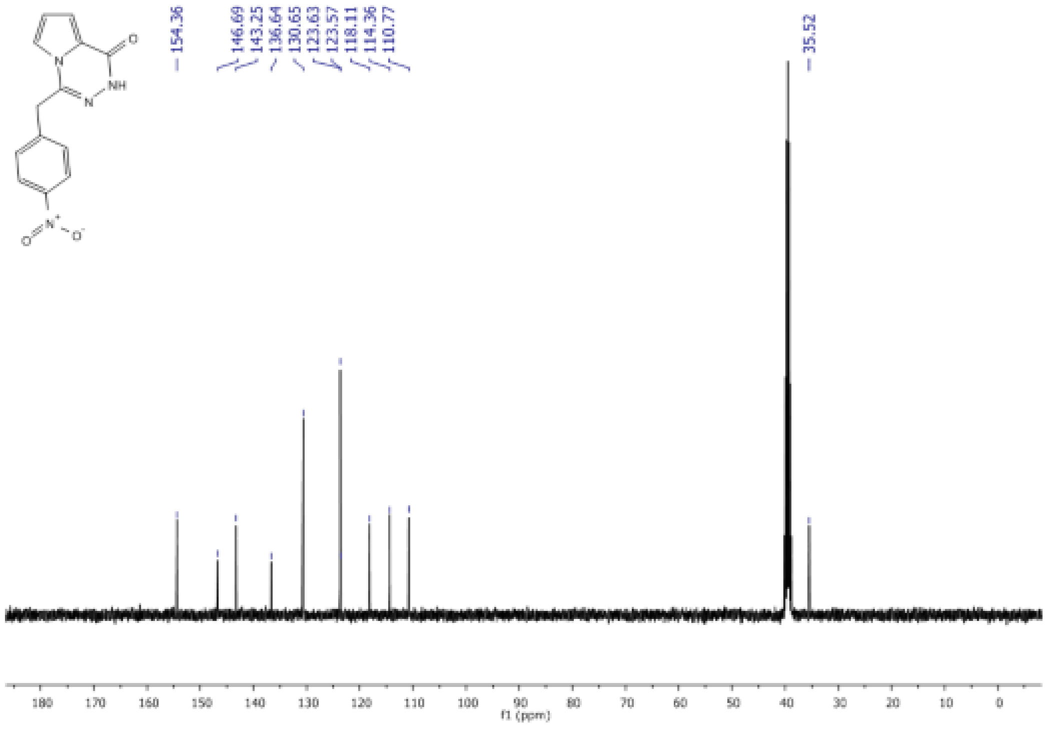

2-Amino-3-butylpyrrolo[1,2-*a*]pyrazin-1(2*H*)-one (**12d**).

^{1}H-NMR: (400 MHz, $CDCl_3$)

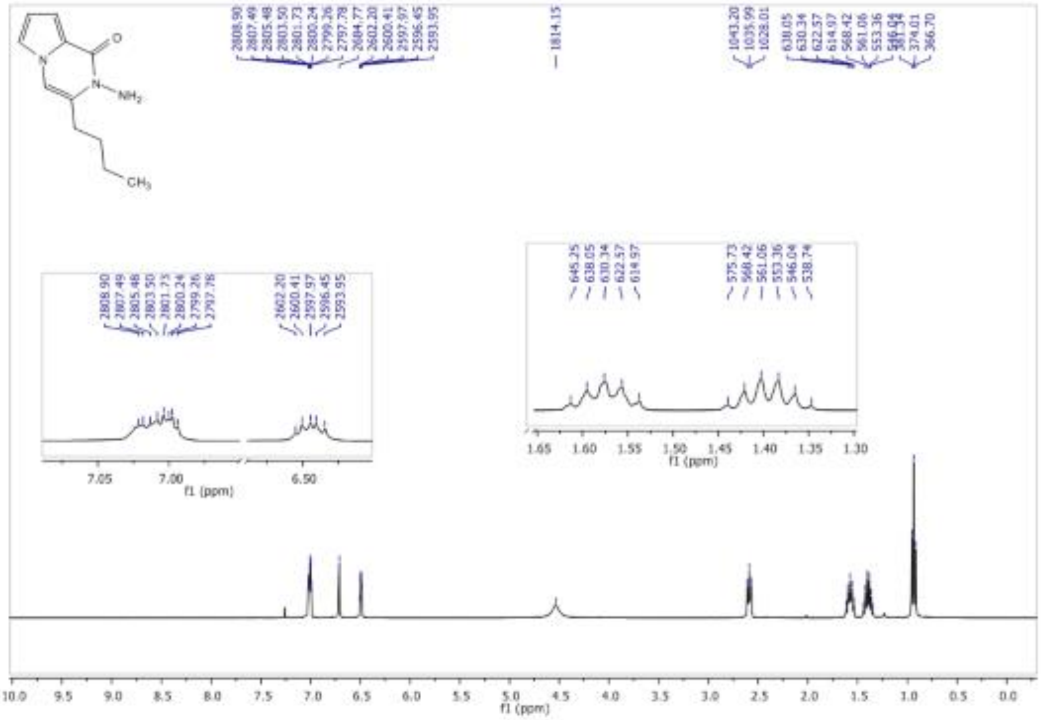

^{13}C-NMR: (100 MHz, $CDCl_3$)

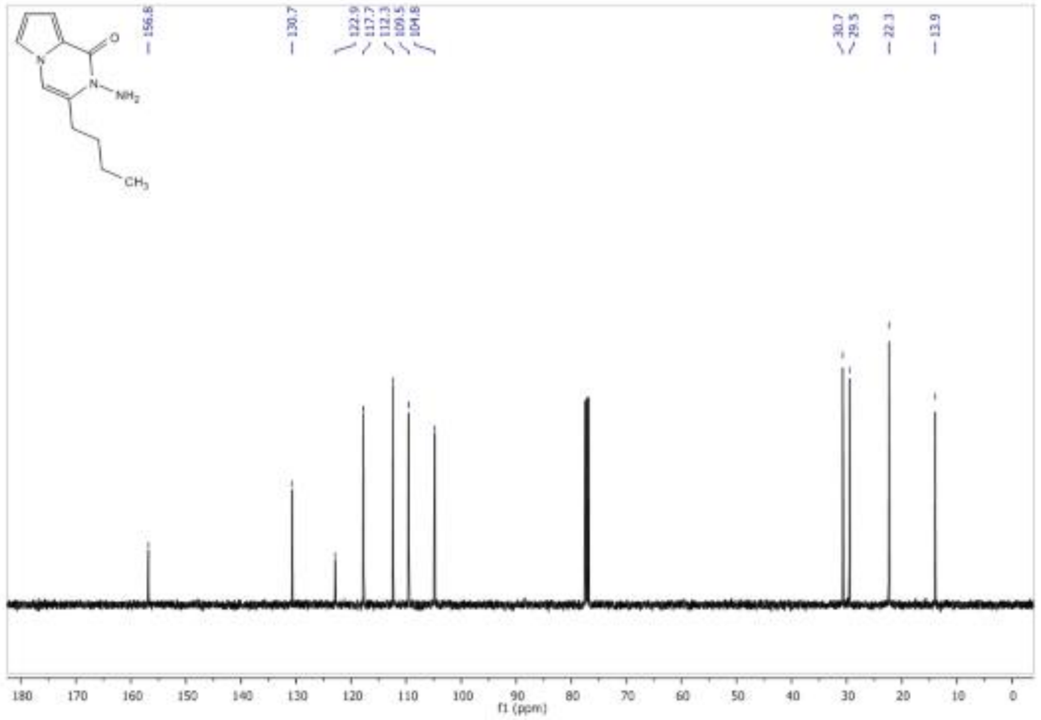

4-Iodo-3-phenyl-1*H*-pyrrolo[2,1-*c*][1,4]oxazin-1-one (**19c**).

^{1}H-NMR: (400 MHz, $CDCl_3$)

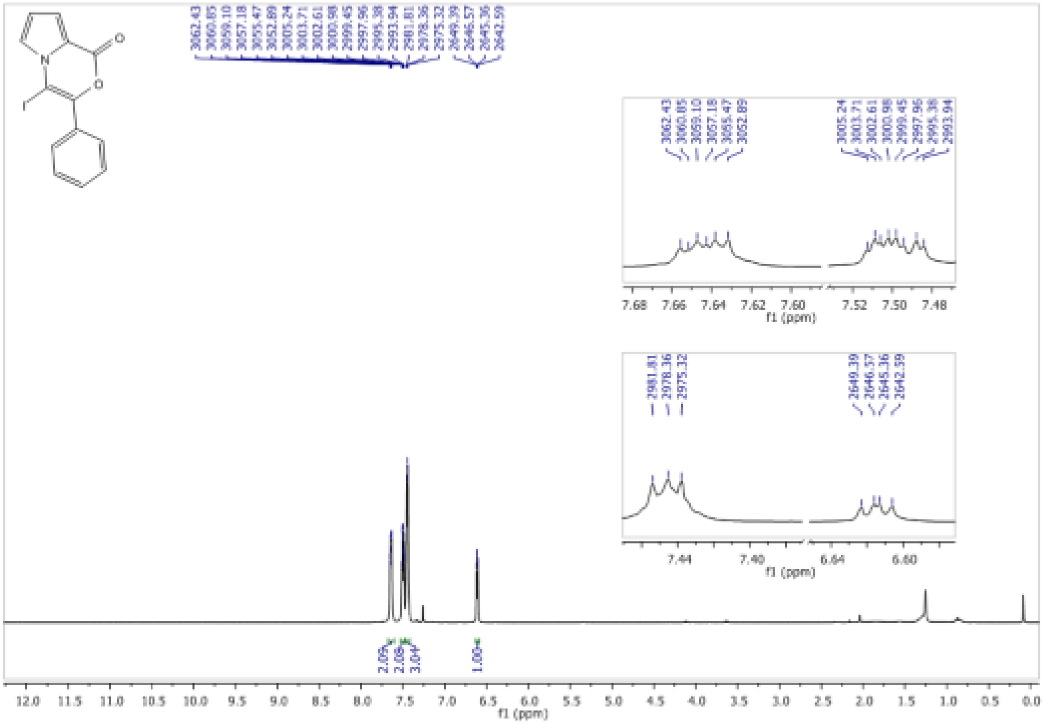

^{13}C-NMR: (100 MHz, $CDCl_3$)

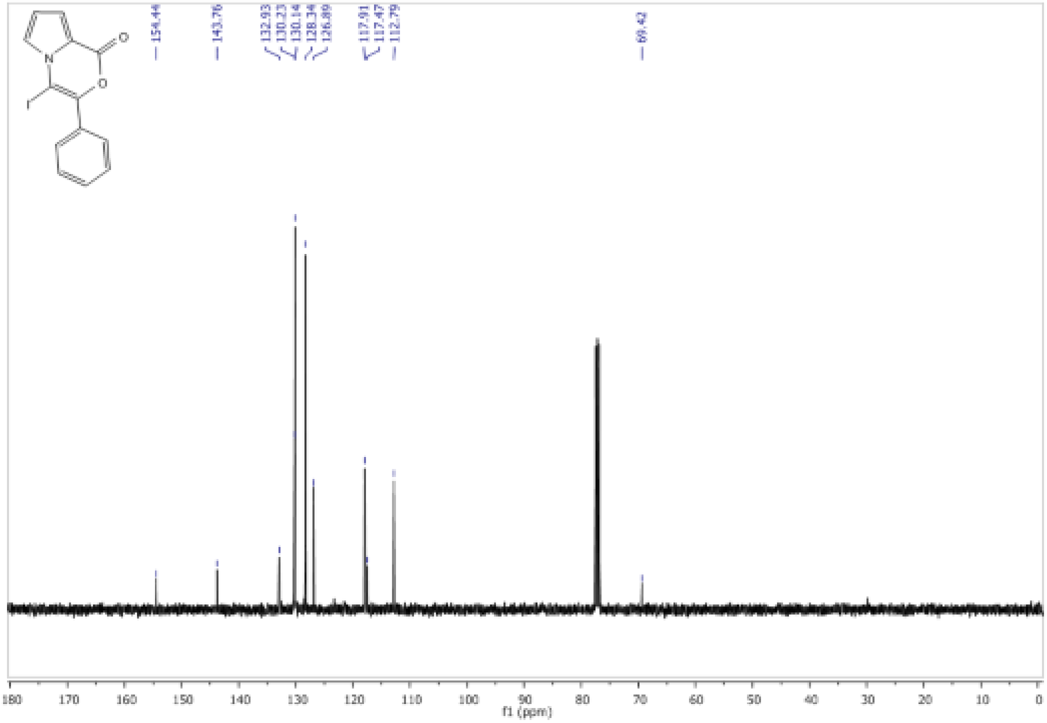

4-Iodo-3-(4-nitrophenyl)-1*H*-pyrrolo[2,1-*c*][1,4]oxazin-1-one (**19b**).

^{1}H-NMR: (400 MHz, $CDCl_3$)

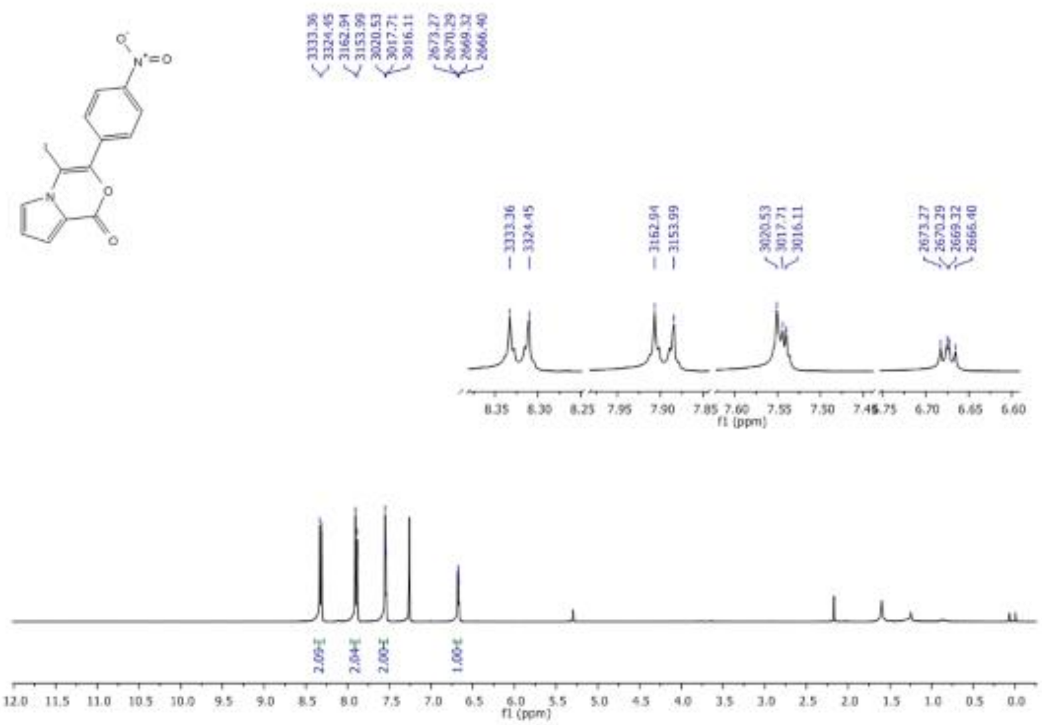

^{13}C-NMR: (100 MHz, $CDCl_3$)

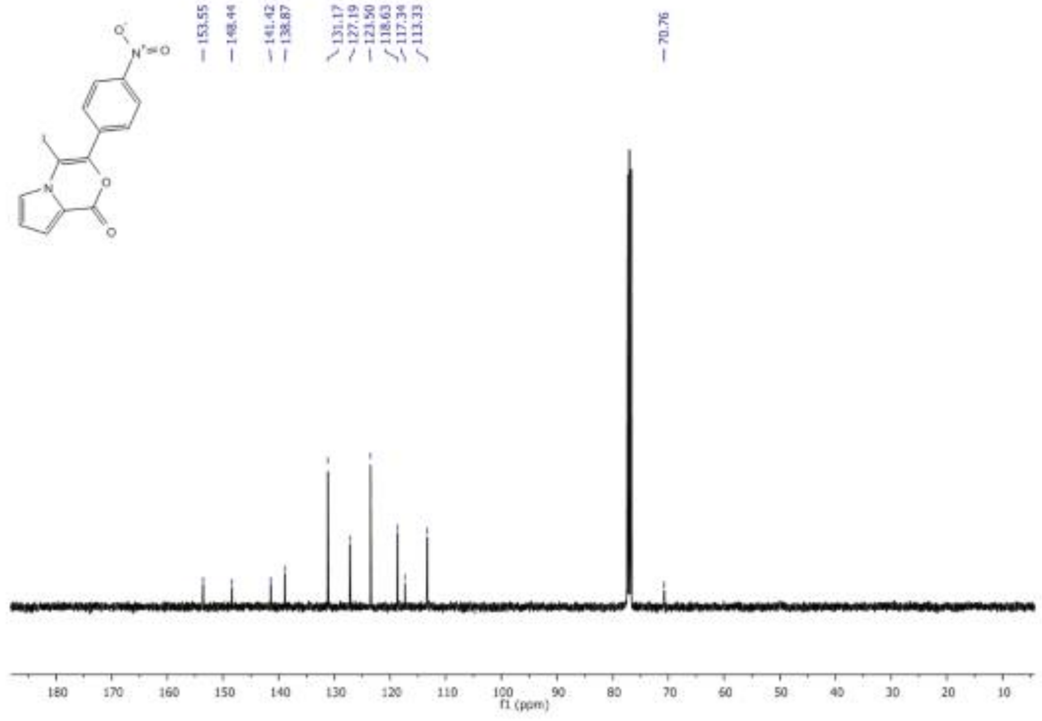

4-Iodo-3-butyl-1*H*-pyrrolo[2,1-*c*][1,4]oxazin-1-one (**19d**).

^{1}H-NMR: (400 MHz, $CDCl_3$)

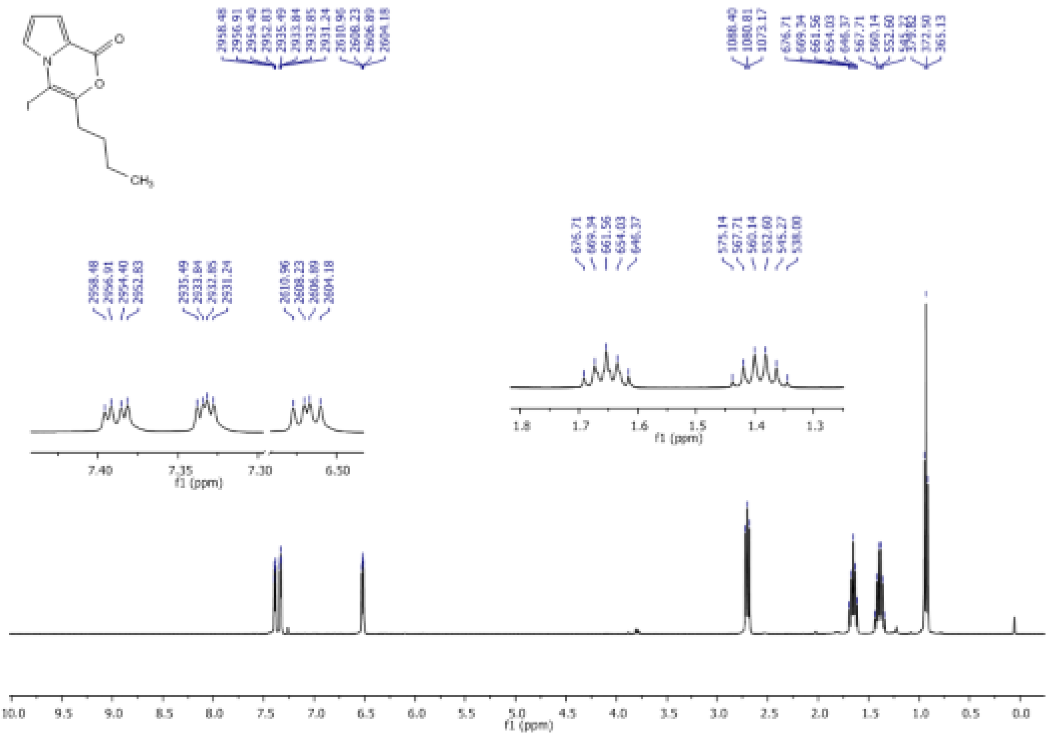

^{13}C-NMR: (100 MHz, $CDCl_3$)

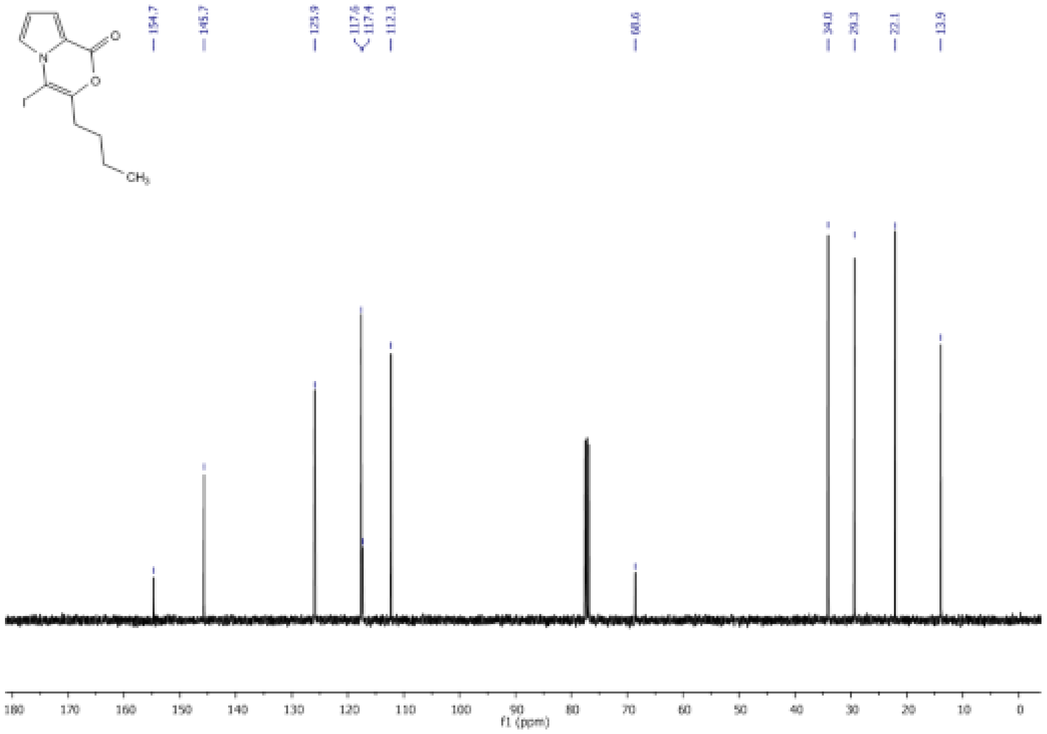

THEORETICAL CALCULATIONS

Methodology

Frequency calculations and geometrical optimizations of reactants, transition states (TS) and products were performed at the polarizable continuum model [9] (PCM) in dichloromethane with the M06 [10] method using the GEN basis set combination 6-31+G(d) and LANL2DZ (I) in Gaussian 09 [11]. The intrinsic reaction coordinates [11] (IRC) were computed to make sure that each transition state connects the corresponding reactant and the product in dichloromethane. The total electronic energies including zero point energy corrections, enthalpy corrections and Gibbs free energy corrections were extracted from the output of the frequency calculations in dichloromethane.

Table 1. Absolute energies of optimized structures in dichloromethane (M06/6-31+G(d)/ LANL2DZ)

**Compound No**	**Eel^{a}+ZPE^{b} [au]**	**Eel+ΔH^{c} [au]**	**Eel+ΔG^{d} [au]**	**Imaginary Frequency [*i*]**
**RC(20)**	-755.668121	-755.650390	-755.716279	-
**TS1**	-755.667331	-755.650552	-755.713717	-166.83
**PC(21)**	-755.681364	-755.664753	-755.726242	-
**RC(21+I^{-})**	-767.225025	-767.206051	-767.275854	-
**TS2**	-767.224394	-767.205757	-767.275350	-405.45
**PC2 (19a +CH_3I)**	-767.292892	-767.274548	-767.342293	-

[a]Eel = Total electronic energy
[b]ZPE = Zero point energy correction
[c]H = Enthalpy correction
[d]G = Gibbs free energy correction

Cartesian Coordinates for the Optimized Structures:

Structure No: RC(20) (M06/6-31+G(d)/LANL2DZ)

	X	Y	Z
C	-3.118815	-0.277407	-0.000252
C	-4.093461	0.679980	-0.000007
C	-3.451079	1.938540	0.000285
C	-2.093225	1.731769	0.000053
N	-1.873922	0.335533	-0.000280

H	-3.207160	-1.355705	-0.000439
H	-5.159395	0.490052	-0.000082
H	-3.914642	2.918247	0.000504
C	-1.093522	2.783221	0.000155
O	-1.353105	3.966559	0.001042
C	1.222266	3.290770	-0.000126
H	1.151664	3.909424	-0.898584
H	2.159961	2.729600	0.001314
H	1.149426	3.910415	0.897448
C	-0.667332	-0.333804	-0.000317
C	0.574536	0.063927	-0.000677
C	1.951748	0.101050	-0.000255
C	2.657295	0.131511	1.233732
C	2.657922	0.132396	-1.233879
C	4.036245	0.180929	1.223695
H	2.094859	0.113789	2.165310
C	4.036860	0.181827	-1.223138
H	2.095906	0.115363	-2.165723
C	4.718867	0.206276	0.000464
H	4.592118	0.200204	2.157451
H	4.593212	0.201796	-2.156594
H	5.806331	0.245751	0.000759
O	0.171585	2.311595	-0.000929
I	-0.619292	-2.470166	0.000025

Structure No: TS1 (M06/6-31+G(d)/LANL2DZ)

	X	Y	Z
C	-3.110218	-0.573041	-0.027443
C	-4.176216	0.287814	-0.011401
C	-3.663807	1.602998	0.010980
C	-2.290558	1.520644	0.006571
N	-1.938322	0.159512	-0.017680
H	-3.092494	-1.654788	-0.045471
H	-5.218302	-0.005865	-0.015793
H	-4.224325	2.530599	0.025468
C	-1.365930	2.622432	0.012311
O	-1.655567	3.792717	0.046447
C	0.939266	3.272799	-0.039143
H	0.748822	3.927047	-0.891918
H	1.911932	2.789058	-0.140099
H	0.877752	3.823581	0.901831
C	-0.651492	-0.340869	-0.012616
C	0.493022	0.306048	-0.013341
C	1.891927	0.340293	-0.004747
C	2.585557	0.393900	1.227138

C	2.601378	0.351465	-1.228570
C	3.967660	0.449345	1.226407
H	2.019604	0.390141	2.157336
C	3.983492	0.406678	-1.212087
H	2.047489	0.315627	-2.165333
C	4.660092	0.458724	0.011054
H	4.513946	0.486961	2.165596
H	4.542254	0.411417	-2.144614
H	5.747223	0.506053	0.017309
O	-0.049675	2.215759	-0.033274
I	-0.336769	-2.439615	0.009120

Structure No: PC(21) (M06/6-31+G(d)/LANL2DZ)

	X	Y	Z
C	-2.928411	-1.195955	-0.082075
C	-4.180624	-0.612991	0.022254
C	-4.002901	0.769716	0.127004
C	-2.633265	1.005897	0.079817
N	-1.976353	-0.222414	-0.051667
H	-2.657095	-2.240887	-0.170074
H	-5.120434	-1.150427	0.020184
H	-4.764007	1.535983	0.218294
C	-1.979365	2.246042	0.087210
O	-2.328951	3.373962	0.168148
C	0.330513	3.220121	-0.539700
H	-0.376130	4.047461	-0.530965
H	0.690701	3.001029	-1.545298
H	1.136428	3.380088	0.174717
C	-0.586057	-0.332307	-0.063123
C	0.203441	0.749538	-0.079082
C	1.669669	0.793661	-0.010601
C	2.293253	1.051399	1.215607
C	2.435646	0.566722	-1.158645
C	3.681514	1.080609	1.289294
H	1.686870	1.220353	2.105709
C	3.823793	0.594000	-1.077036
H	1.939339	0.359365	-2.107174
C	4.444565	0.851743	0.144476
H	4.168671	1.275298	2.242607
H	4.421843	0.411086	-1.967307
H	5.530958	0.871244	0.206219
O	-0.443417	2.040624	-0.080351
I	0.262748	-2.249048	0.024151

Structure No: RC(21+I$^-$) (M06/6-31+G(d)/LANL2DZ)

	X	Y	Z
C	-4.193960	1.421530	-0.324949
C	-4.515597	2.764530	-0.222722
C	-3.339901	3.467368	0.065853
C	-2.315282	2.532310	0.128715
N	-2.856479	1.266741	-0.116191
H	-4.829202	0.568834	-0.530462
H	-5.507310	3.180413	-0.349033
H	-3.217645	4.535213	0.205038
C	-0.941849	2.762518	0.333007
O	-0.303357	3.741102	0.544054
C	1.345011	1.576070	0.086434
H	1.540779	2.641238	-0.017964
H	1.614397	1.008514	-0.802821
H	1.781271	1.160894	0.993844
C	-2.085646	0.106102	-0.060245
C	-0.758031	0.157846	0.108006
C	0.180994	-0.962383	0.240088
C	0.596649	-1.374166	1.510952
C	0.661461	-1.607481	-0.904537
C	1.493124	-2.430274	1.631593
H	0.214239	-0.866454	2.397067
C	1.556111	-2.663273	-0.776160
H	0.329920	-1.276283	-1.889177
C	1.972592	-3.070873	0.489978
H	1.821221	-2.752334	2.617707
H	1.936833	-3.161735	-1.665177
H	2.680983	-3.891147	0.588223
O	-0.155785	1.466541	0.219355
I	-3.059215	-1.749240	-0.155233
I	4.707686	0.470854	-0.234904

Structure No: TS2 (M06/6-31+G(d)/LANL2DZ)

	X	Y	Z
C	-3.965131	1.800245	-0.279342
C	-4.080935	3.176174	-0.179737
C	-2.810413	3.697249	0.103750
C	-1.938339	2.621627	0.169658
N	-2.663884	1.451942	-0.070930

H	-4.720493	1.049494	-0.476748
H	-5.000845	3.734261	-0.302344
H	-2.531593	4.735198	0.243064
C	-0.540744	2.615904	0.397777
O	0.215045	3.519387	0.607722
C	1.712587	1.162570	0.133696
H	1.986269	2.213291	0.138129
H	1.758738	0.651109	-0.821611
H	1.986578	0.590610	1.012347
C	-2.056199	0.198197	-0.029178
C	-0.735493	0.083007	0.171750
C	0.049237	-1.154688	0.294005
C	0.488425	-1.573463	1.555059
C	0.381930	-1.892553	-0.846306
C	1.261381	-2.723657	1.669925
H	0.225307	-0.992394	2.439816
C	1.149630	-3.046150	-0.724301
H	0.036134	-1.556404	-1.824266
C	1.591569	-3.458277	0.531578
H	1.607762	-3.047575	2.649318
H	1.411515	-3.618565	-1.611852
H	2.201172	-4.355205	0.623339
O	0.033726	1.274898	0.344571
I	-3.267857	-1.502307	-0.222175
I	4.676375	0.427613	-0.287279

Structure No: PC(19a+CH_3I) (M06/6-31+G(d)/LANL2DZ)

	X	Y	Z
C	-4.028122	1.383513	-0.839218
C	-4.380739	2.719111	-0.745327
C	-3.378070	3.382159	-0.016850
C	-2.428687	2.434012	0.320419
N	-2.840036	1.202245	-0.193358
H	-4.536520	0.556694	-1.319839
H	-5.277738	3.157238	-1.166628
H	-3.330852	4.432713	0.245558
C	-1.200568	2.545230	1.049595
O	-0.745328	3.552912	1.547729
C	2.723994	1.344482	0.209448
H	3.018871	2.311668	0.618869
H	1.927605	1.455812	-0.528028
H	2.430565	0.651924	1.000771
C	-2.064405	0.056410	0.004950
C	-0.895165	0.168974	0.666230
C	0.079670	-0.891807	0.967097

C	0.464720	-1.105132	2.295575
C	0.689458	-1.626110	-0.055204
C	1.432322	-2.057691	2.598102
H	0.001563	-0.520377	3.089578
C	1.658715	-2.576641	0.250681
H	0.420935	-1.433557	-1.094107
C	2.031133	-2.794017	1.576163
H	1.722122	-2.223476	3.634261
H	2.140645	-3.133399	-0.551407
H	2.793781	-3.534168	1.812386
O	-0.481044	1.380545	1.174957
I	-2.855432	-1.779643	-0.631385
I	4.443256	0.506069	-0.799924

X-ray Crystallography: The single-crystal X-ray crystallographic diffraction data were collected at 293 K with a Rigaku R-axis Rapid-S IP-detector diffractometer with graphite-monochromated Mo-K_{α} radiation (λ = 0.71073 Å). Suitable single crystals of **12c** and **19c** were mounted on the tip of a glass fiber with silicone grease and transferred to the diffractometer for data collection. The collection of frames of data, indexing of reflections, and determination of the lattice parameters and the integration of the intensity of the reflections were performed with the CrystalClear (Rigaku/MSC Inc., 2005) software [12]. All of the structures were solved by direct methods with SHELXS-97 [13] and refined with SHELXL-97 [13]. The non-hydrogen atoms were refined anisotropically, and the hydrogen atoms were fixed at calculated positions and refined isotropically with a riding model. The final difference Fourier maps showed no peaks of chemical significance. CCDC 1523007(**19a**) and 1524965 (**12c**) contains the supplementary crystallographic data for this paper. These data can be obtained free of charge from The Cambridge Crystallographic Data Centre.

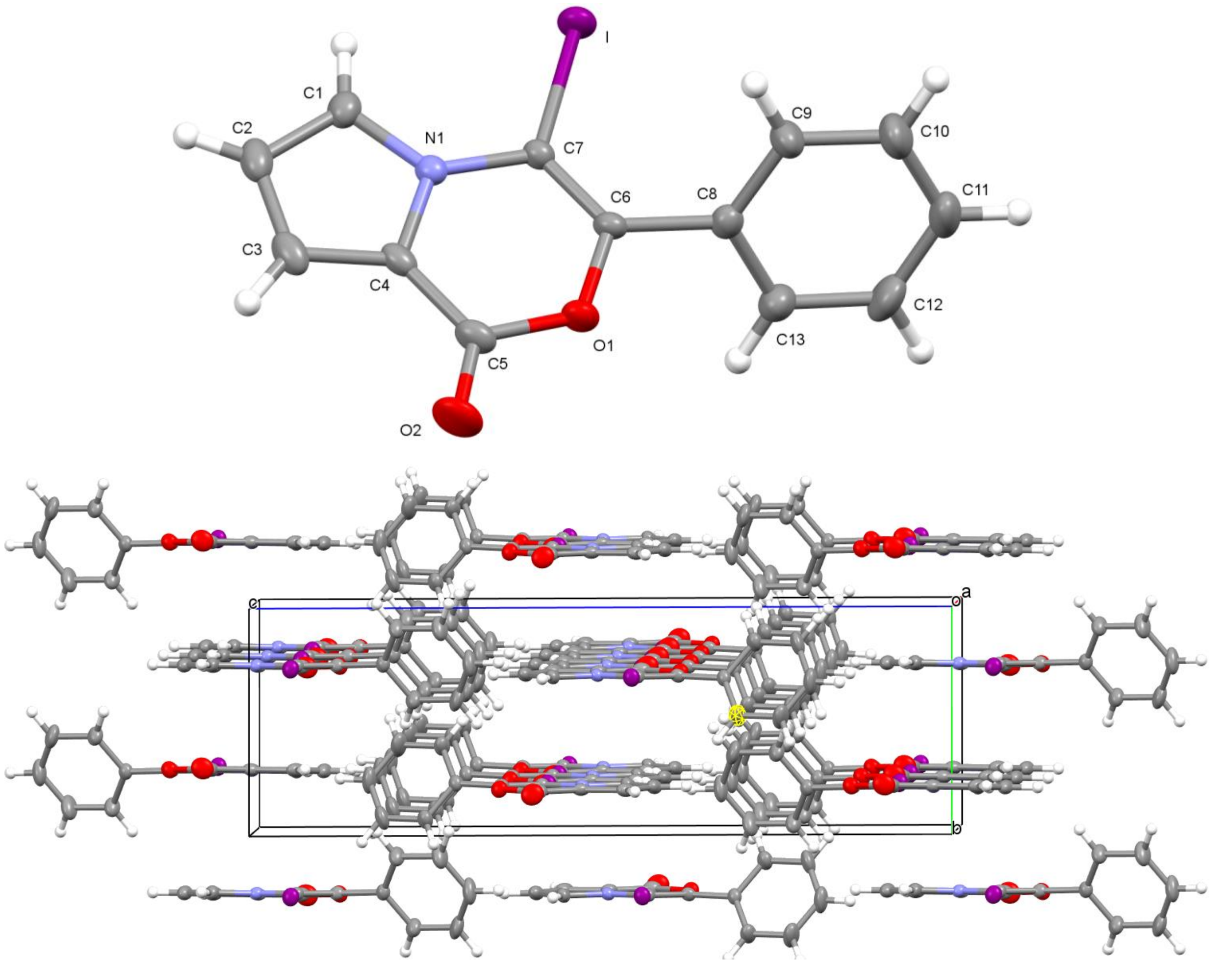

Figure 1: (Up) X-ray view of the molecule (**19c**), shown with 40% probability displacement ellipsoids. (Down) the unit cell viewed along the *a*-axis

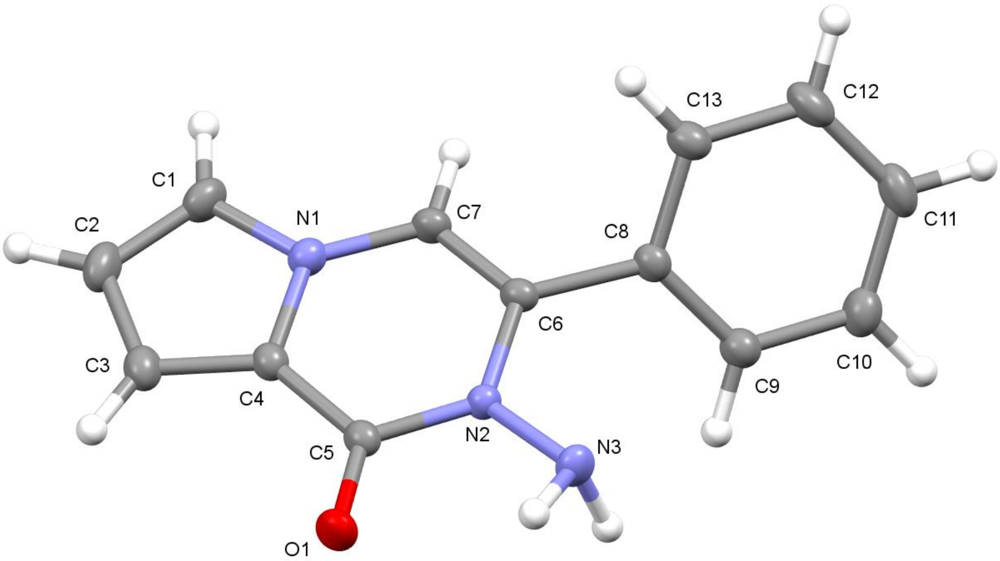

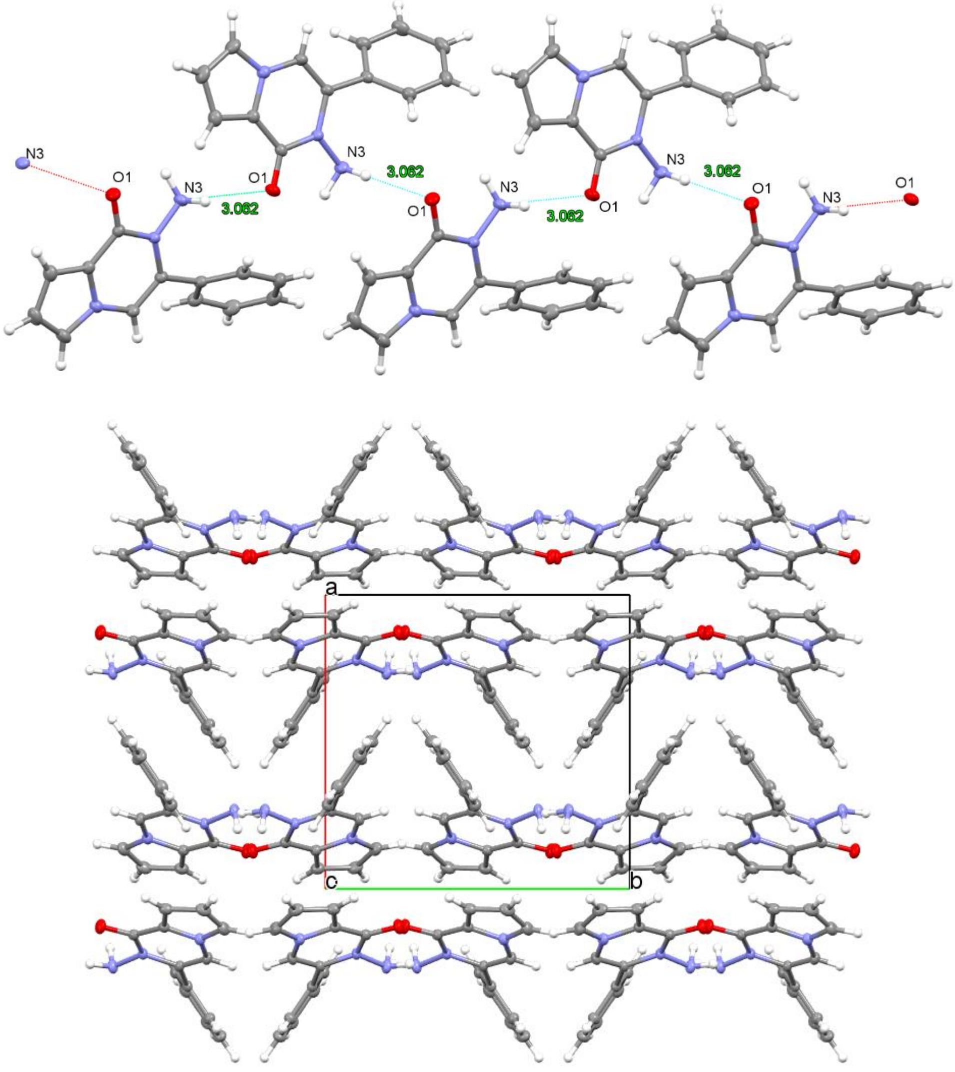

Figure 2. (Up) X-ray view of the molecule (**12c**), shown with 40% probability displacement ellipsoids. (Down) H-bonding geometry and the unit cell viewed along the *c*-axis

REFERENCES

1 Taskaya, S.; Menges, N.; Balci, M. *Beilstein J. Org. Chem.* **2015,** *11,* 897.

2 Hewlett, N. M.; Tepe, J. J. *Org. Lett***. 2011,** *13,* 4550.

3 Schmuck, C.; Bickert, V.; Merschky, M.; Geiger, L.; Rupprecht, D.; Dudaczek, J.; Wich, P.; Rehm, T.; Machon, U. *Eur. J. Org. Chem.* **2008,** 324.

4 Dateer, R. B.; Shaibu, B. S.; Liu, R.-S. *Angew. Chem., Int. Ed.* **2012,** *51,* 113.

5 Zhou, N.; Wang, L.; Thompson, D. W.; Zhao, Y. *Tetrahedron* **2011,** *67,* 125.

6 Takahashi, S.; Kuroyama, Y.; Sonogashira, K.; Hagihara, N. *Synthesis* **1980,** 627.

7 Nie, X.; Wang, G. *J. Org. Chem.* **2006,** *71,* 4734.

8 Hofmeister, H.; Annen, K.; Laurent, H.; Wiechert, R. *Angew. Chem., Int. Ed. Engl.* **1984,** *23,* 727.

9 S. Miertus, E. Scrocco and J. Tomasi, *Chem. Phys.*, 1981, **55**, 117.

10 Y. Zhao and D. G. Truhlar, *Theor. Chem. Acc.*, 2008, **120**, 215.

11 Gaussian 09, Revision E.01, M. J. Frisch, G. W. Trucks, H. B. Schlegel, G. E. Scuseria, M. A. Robb, J. R. Cheeseman, G. Scalmani, V. Barone, B. Mennucci, G. A. Petersson, H. Nakatsuji, M. Caricato, X. Li, H. P. Hratchian, A. F. Izmaylov, J. Bloino, G. Zheng, J. L.

Sonnenberg, M. Hada, M. Ehara, K. Toyota, R. Fukuda, J. Hasegawa, M. Ishida, T. Nakajima, Y. Honda, O. Kitao, H. Nakai, T. Vreven, J. A. Montgomery, Jr., J. E. Peralta, F. Ogliaro, M. Bearpark, J. J. Heyd, E. Brothers, K. N. Kudin, V. N. Staroverov, R. Kobayashi, J. Normand, K. Raghavachari, A. Rendell, J. C. Burant, S. S. Iyengar, J. Tomasi, M. Cossi, N. Rega, J. M. Millam, M. Klene, J. E. Knox, J. B. Cross, V. Bakken, C. Adamo, J. Jaramillo, R. Gomperts, R. E. Stratmann, O. Yazyev, A. J. Austin, R. Cammi, C. Pomelli, J. W. Ochterski, R. L. Martin, K. Morokuma, V. G. Zakrzewski, G. A. Voth, P. Salvador, J. J. Dannenberg, S. Dapprich, A. D. Daniels, Ö. Farkas, J. B. Foresman, J. V. Ortiz, J. Cioslowski and D. J. Fox, Gaussian, Inc., Wallingford CT, 2009.Gonzalez, C.; Schlegel, H. B. *J. Chem. Phys.* **1989**, *90*, 2154-2161.

12 Rigaku/MSC, Inc., 9009 new Trails Drive, The Woodlands, TX.G.M. Shedrick.

13 SHELXS97 and SHELXL97, University of Göttingen (1997)
